# Genome-Wide Expression Analysis of Root Tips in Contrasting Rice Genotypes Revealed Novel Candidate Genes for Water Stress Adaptation

**DOI:** 10.3389/fpls.2022.792079

**Published:** 2022-02-21

**Authors:** Somayeh Abdirad, Mohammad Reza Ghaffari, Ahmad Majd, Saeed Irian, Armin Soleymaniniya, Parisa Daryani, Parisa Koobaz, Zahra-Sadat Shobbar, Laleh Karimi Farsad, Parisa Yazdanpanah, Amirhossein Sadri, Mehdi Mirzaei, Zahra Ghorbanzadeh, Mehrbano Kazemi, Naghmeh Hadidi, Paul A. Haynes, Ghasem Hosseini Salekdeh

**Affiliations:** ^1^Department of Systems and Synthetic Biology, Agricultural Biotechnology Research Institute of Iran, Agricultural Research, Education and Extension Organization, Karaj, Iran; ^2^Department of Plant Biology, Faculty of Biological Sciences, Kharazmi University, Tehran, Iran; ^3^Department of Cell and Molecular Biology, Faculty of Biological Sciences, Kharazmi University, Tehran, Iran; ^4^Department of Biotechnology, University of Tehran, Tehran, Iran; ^5^Department of Molecular Physiology, Agricultural Biotechnology Research Institute of Iran, Agricultural Research, Education and Extension Organization, Karaj, Iran; ^6^Faculty of Medicine, Health and Human Sciences, Macquarie University, Sydney, NSW, Australia; ^7^Department of Clinical Research and Electronic Microscope, Pasteur Institute of Iran, Tehran, Iran; ^8^Department of Molecular Sciences, Macquarie University, Sydney, NSW, Australia

**Keywords:** rice, root tip, water stress, root system architecture, RNAseq, transcriptome

## Abstract

Root system architecture (RSA) is an important agronomic trait with vital roles in plant productivity under water stress conditions. A deep and branched root system may help plants to avoid water stress by enabling them to acquire more water and nutrient resources. Nevertheless, our knowledge of the genetics and molecular control mechanisms of RSA is still relatively limited. In this study, we analyzed the transcriptome response of root tips to water stress in two well-known genotypes of rice: IR64, a high-yielding lowland genotype, which represents a drought-susceptible and shallow-rooting genotype; and Azucena, a traditional, upland, drought-tolerant and deep-rooting genotype. We collected samples from three zones (Z) of root tip: two consecutive 5 mm sections (Z1 and Z2) and the following next 10 mm section (Z3), which mainly includes meristematic and maturation regions. Our results showed that Z1 of Azucena was enriched for genes involved in cell cycle and division and root growth and development whereas in IR64 root, responses to oxidative stress were strongly enriched. While the expansion of the lateral root system was used as a strategy by both genotypes when facing water shortage, it was more pronounced in Azucena. Our results also suggested that by enhancing meristematic cell wall thickening for insulation purposes as a means of confronting stress, the sensitive IR64 genotype may have reduced its capacity for root elongation to extract water from deeper layers of the soil. Furthermore, several members of gene families such as *NAC*, *AP2/ERF*, *AUX/IAA*, *EXPANSIN*, *WRKY*, and *MYB* emerged as main players in RSA and drought adaptation. We also found that *HSP* and *HSF* gene families participated in oxidative stress inhibition in IR64 root tip. Meta-quantitative trait loci (QTL) analysis revealed that 288 differentially expressed genes were colocalized with RSA QTLs previously reported under drought and normal conditions. This finding warrants further research into their possible roles in drought adaptation. Overall, our analyses presented several major molecular differences between Azucena and IR64, which may partly explain their differential root growth responses to water stress. It appears that Azucena avoided water stress through enhancing growth and root exploration to access water, whereas IR64 might mainly rely on cell insulation to maintain water and antioxidant system to withstand stress. We identified a large number of novel RSA and drought associated candidate genes, which should encourage further exploration of their potential to enhance drought adaptation in rice.

## Introduction

Rice (*Oryza sativa* L.) is a staple food for nearly half of the world’s population (Bhuiyan, 1992). Drought, estimated to affect a large portion of the world’s rice production (Lesk et al., 2016), is defined as a decrease in water input into an ecosystem over time and being sufficient to result in soil water deficit (Gilbert and Medina, 2016; Abdirad et al., 2020). Water-deficit stress limits crop growth and development, eventually leading to a sharp decline in productivity (Michaletti et al., 2018). Therefore, developing plants capable of tolerating conditions of water deficit while maintaining a high yield is of great interest (Salekdeh et al., 2009).

Root system architecture (RSA) is an important agronomic trait with vital roles in plant adaptation and productivity in water-limited environments. A deep, thick, and branched root system may help plants to avoid water stress by enabling them to acquire more water and nutrient resources (Ye et al., 2018). Deep rooting is a complex trait affected by growth angle and root length (Araki et al., 2002; Uga et al., 2013). Other deep-rooting determinants include root thickness and penetrability, with thicker roots penetrating deeper through soil-layers (Yu et al., 1995; Zheng et al., 2000). Also, branched and proliferate rooting, mainly determined by the number and length of adventitious and lateral roots (LRs), is considered as an important contributor to water uptake efficiency under water-deficit conditions (Ye et al., 2018).

From a developmental biological perspective, a developmental gradient is apparent at the growing root tips from meristematic to mature zones, with each responding differentially to water stress and performing specific functions during RSA formation. The major longitudinal regions of a typical root include: The meristematic region wherein cell division and cell production originate; the elongation zone wherein the growing cells elongate and lose the power of division; the zone of maturation with completely differentiated cells wherein the root hairs and LRs initiate and emerge, and specialized tissues are differentiated.

Lowland rice, accounting for 80% of the total rice produced worldwide, is generally grown under flooded conditions, and is susceptible to drought due to its shallow root distribution and limited capacity to extract water from deep soil layers (Kondo et al., 2003). Upland rice, however, is mainly planted in unbounded fields without irrigation facilities, and thus by being exposed to drought has accumulated a relatively greater morphological and genetic variations contributing to its drought resistant traits, including the development of deeper roots for enhanced water uptake (Bernier et al., 2008).

A strategy envisaged to enhance drought resistance in lowland rice cultivars would be to introduce the deep rooting traits of the upland rice into the commercial lowland rice cultivars. Such cultivars would also be better suited to the Alternate Wetting and Drying irrigation systems, wherein the field is not continuously flooded. This involves periodic introduction of unsaturated soil conditions during the growing season, and has been shown to decrease water demand for irrigation up to 30%, and reduce greenhouse gas emissions without reducing crop yields (Carrijo et al., 2017).

Progress has been made in detecting large effect quantitative trait loci (QTL) conferring the ratio of deep roots in rice (Uga et al., 2011; Lou et al., 2015). The root transcriptomes of the extreme shallow or deep-rooting genotypes, surveyed under abiotic stresses have led to the identification of numerous stress-responsive genes and pathways (Mehra et al., 2016; Singh et al., 2016; Sinha et al., 2018; Subudhi et al., 2020; Tiwari et al., 2020). However, our knowledge of the genetics and molecular control mechanisms of deep rooting in rice is still relatively limited as only a few genes involved in RSA traits have been identified. In addition, these studies by applying hydroponic media have ignored the impact of soil texture on RSA development (Mehra et al., 2016; Singh et al., 2016; Subudhi et al., 2020; Tiwari et al., 2020). Furthermore, by using whole roots, the important signaling events localized to particular areas, such as the root tips, go undetected. Root tips, by encompassing the root cap, apical meristem and elongation zones, are considered to be of great functional significance as these regions determine the fate of root traits (Uga et al., 2013; Wu et al., 2016).

The integration of RSA Meta-QTLs analysis with transcriptome profiling may assist in generating a more reliable list of potential candidate genes involved in RSA. Meta-analysis of QTLs is a powerful statistical technique for reducing the confidence interval of QTL position through refining and confirming QTL positions on a consensus map via mathematical models. In Meta-QTL analysis, a number of independent QTL studies performed across different genetic backgrounds and environments are combined to determine the number of true QTLs enabling researchers to reduce the intervals and the number of candidate genes (Coudert et al., 2010; Comas et al., 2013; Daryani et al., 2021).

Here we aimed at monitoring the transcriptome response to water stress of three different root regions in two well-known genotypes of rice, namely IR64, a high-yielding, lowland, drought-susceptible, and shallow-rooting genotype, and Azucena, a traditional, upland, drought-tolerant and deep-rooting genotype. The transcriptome data were collected from two consecutive 5 mm sections and the following next 10 mm section starting from the distal tip, namely Z1–Z3, aimed to be enriched for undifferentiated, differentiating and differentiated cells, respectively. Z1, containing meristematic and elongating cells, is the region of cell division, and elongation, and is enriched for root traits such as length, thickness and angle. In zones 2 and 3, root tissues are gradually differentiating and enriched for LR production (Takehisa et al., 2012). We then integrated the transcriptome data into Meta-QTLs, resulting in identification of a large number of novel RSA/drought associated candidate genes.

## Materials and Methods

### Sample Preparation

**FIGURE 1:**
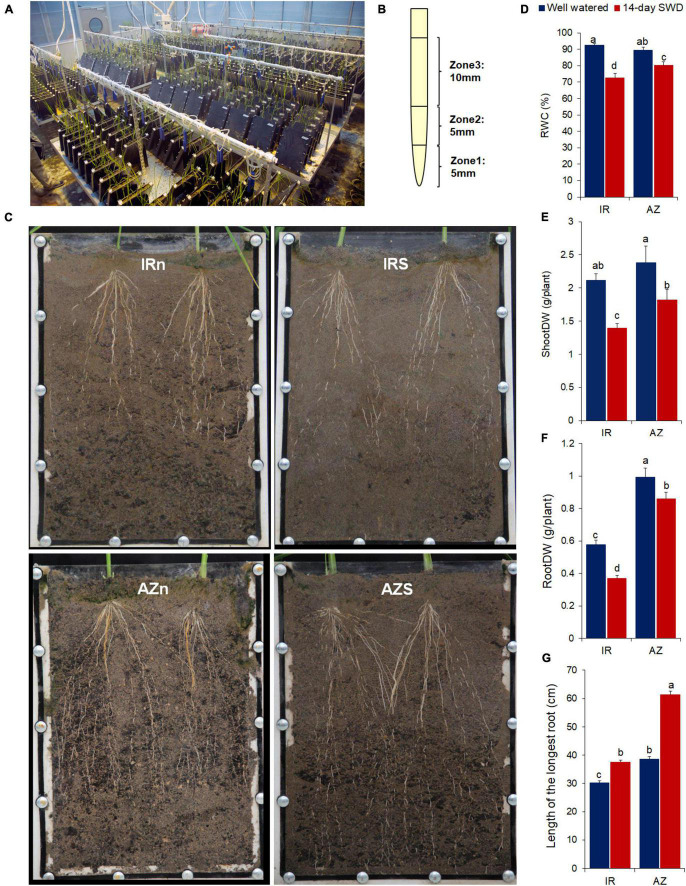
Root sampling and phenotypic responses of two contrasting rice genotypes, Azucena and IR64, to water stress. **(A)** Soil-filled root boxes containing rice plants in the greenhouse; **(B)** root tip sampling; **(C)** the root length of IR64 (top panel, IRn and IRs) and Azucena (bottom panel, AZn and AZs) under well-watered (Left panel, IRn and AZn) and 14-day water withholding (right panel, IRs and AZs) conditions, shown in the transparent root boxes; 14-day-treated root boxes were watered prior to taking photos for a better resolution; **(D)** relative water content (RWC); **(E)** shoot dry weight; **(F)** root dry weight; and **(G)** length of the longest root of two contrasting genotypes of rice, IR64 (IR), the shallow rooting and sensitive genotype and Azucena (AZ), the deep rooting and tolerant genotype, grown in soil-filled root boxes in response to a 14-day water-deficit stress compared to well-watered samples. Values represent mean ± SE from six independent samples. The different letters indicate significant difference (*p* ≤ 0.05) by Duncan’s test. **(E)** DW, dry weight; IRn, IR64 genotype under normal condition; IRs, IR64 genotype under water-deficit stress condition; AZn, Azucena genotype under normal condition; AZs, Azucena genotype under water-deficit stress condition.

Seeds of two genotypes of rice (*Oryza sativa* L.), Azucena (a japonica-type, traditional, upland, and deep-rooting genotype) and IR64 (an *indica*-type, cultivated, lowland, and shallow-rooting genotype) (Yadav et al., 1997; Shashidhar et al., 1999) were provided by the IRRI International Rice Genebank Collection in the Philippines. Sterilized seeds were germinated on water-soaked filter paper, and uniformly sized 7-day-old seedlings were cultured hydroponically in Yoshida solution (Yoshida, 1976) at 22–25°C, relative humidity of 85% and a photoperiod of 16 h for 2 weeks. Then, 20-day-old seedlings were transferred to 400 root boxes (two plants in each box) filled with a mix ratio of 1:1:2 of clay, pit, and sand, respectively (Figure 1A). The root boxes were made of plexiglass sheets with a thickness of 5 mm (the root box dimensions: 25 cm × 3 cm × 40 cm L × W × H) and placed in a greenhouse of ABRII (Agricultural Biotechnology Research Institute of Iran) (Figures 1A,C). In a randomized design (100 root boxes and 200 plants per each treatment), water stress was imposed on 35-day-old plants by withholding water for 14 days till the level of field capacity was reduced to 25–35%. The control plants were watered regularly. Field capacity was measured twice a day during the treatment period for at least 40 root boxes, randomly (Puértolas et al., 2017). The Soil moisture of the 40 root boxes were also monitored using a Soil Moisture Sensor SM150 (Delta-T Devices, Cambridge, United Kingdom). Relative water content (RWC) of fresh leaves (the last expanded leaf) was measured 14 days after water withholding using the following formula (Martinez et al., 2004): RWC = (FW – DW) 100/(SW – DW) × 100, where FW corresponds to the leaf fresh weight, DW to the leaf dry weight (determined after 48 h in an oven at 80°C), and SW to the leaf water-saturated weight measured after 24 h of saturation on deionized water in the dark.

To assay the dry weight, shoots and roots were separated and dried in an oven at 110°C for 2 days (Morant-Manceau et al., 2004). Also, the length of the longest root was measured. The collected data (six biological replicates) were subjected to analysis of variance (ANOVA), and the mean differences were compared using Duncan’s range test at *P* values ≤ 0.05. All calculations were performed using SPSS software, version 19.

Fourteen days after stress initiation, crown root tips were dissected from both well-watered and stressed 50-day-old plants and were immediately snap-frozen in liquid nitrogen, and then stored at −80°C. Root tip samples were collected from three zones: two consecutive 5 mm sections (Z1 and Z2) and the following next 10 mm section (Z3) (Figure 1B). Only the root tips located in one third of the bottom of root boxes, were considered for sampling. Three biological replicates were considered for each genotype/zone/condition. Biological replicates were collected on different dates (12 days) and pooled, separately, to allow for day-to-day variation within each genotype/zone/condition and other environmental variables. Approximately root tip sections of 70 plants were pooled as a biological replicate.

### Microscopic Analysis

Root samples were cut into small pieces of 2.5 mm (serial sections), fixed in 4% glutaraldehyde (in PBS buffer), dried under vacuum for 3 h, shaken for 2 h, washed in PBS buffer (pH 7.2, 100 mM) six times and then kept in PBS buffer at 4°C overnight. The fixed samples were post fixed in 1% osmium tetroxide (OsO4) (in PBS buffer) for 2 h, and washed in PBS buffer and distilled water, three times each, respectively. Then, they were dehydrated stepwise in 5, 10, 30, 50, 70, 80, 90, 95, and 100% acetone for 15 min in each step and then twice in 100% acetone again for 20 min. Finally, the samples were embedded stepwise in different mixtures of Spurs resin/100% acetone, 25%/75%, 50%/50%, 75%/25% for 4, 1, and 4 h, respectively, followed by pure Spurs resin, three times and for 4, 12, and 24 h and oven-cured at 65°C for 48 h. For light microscopy, serial semi thin transverse and longitudinal sections (1–2 μm) were obtained from resin blocks cutting. Then the sections were mounted on glass slides, stained with 0.05% methylene blue (w/v), and observed and recorded with a light microscope (Olympus BX53, Tokyo, Japan). For transmission electron microscopy (TEM), the blocks were cut into ultrathin sections of 40–50 nm thick with an RMC MT-7000 Ultramicrotome (Tucson, AZ, United States) by a glass knife, and then stained with 2% uranyl acetate and 2% lead citrate, respectively (Bozzola and Russell, 1999). Ultimately, the ultrastructural analysis and photography were performed using a Zeiss EM900 Transmission Electron Microscope (Carl Zeiss, Oberkochen, Germany).

### RNA Extraction, RNA-Seq Library Construction and Sequencing

A total of 24 root samples were collected for RNAseq profiling from three different zones of the two contrasting genotypes of rice under two different growth conditions with two independent biological replicates for each treatment. Total RNA was isolated from 100 mg root tissue using the Invitrogen TRIzol Reagent (Thermo Fisher Scientific, Waltham, MA, United States) as previously described (Mardi et al., 2015).

The RNA was analyzed for quality and concentration determination using a Nanodrop 2000 spectrophotometer (NP80 NanoPhotometer, IMPLEN, Munich, Germany) and an Agilent Technologies 2100 Bio Analyzer. All the RNA samples had A260/A280 nm ratios between 2 and 2.1 and RNA integrity number (RIN) between 9 and 10.

### RNA-Seq Data Processing

RNA-seq (Illumina Hiseq 2500 system with 150-bp paired end reads, Beijing Genomics Institute, BGI) were performed on 24 samples, and approximately 15–22 million read pairs were collected for each sample (a total of 70 Gb data). The sequencing data has been submitted to SRA of NCBI with accession no. PRJNA716593.

Quality assessment of the raw sequencing reads using FASTQC tool showed that all data were clean and did not require trimming. The clean reads were aligned to the *O. sativa* cv. Nipponbare (ssp. *japonica*) reference genome sequences IRGSP 1.0^1^ using Hisat2 (Kim et al., 2015; Pertea et al., 2016). Then, for assembly, Cufflinks utility (Trapnell et al., 2012) was used on the Hisat2-generated alignment and the transcriptome assembly was performed by cuffmerge meta-assembler. The Cuffcompare utility was used to identify novel transcripts.

Raw read counts were normalized by library size and accounted for the effect of extremely differentially expressed genes using the trimmed Mean of the *M*-values (TMM) normalization approach (Robinson and Oshlack, 2010) to give counts per million reads mapped (CPM) values for each gene in each sample. CPM values were then normalized by gene coding sequence (CDS) lengths to give fragments per kilo base of exon per million reads (FPKM) values.

The silent genes were selected where there were no doublet sets of biological replicates with FPKM values of more than one in all 24 samples. The expressed genes were detected where at least both replicates of one doublet set had FPKM values of more than one. The constitutive, intermediate frequency and low frequency genes were identified from expressed genes as having FPKM > 1 in more than 80%, between 20 and 80%, and less than 20% of samples, respectively. Genes actively expressed in all replicates of the three zones of one genotype, but not expressed in the other, were referred to as genotype-specific genes. Similar approaches were used to determine-zone specific and condition-specific genes.

### Identification of Differentially Expressed Genes

Genes with differential expression between control and stress (stress vs. control) were identified for each zone in each genotype using Cuffdiff (FDR adjusted *p*-value < 0.05) (Trapnell et al., 2012), and the fold changes (FC) were calculated for each gene in response to stress. Differentially expressed genes (DEGs) were further classified into different fold-change categories (i.e., 2 > FC, 4 > FC > 2, 8 > FC > 4, 16 > FC > 8, FC > 16) considering their directions for upregulation and downregulation. An upset plot (showing the intersections of DEGs sets), generated by Upset package in R, was performed on total DEGs to identify general, zone-specific and genotype-specific DEGs.

### Gene Ontology Enrichment Analysis

Gene Ontology (GO) enrichment analysis was performed using Cytoscape software (version 3.8.2) with the ClueGO V2.5.7 plug-in (Bindea et al., 2009). The *P*-value was calculated by two-sided hypergeometric tests, and Benjamini–Hochberg adjustment was used for multiple test correction. GO terms with a *P*-value < 0.05 were considered significant.

### Finding Gene Family Members and Genes Known to Be Involved in Root Development

The members of 24 important gene families, *NAC domain transcription factor* (TF), *APETALA2/Ethylene-Responsive Factor* (*AP2/ERF*), *Auxin/Indole-3-Acetic Acid* (*Aux/IAA*), *auxin response factor* (*ARF*), *WRKY TF* (*WRKY*), *MYB TF*, *MYB-Related TF*, *EXPANSIN*, *GRAS TF*, *late embryogenesis abundant* (*LEA*) *protein*, *heat-shock protein* (*HSP*), *heat shock factor* (*HSF*) *TF*, *ARID*, *basic helix-loop-helix* (*bHLH*) *TF*, *basic leucine zipper* (*bZIP*), *lateral organ boundaries domain* (*LOB*), *WUSCHEL-related homeobox* (*WOX*) *TF*, *ethylene insensitive3-like* (*EIL*) *TF*, *growth-regulating factor* (*GRF*) *TF*, *golden 2-like* (*G2-like*) *TF*, *TIP*, and *PIP aquaporins*, *SWEET*, *response regulator* (*RR*), and *MADS-box* were collected by searching through the following databases: fun rice genes^2^, oryza base^3^, Rapdb^4^, Rice phylogenomics^5^, and uniprot^6^.

An expanded list of previously reported genes involved in root elongation, lateral root formation, root growth and development, and root cell wall biogenesis and modification was also prepared from the literature and by searching through the same databases using relevant keywords.

To identify gene family members with significant interactions between genotypes and conditions, the expression level of members was extracted from the transcriptome profiling data followed by the statistical analysis of their expression pattern using a two-way analysis of variance (ANOVA) for each zone, separately (*p*-value cutoff of <0.05).

### Quantitative Trait Loci Meta-Analysis

#### Collection and Projection of Related Quantitative Trait Loci

Quantitative trait loci controlling rice RSA traits (based on RFLP, AFLP, SSR, and SNP genetic markers and including the RIL, F_2_, DH, and NIL populations) were collected from reviewing of 20 independent experiments that studied two sets of bi-parental populations: IR64 × Azucena population (8 articles) and Azucena (the tolerant and deep-rooting genotype) × other rice genotypes population (12 articles) under drought and normal conditions. The root traits considered were included maximum root length (MRL), deep rooting weight (DRW), root number (RN), root length (RL), root thickness (RTHK), root volume (RV), root dry weight (RDW), deep rooting ratio (DRR), root to shoot ratio (RSR), drought stress (DS), root surface area (RSAr) and root growth angle (RGA). Afterward, the chromosome number, trait type, proportion of phenotypic variance explained by the QTL (*R*^2^), logarithm of odds ratio (LOD score), QTL confidence interval (CI), QTL chromosomal position and population size were also extracted.

A high-density marker integrated genetic map (Daryani et al., 2021) was used as a reference map for Meta-QTL analysis. The primary QTLs were projected on the consensus map based on a simple scaling method using flanking markers. Using a Gaussian distribution, new confidence intervals of the primary QTLs were approximated based on their original genetic map before the QTLs projection on the consensus map. Based on the population type, the 95% CI for each QTL position was calculated according to Daryani et al. (2021).

#### Meta-Analysis and Identification of Candidate Genes Within the Meta-Quantitative Trait Loci Regions

Applying BioMercator V4.2 (Arcade et al., 2004) contained algorithms from the MetaQTL software (Sosnowski et al., 2012), meta-analysis was performed according to the QTL clusters on each chromosome following QTL projection on the consensus map for both sets of IR64 × Azucena and Azucena × other rice genotypes populations, separately).

To identify candidate genes, the flanking markers of the positional CIs belonged to the extracted Meta-QTLs were applied. As the reference genome, the genome assembly of cultivated rice (*Oryza sativa* L.) was considered (Temnykh et al., 2001)^7^ and the flanking markers were mapped. Then, the physical positions were also calculated. Lastly, the identification of candidate genes was performed using BioMart data mining tools from the Ensembl Website Gramene^8^. A total of 5787 and 3511 genes were obtained for IR64 × Azucena and Azucena × other rice genotypes populations, respectively which 1047 genes were common between them. To narrow down the candidate genes, only the 1047 common genes were considered for integration with RNAseq data.

### Integrating Transcriptome Profiling and Meta-Quantitative Trait Loci Analysis

The expression level of the 1047 selected candidate genes (see the pervious section) were extracted from the transcriptome profiling data for all samples (the genes with FPKM values less than one in more than 80% of samples were removed) and their significance was analyzed by a two-way ANOVA for each zone, separately (with a *p*-value cutoff of < 0.05) to identify genes with significant interactions between genotypes and conditions.

### Quantitative Real-Time PCR Analysis

Total RNA was extracted as described above. Quantitative real-time PCR (qRT-PCR) was performed as previously described (Taheri et al., 2011). Briefly, cDNA was synthesized from 2 μL of each RNA sample (iScript cDNA Synthesis kit, Bio-Rad). The sequence information from our RNA-seq data was utilized for primer design using the Oligo 7.0 software (National Bioscience Inc., Plymouth, MA, United States), and the primer sequences were double-checked by IDT-oligo analyzer tool^9^. The qRT-PCR with three independent biological replicates was performed using a LightCycler 96 Real-Time PCR System (Roche Life Science, Germany) and TB SYBR Premix Ex Taq II based on the manufacturers’ protocol.

The expression level of each target mRNA and the housekeeping gene (UBQ) were determined in parallel for each sample. Results were expressed as the normalized ratio of the mRNA level of each gene of interest over that of UBQ using the difference between the threshold cycle values, or ΔΔCt method. Ct values for individual target genes were calculated and the ΔCt average for the housekeeping gene (UBQ) was treated as an arbitrary constant and used to calculate ΔΔCt values for all samples. Three independent biological replicates were used for qRT-PCR.

## Results

### Phenotypic Responses of Two Contrasting Rice Genotypes to Water Stress

Following exposure to a 14-day water stress treatment, RWC of the susceptible and shallow-rooting genotype, IR64, dropped to 72%, while Azucena, the tolerant and deep-rooting genotype, maintained 80% of its RWC (Figure 1D and Supplementary Tables S1, S2). A significant reduction in shoot dry weight was evident in Azucena (24%), and to a greater extent in IR64 (34%) (Figure 1E and Supplementary Tables S1, S2). Changes in the root dry weight also showed a similar trend, with 36 and 13% reduction in IR64 and Azucena, respectively (Figure 1F and Supplementary Tables S1, S2).

In addition, Azucena had significantly longer roots compared to IR64 under both control and stress conditions (Figures 1C,E and Supplementary Tables S1, S2), and the rates of root elongation induced by water stress were significantly higher in Azucena (58%) compared to IR64 (23%) (Supplementary Table S2).

**FIGURE 2:**
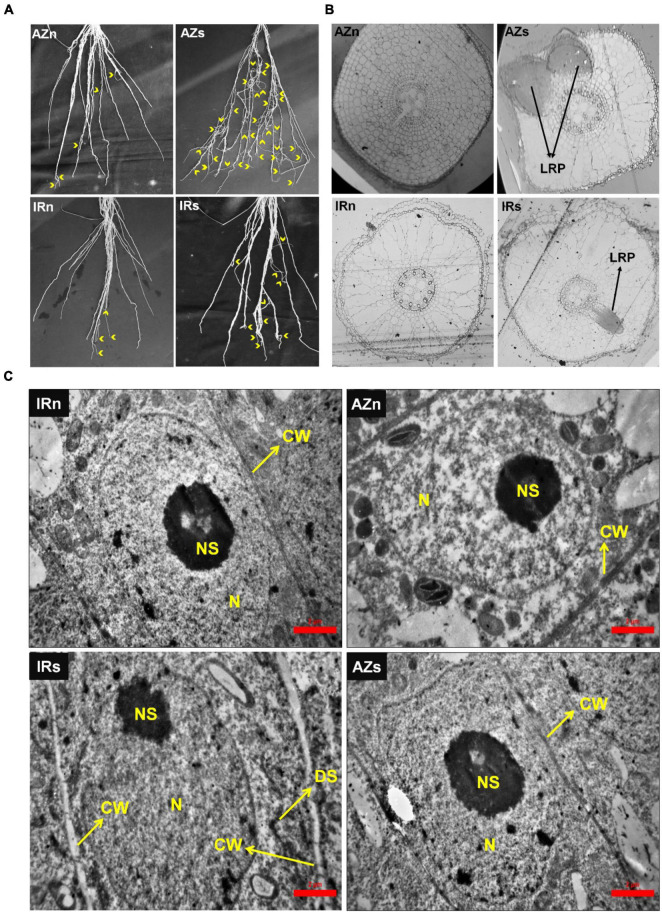
Lateral root expansion and structural and ultrastructural studies of Azucena and IR64 root tips in response to water stress. **(A)** Lateral root expansion in Azucena and IR64 under normal and stress conditions; Yellow arrows indicate lateral roots. **(B)** Tissue structural studies of root (zone3) in both genotypes under normal and stress conditions shows the number of lateral root primordia. **(C)** Ultrastructural studies of root meristematic cells in both genotypes under normal and stress conditions shows cell wall thickening in IR64 in response to stress. IRn, IR64 genotype under normal condition; IRs, IR64 genotype under water-stress condition; AZn, Azucena genotype under normal condition; AZs, Azucena genotype under water-stress condition; Z1, Root Z1; Z2, Root Z2; Z3, Root Z3; CW, cell wall; N, nucleus; NS, nucleolus; DS, dictyosome; LRP, lateral root primordium.

Lateral roots expansion, evident in both genotypes in response to stress, was more significant in Azucena (Figure 2A). The number of macroscopic lateral roots was counted along a 30-cm section (from tip) of the longest crown roots of both genotypes under both conditions (Supplementary Figure S1). The number of lateral roots were increased 4.5 fold in Azucena under water stress while in IR64 it was only 2.5 fold. Structural studies revealed different numbers of LR primordia in a 2 mm section of Z3. Our transverse serial sections showed no LR primordia in the two genotypes grown under well-watered conditions (Figure 2B). However, under water stress, LR primordia were observed in both genotypes with Azucena having a greater number. These observations may be reflective of the role of LR growth and development in assisting the tolerant genotypes to cope with water shortage.

Furthermore, cellular ultrastructural studies of meristematic root zone (Z1) revealed cell wall thickening of meristematic cells in IR64 roots, in response to water stress, with no change in those of Azucena (Figure 2C). Plant cells are surrounded by plasma membrane and cell wall, with the former appearing dark, after fixation in osmium tetroxide and staining with uranyl acetate and lead citrate, due to their lipid structure, and the latter appearing white due to its polysaccharide content (Alberts, 2018). The very thin cell wall surrounding a typical meristematic cell makes it barely detectable, as shown in Azucena under both conditions and IR64 under the normal condition. However, in IR64 samples exposed to stress, it presented in the form of a white boundary, possibly indicating an increase in the thickness of the cell wall (Figure 2C).

### Transcriptome Profiling of Root Zones Under Control and Water Stress Conditions

RNAseq profiling was performed on three consecutive root tip zones in the two contrasting genotypes of rice under two different conditions and approximately 15–22 million read pairs were obtained for each sample (A total of 452,452,035 read pairs; Supplementary Table S3 and Supplementary Figure S2). Reads were aligned to the *O. sativa* cv. Nipponbare (ssp. *japonica*) reference genome sequences IRGSP1.0^10^ using Hisat2 (Kim et al., 2015). The percentage of mapping rates and unique mapping rates for all samples were between 94–96% (with an average of 95.17%) and 84–90% (with an average of 88.57%), respectively (Supplementary Table S3 and Supplementary Figure S2). Expression levels for the transcripts were calculated by quantifying the reads according to the FPKM method (Trapnell et al., 2010).

**FIGURE 3:**
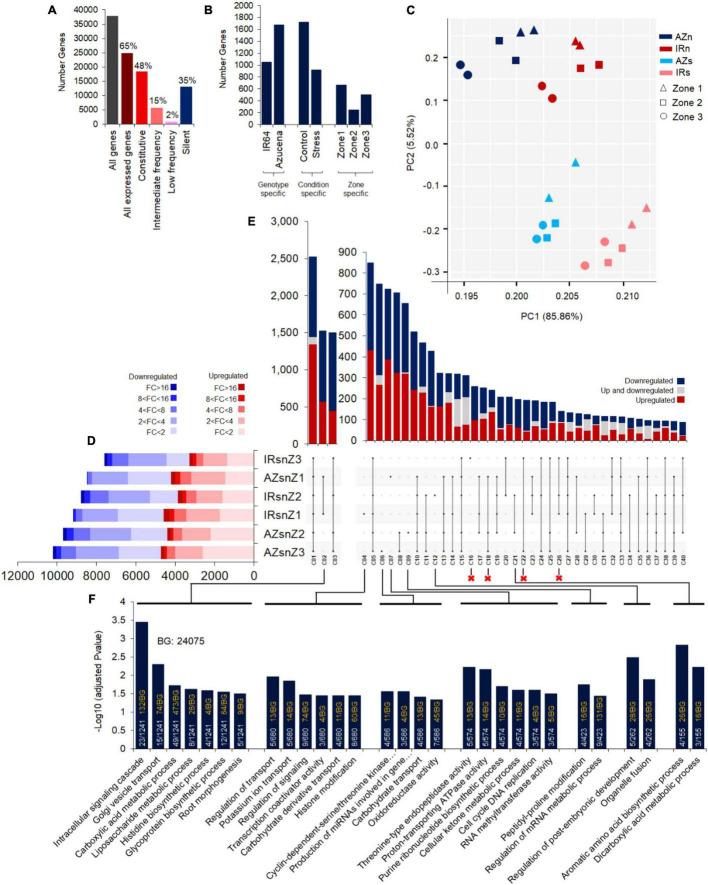
Transcriptome profiling of Azucena and IR64 root zones under control and water stress conditions and the intersections of DEGs across 6 interaction of genotypes, zones and conditions. **(A)** The number of genes not expressed in any sample (the silent group) (FPKM < 1 in none of the doublet sets of replicates), expressed in less than 20% of samples (Low frequency), expressed in 20–80% of samples (Intermediate frequency), and expressed in more than 80% of samples (Constitutive). **(B)** The number of genes expressed in only one genotype (All replicates of three zones; Genotype specific), one condition (All replicates of the condition; Condition specific), and one zone (All replicates of the zone; Zone specific). **(C)** Principal component analysis (PCA) visualization of all 24 samples in a 2D space. PCA were used on Log2 transformed FPKM values of a set of 24742 genes (about 65% of all identified annotated genes) expressed in at least one set of doublet replicates (FPKM > 1) in this analysis. The color indicates genotype and condition (IRn, IR64 under normal condition; AZn, Azucena under normal condition; IRs, IR64 under stress condition; AZs, Azucena under stress condition) and the shapes indicates root zones. **(D)** Horizontal bar graph represents the number of up- (Red boxes) and down- (Blue boxes) regulated DEGs (FDR adjusted *p*-value cut-off of ≤ 0.05) based on their fold changes. **(E)** The intersections of DEGs across the six gene lists in the vertical bar graph (40 out of 62 intersections presented and the information on the others can be found in Supplementary Table S6). Sections colored in red/blue depict genes up/down regulated synchronously, while the gray sections are the ones upregulated in one and downregulated in the other and vice versa. The circles below each bar with black color indicate which sets are in the intersection. **(F)** The significantly enriched BPs are shown as barplots in the bottom panel based on their –log10 adjusted *p*-values. The proportion of gene numbers included in each BP in annotated gene numbers of the cluster (white text) along with the proportion of total gene numbers included in each BP in annotated gene numbers of the background (yellow text) were mentioned in the bars. IRsnZ1, expression changes in zone1 of IR64 (Stress vs. Control); IRsnZ2, expression changes in zone2 of IR64 (Stress vs. Control); IRsnZ3, expression changes in zone3 of IR64 (Stress vs. Control); AZsnZ1, expression changes in zone1 of Azucena (Stress vs. Control); AZsnZ2, expression changes in zone2 of Azucena (Stress vs. Control); AZsnZ3, expression changes in zone3 of Azucena (Stress vs. Control).

A total of 37846 annotated genes were identified, 65% of which (24742 genes) were actively expressed (FPKM > 1 in at least two biological replicates of one sample), whereas, about 35% (13104 genes) were not expressed (FPKM = 0) or expressed at very low levels (1 > FPKM > 0) and thus considered as silent genes. Among all expressed genes (24742), there was evidence of expression for 18364 genes (48% of all annotated genes) in at least 80% of all samples (referred to constitutive expression), 5580 genes (15% of all annotated genes) in 20–80% of all samples (intermediate frequency), and 798 genes (2% of all annotated genes) in less than 20% of all samples (low frequency) (Figure 3A). The enriched biological processes (BPs) terms for the identified gene sets are shown in Supplementary Table S4.

There were 1053 and 1675 genes specifically expressed in IR64 and Azucena, respectively (referred to genotype specific), and 1723 and 916 genes expressed specifically in control and stress conditions, respectively (referred to condition specific). In the zone-specific category, 661, 250, and 503 genes were only expressed in Z1, Z2, and Z3, respectively (Figure 3B). These results suggest substantial differences in expression patterns in all genotypes, conditions, and the zones examined here. The enriched BPs terms for these identified gene sets are shown in Supplementary Table S5.

Principal component analysis (PCA) on the 24742 expressed genes illustrated the clustering of samples by zones, genotypes and conditions. Pearson correlation coefficients based on gene expression levels (FPKMs) between each pair of samples are shown as a heatmap, indicating the consistency between biological replicates of each sample (Supplementary Figure S3). The first two PC dimensions, accounting for 91% of the variation, was sufficient for clear separation of the genotypes and conditions (Figure 3C and Supplementary Figure S3). The PCA analysis clearly presented the distinct expression patterns between stress and normal conditions. Furthermore, Z1 grouped separately from Z2 and Z3 (Figure 3C and Supplementary Figure S3).

### Differential Expression Analysis

#### Differentially Expressed Genes Between Control and Stress Conditions

Differentially expressed genes for each genotype and zone were identified and the fold change was calculated by dividing the expression level under stress conditions by control conditions. An Upset plot was used for visualization of the number of up- (red boxes) and down- (blue boxes) regulated DEGs across different genotypes and zones (Figure 3D). The data on DEGs in Z1, Z2, and Z3 of IR64 and Azucena identified a fraction of nearly 35–45% with modest levels of change (less than twofold change), while the remainder showed greater levels of change (more than twofold change) in transcript abundance. Interestingly, about 5–10% of DEGs showed 8- to 16-fold change (Figure 3D).

The intersections of DEGs across these six groups, labeled C1-C40, indicated their dynamic patterns in different zones and genotypes, as shown in the vertical bar graph (Figure 3E). The significantly enriched BPs of eleven important intersections representing zone-, genotype-, and zone and genotype-specific responses are shown in bar plots (based on –log10 adjusted *p*-values) at the bottom panel (Figure 3F) while the remains are exhibited in the Supplementary Table S6. The results from zone and genotype-specific DEGs revealed the enrichment of different BPs by IR64 and Azucena: In Z1 of Azucena roots (C07), six BPs were specifically enriched, such as those involved in cell cycle and division and ATPase activity (Figure 3F). In IR64 (C04), on the other hand, of the six enriched BPs, four were related to signaling, and transporting (Figure 3F). The BPs specifically enriched in Z2 of Azucena (C09) included peptidyl-proline modification and regulation of mRNA metabolic processes, while, in Z2 of IR64 (C12) different BPs were enriched including the regulation of post-embryonic development and organelle fusion (Figure 3F). Genes specifically expressed in Z3 of Azucena (C06) corresponded to BPs including cyclin-dependent protein serine/threonine kinase activity, production of miRNAs involved in gene silencing, carbohydrate transport and oxidoreductase activity. In contrast, no significantly enriched BPs were detected in Z3 of IR64 (C16) (Figure 3F).

**FIGURE 4:**
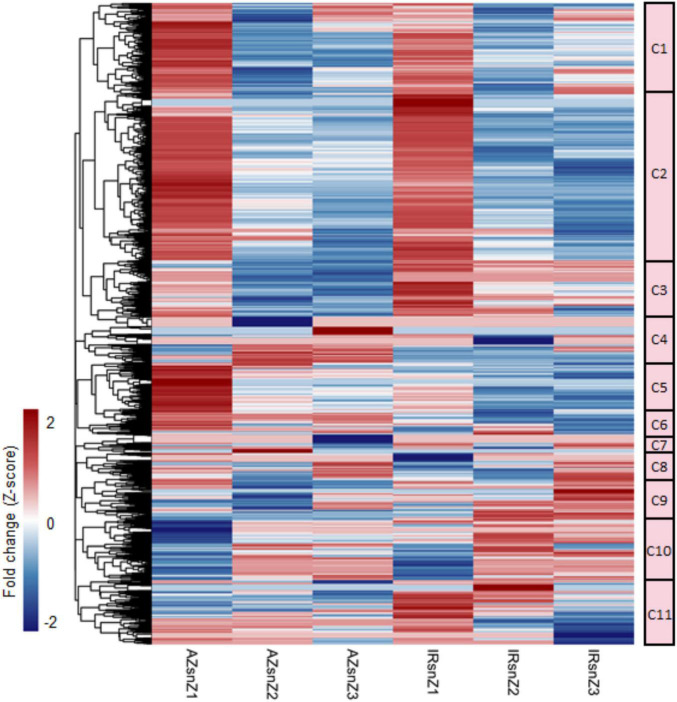
An overview of DEGs and co-expressed clusters across rice genotypes and root zones in response to water stress. Heatmap representing the hierarchical clustering of DEGs based on the fold-change values (clustering method: complete; clustering distance: euclidean). The color bar depicts the gradient of fold changes in response to water stress. The matrix was included the FC values of those genes that were DEG in at least one zone (the non-differentially expressed FC values were set to zero.). The 11 main clusters (C1–C11) were marked. Please see the enriched GO terms belonged to each cluster in Supplementary Table S7.

To present an overview of data and identify major co-expressed clusters across both genotypes and all root zones in response to water stress, a hierarchical clustering was performed on DEGs based on the fold-change values by complete method and Euclidean distance measurement (Figure 4). The matrix was included the FC values of those genes that were DEG in at least one zone (the non-differentially expressed FC values were set to zero.). The 11 main clusters (C1–C11) were considered for GO enrichment analysis. Supplementary Table S7 shows the significantly enriched GO terms belonged to the clusters 1–5 and 9–11 (C1–C5 and C9–C11) however, no enriched GO terms were obtained for the clusters 6, 7, and 8. Interestingly, the genes belonged to C5 strongly upregulated in zone one of Azucena, were enriched for generation of precursor metabolites and energy, electron transport chain, and cell cycle BPs while the C11 containing genes with upregulation patterns in zone 1 and 2 of IR64 root were enriched for response to water stress and polysaccharide catabolic process (Supplementary Table S7 and Figure 4).

### Differentially Expressed Genes With a High Fold Change Between Control and Stress Conditions

**FIGURE 5:**
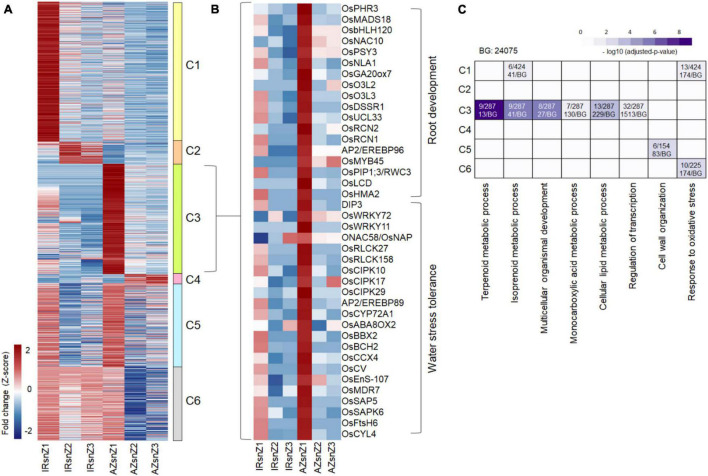
Differentially expressed genes with a high fold change between control and stress conditions in two contrasting genotypes of rice. **(A)** Heatmap representing the DEGs with high fold changes (more than fourfold) between control and stress in the six clusters with distinct expression patterns across the three root zones of two contrasting genotypes of rice. The color bar depicts the gradient of fold changes in response to water stress. **(B)** Heatmap representing a subset of 40 genes extracted from cluster 3 with roles in root development and water stress tolerance. **(C)** BPs enriched in clusters 1–6 (C1–C6). The color bar depicts the gradient of –log10 (adjusted *P*-value) where *P*-value is the significance of individual GO-BPs enriched with each cluster. The proportion of gene numbers included in each BP in annotated gene numbers of the cluster (on top) along with the proportion of total gene numbers included in each BP in annotated gene numbers of the background (at bottom) were mentioned in the boxes. IRsnZ1, expression changes in zone1 of IR64 (Stress vs. Control); IRsnZ2, expression changes in zone2 of IR64 (Stress vs. Control); IRsnZ3, expression changes in zone3 of IR64 (Stress vs. Control); AZsnZ1, expression changes in zone1 of Azucena (Stress vs. Control); AZsnZ2, expression changes in zone2 of Azucena (Stress vs. Control); AZsnZ3, expression changes in zone3 of Azucena (Stress vs. Control).

To analyze highly up- and down-regulated DEGs, transcripts with more than fourfold change between control and stress conditions were filtered. The filter was applied to all DEG groups belonging to all three root zones of the two genotypes, and the values of each list in the other zones and other genotype were determined. To find the genotype-specific responsive genes, only those genes with more than fourfold change differences between genotypes were kept. The collective list (1440 genes) was clustered based on fold changes in different zones and genotypes by average linkage method and Euclidean distance measurement (Figure 5A). We observed six distinct clusters corresponding to up- or down-regulated genes in response to stress. Cluster 3 consisted of genes highly upregulated in Z1 of Azucena, and included a total of 375 genes, 194 of which had functional annotation according to rice databases. Interestingly, a total of 40 genes, listed in Figure 5B, are involved in root development (18 genes) and drought tolerance (22 genes). These were included genes with important roles in rice RSA, such as *OsbHLH120* (root thickness), *OsNAC10* (root thickness and drought tolerance), *OsPHR3* (LR development), PIP1;3/RWC3 (root length and water stress avoidance), *OsMADS18* (root elongation), and *OsNLA1* (root length and growth), as well as those with key roles in enhancing drought tolerance such as *DIP3*, *CIPK10*, *CIPK17*, *CIPK29*, *OsERF101*, *OsWRKY11*, and *ONAC58*/*OsNAP*.

Gene ontology enrichment analysis was performed on all six clusters in Figure 5A, and the significant BPs (*p*-value < 0.05) are presented in Figure 5C based on their –log10 (*p*-value). Interestingly, cluster 3 (C3) containing highly up-regulated genes in zone 1 of Azucena, was specifically enriched for transcripts involved in regulation of transcription, and terpenoid, lipid and monocarboxylic acid metabolic processes, while cluster 1 (C1) containing highly up-regulated genes in zone 1 of IR64, was specifically enriched for response to oxidative stress and isoprenoid metabolic process. Response to oxidative stress was also enriched for cluster 6 (C6), which containing a gene set with upregulation patterns in all three zones of IR64 but only in zone 1 of Azucena (Figure 5C).

### Differentially Expressed Genes Involved in Root Elongation, Lateral Roots Expansion and Meristematic Cell Wall Thickening

The main goal of this study was identification of genes involved in rice RSA traits. To find more associated candidate genes, an expand literature review was performed to prepare a list of related genes reported previously in the research articles and the rice databases. According to the results of our phenotypic observations indicating induced root elongation, LR expansion and meristematic cell wall thickening in response to stress, three lists of 230, 154, and 581 genes affecting root length, LR development and cell wall formation and modification, respectively, were considered for surveying of their expression patterns using our dataset (Supplementary Table S8). Of these, a total of 186, 113, and 403 genes with FPKM values of more than one in at least 20% of the samples, respectively, were selected for further analysis. The two-way ANOVA led to the identification of interesting DEGs with significant interactions for genotypes and conditions.

**FIGURE 6:**
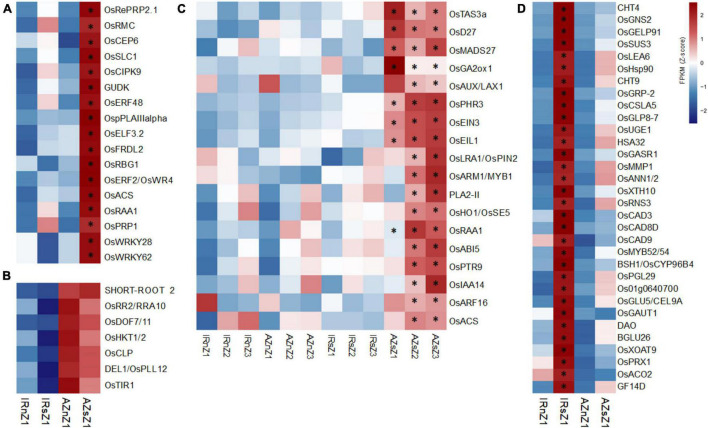
The expression pattern of DEGs involved in root elongation, LR expansion and meristematic cell wall thickening across two contrasting genotypes under water stress. **(A)** Heatmap showing the expression patterns of genes affecting root length and significantly upregulated in Z1 of Azucena roots under stress conditions. The asterisks indicate significant interactions between genotypes and conditions (*p*-value cut-off ≤ 0.05) analyzed by a two-way ANOVA test. **(B)** Genes affecting root length were expressed at much higher levels (*p*-value cut-off of ≤0.05, analyzed by *t*-tests between genotypes in both conditions) in Z1 of Azucena roots under both conditions compared to IR64. **(C)** Heatmap showing the expression pattern of genes involved in lateral root formation, growth and development, induced and upregulated in response to stress only in Azucena root zones. The asterisks indicate significant interactions between genotypes and conditions (*p*-value cut-off ≤ 0.05) analyzed by two-way ANOVA test. **(D)** Heatmap showing the expression pattern of genes involved in cell wall biogenesis and modification induced and upregulated in Z1 of IR64 roots under stress. The asterisks indicate significant interactions between genotypes and conditions (*p*-value cut-off ≤ 0.05) analyzed by two-way ANOVA test. IRnZ1, root Z1 of IR64 under normal condition; IRnZ2, root Z2 of IR64 under normal condition; IRnZ3, root Z3 of IR64 under normal condition; IRsZ1, root Z1 of IR64 under stress condition; IRsZ2, root Z2 of IR64 under stress condition; IRsZ3, root Z3 of IR64 under stress condition; AZnZ1, root Z1 of Azucena under normal condition; AZnZ2, root Z2 of Azucena under normal condition; AZnZ3, root Z3 of Azucena under normal condition; AZsZ1, root Z1 of Azucena under stress condition; AZsZ2, root Z2 of Azucena under stress condition; AZsZ3, root Z3 of Azucena under stress condition.

Our phenotypic analysis revealed Azucena roots were longer than those of IR64 under both well-watered and water-deficit conditions (Figures 1C,G). In addition, stress-induced increase in root elongation rate was about 2.5 fold higher in Azucena than in IR64 (Supplementary Table S2). Out of 186, 34 DEGs with significant interactions for genotypes and conditions were detected in Z1, the region including meristematic and elongation regions responsible for cell division and cell expansion, the two major determinants in root growth and elongation (Krizek, 2009) (Supplementary Figure S4A). Among them, 17 DEGs were upregulated in Azucena (Figure 6A), including *OsRBG1*, *OsRMC*, and *OsERF2*/*OsWR4*.

Since Azucena had longer roots than IR64 under both conditions, to identify DEGs between the two genotypes, irrespective of the treatment, a t-test analysis was performed on the genes with insignificant differences according to the ANOVA analysis. Eleven genes (Supplementary Figure S4B) showed significant differences (*p*-value < 0.05) between genotypes under both normal and stress conditions, of which seven were over-expressed in the Azucena genotype, including *OsDEL1*/*OsPLL12* and *OsDOF7*/*11* (Figure 6B).

Also, we detected LR expansion in both genotypes in response to water stress, though to a greater level in Azucena (Figures 2A,B). The analysis revealed 48 (out of 113) DEGs (Supplementary Figure S4C) significantly induced (significant at least in one zone) of which 18 were upregulated in the Azucena roots (Figure 6C), included *OsACS*, *OsARF16*, *OsPTR9*, *OsMADS27*, *OsHO1*/*OsSE5*, *OsRAA1*, and *OsAUX*/*LAX1* (Figure 6C). It is interesting to note that all 18 DEGs were significantly induced in response to stress in both zones 2 and 3, enriched for lateral root growth and development (Figure 6C).

Meristematic cell wall thickening was observed in IR64 in response to stress (Figure 2C). Out of a total of 58 DEGs with significant interaction between genotype and condition in Z1 (Supplementary Figure S4D), 32 genes were upregulated in IR64 (Figure 6D) including three members of the *CAD* gene family (*OsCAD3*, *OsCAD8D*, and *OsCAD9*) along with *OsMYB52*/*54*, *OsSUS3*, *OsPRX1*, and *BSH1*/*OsCYP96B4*.

### Gene Family Studies Across Contrasting Genotypes

To maximize the benefit of transcriptome analysis, the expression pattern of 24 important gene families with roles in RSA growth and development and drought stress response were further analyzed including *NAC*, *AP2*/*ERF*, *AUX*/*IAA*, *EXPANSIN*, *WRKY*, *MYB*, *MYB-related*, *ARF*, *EXPANSIN*, *GRAS*, *LEA*, *HSP*, *HSF*, *ARID*, *bHLH*, *bZIP*, *LOB*, *WOX*, *EIL*, *GRF*, *G2-like*, *TIP* and *PIP*, *SWEET*, *RR*, and *MADS* (Hu et al., 2009; Dal Santo et al., 2013; Janiak et al., 2016; Yoon et al., 2020). A complete list of the members, collected by searching through four databases, along with their IDs, names, and descriptions is presented in the Supplementary Table S9. By performing a two-way ANOVA for each zone, separately, we searched for genes with significant interactions for genotypes and conditions (at least in one zone). The expression patterns of DEG members (*p*-value < 05) in two genotypes in response to water stress are shown in Supplementary Figures S5–S7. It should be emphasized that the majority of the 24 families showed dynamic expression patterns, however, we have focused on six important families, namely *NAC*, *AP2*/*ERF*, *AUX*/*IAA*, *EXPANSIN*, *WRKY*, and *MYB*, while the information on the remaining families is shown in Supplementary Figures S6, S7. These six families are involved in a wide range of BPs such as plant life cycle, root growth and development and water deficit tolerance.

Out of the 121, 164, 31, 103, 120, and 56, members detected of *NAC*, *AP2*/*ERF*, *AUX*/*IAA, WRKY, MYB TF*, and *EXPANSIN* gene families, respectively, 69, 103, 27, 70, 81, and 39 had FPKM values of more than one at least in 20% of the samples considered for two-way ANOVA.

**FIGURE 7:**
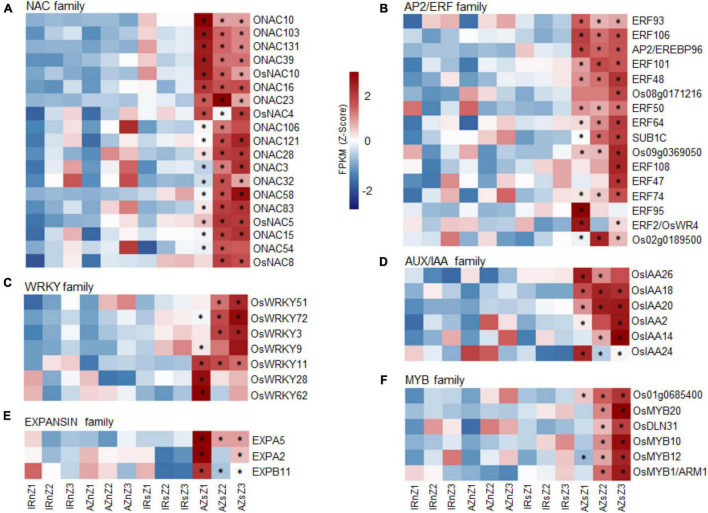
Gene family studies across contrasting genotypes in response to water stress. Heatmaps showing the expression pattern of members of **(A)** *NAC*, **(B)** *AP2/ERF*, **(C)** *WRKY*, **(D)** *AUX/IAA*, **(E)** *EXPANSIN*, **(F)** *MYB* gene families, upregulated in Azucena roots in response to water stress compared to IR64 genotype. The color bar depicts the gradient of FPKM mean (*Z*-score) for each sample. The asterisks indicate genes with significant interactions between genotypes and conditions (*p* ≤ 0.05) by two-way ANOVA test. IRnZ1, root zone 1 of IR64 under normal condition; IRnZ2, root zone 2 of IR64 under normal condition; IRnZ3, root zone 3 of IR64 under normal condition; IRsZ1, root zone 1 of IR64 under stress condition; IRsZ2, root zone 2 of IR64 under stress condition; IRsZ3, root zone 3 of IR64 under stress condition; AZnZ1, root zone 1 of Azucena under normal condition; AZnZ2, root zone 2 of Azucena under normal condition; AZnZ3, root zone 3 of Azucena under normal condition; AZsZ1, root zone 1 of Azucena under stress condition; AZsZ2, root zone 2 of Azucena under stress condition; AZsZ3, root zone 3 of Azucena under stress condition.

Of the 33 DEGs identified of the *NAC* family (Supplementary Figure S5B), 19 showed significant upregulation in Azucena root in response to water stress while they did not change significantly in IR64 (Figure 7A) and included those involved in RSA development [*OsNAC10*, *OsNAC5*, *ONAC54* (*RIM1*), *ONAC58* (*OsNAP*), and *ONAC3* (SNAC3)]. Of the 45 DEGs detected of the *AP2*/*ERF* gene family (Supplementary Figure S5A), 16 were upregulated in Azucena in response to water stress, with no significant changes in IR64 (Figure 7B). The roles of six of them in water-stress tolerance and rice root development have previously been reported; *OsERF50* (*OsDREB6*), *OsERF48*, *OsERF101*, *OsERF95* (*OsSta2*), *OsERF93*/*ERF1*, and *OsERF2*/*OsWR*. Fourteen members of *AUX*/*IAA* gene family showed differential expression under water stress at least in one zone (Supplementary Figure S5E). Of these, a total of 6 indicated significant upregulation under stress only in the Azucena genotype including *OsIAA26*, *OsIAA2*, *OsIAA18*, *OsIAA20*, *OsIAA14*, and *OsIAA24* (Figure 7D). Also, 22 DEGs were detected of *WRKY* gene family (Supplementary Figure S5C) in which seven DEGs were upregulated in Azucena under water stress with no significant changes in IR64. These include *OsWRKY51*, *OsWRKY72*, *OsWRKY3*, *OsWRKY9*, *OsWRKY11*, *OsWRKY28*, and *OsWRKY62* (Figure 7C). The analysis led to the identification of 33 DEGs of the *MYB TF* family (Supplementary Figure S5F). Six genes (*OsMYB12*, *OsMYB20*, *OsDLN31*, *OsMYB10*, *OsMYB1*/*ARM1*, and *Os01g0685400*) showed significant upregulation in Azucena roots in response to water stress while they did not change significantly in IR64 (Figure 7F). Thirteen DEGs were detected of the *EXPANSIN* gene family (Supplementary Figure S5D). Despite the ten DEGs downregulated in both genotypes the expression level of 3 members (*EXPA2*, *EXPB11*, and *EXPA5*) only increased only in Azucena roots, especially in Z1 in response to water stress with no significant changes in IR64 (Figure 7E).

In addition, our results revealed that the members of two *HSP* (heat shock protein) and *HSF* (heat shock factor) gene families were strongly upregulated in IR64 root in response to water stress, compared to Azucena. Out of 25 and 39 members detected for *HSF* and *HSP* gene families, respectively, 19 and 24 members showed significant interactions for genotypes and conditions at least in one zone. Of these 14 *HSF* genes (including *OsHSFA2a*, *OsHSF-16*, *OsHSFB2b*, *OsHSFC1b*, *OsHSFA2c*, *OsHSFA3*, *OsHSFB2a*, *OsHSFB4a*, *OsHSFB2c*, *OsHSFA7*, *OsHSFA2d*, *OsHSFA9*, *OsHSF15*, and *OsHSFA2b*) and 19 *HSP* genes (including *OsHSP70-4*, *OsHSP18.6*, *OsHSP70CP1*, *OsHSP101*, *OsHSP18.0-CIII*, *OsHSP90.1*, *OsHSP17.9B*, *OsHSP26*, *OsHSP74.8*, *OsctHSP70-1*, *OsHSP17.3*, *OsHSP17.7*, *OsHSP70CP2*, *OsHSP90-1*, *OsHSP16.9C*, *OsHSP17.9A*, *OsHSP24.1*, *OsHSP16.9A*, and *OsHSBP2*) were significantly upregulated in IR64, the sensitive and lowland genotype, while they did not change significantly in Azucena (Supplementary Figures S7A,B). These results may suggest the important roles of *HSP* and *HSF* families in IR64 to cope with water-deficit stress.

Collectively, several RSA associated genes were identified as drought responsive genes in both gene family and root elongation, LR expansion and meristematic cell wall thickening studies (from sections “Differentially Expressed Genes Involved in Root Elongation, Lateral Roots Expansion and Meristematic Cell Wall Thickening” and “Gene Family Studies Across Contrasting Genotypes”) which their roles in response to drought have not been reported yet. For example, we found two subsets of 17 (*OsPHR3*, *OsMADS18*, *OsACS*, *OsARF16*, *OsHO1*, *OsPTR9*, *OsRAA1*, *OsRMC*, *OsDOF11*, *OsERF2*, *OsDEL1*, *ONAC52*/*RIM1*, *OsEXPA2*, *OsIAA20*, *OsIAA26*, and *OsWRKY72*) and 7 (*OsCAD3*, *OsCAD8D*, *OsCAD9*, *OsMYB52*/*54*, *OsSUS3*, *OsPRX1*, and *BSH1*/*OsCYP96B4*) genes with positive and negative roles in RSA formation and modification, respectively. Considering their induction in response to water deficit stress, their potential roles in adaptation of rice to water stress needs to be further investigated.

### Integrating Transcriptome Profiling and Meta-Quantitative Trait Loci Analysis

**FIGURE 8:**
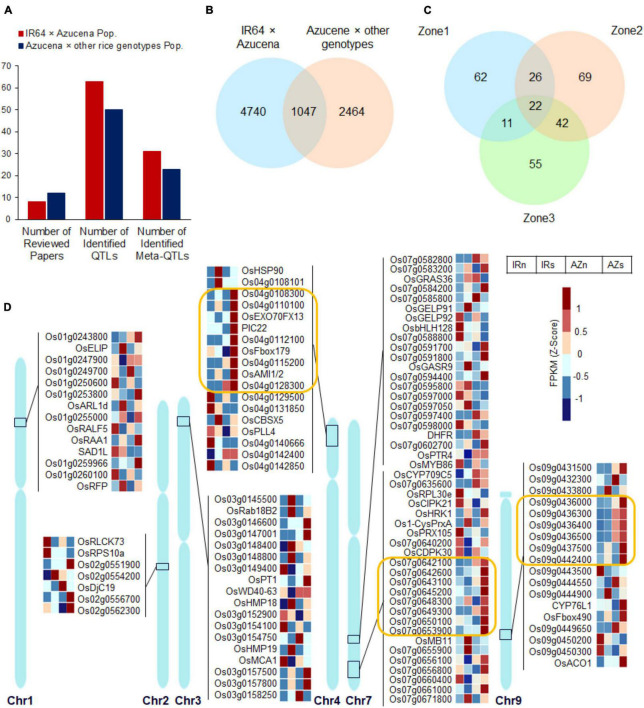
The expression patterns of the genes extracted from integrating transcriptome profiling and Meta-QTL analysis which had significant interactions between genotypes and conditions. **(A)** Barplot showing the number of reviewed articles, collected QTLs, and the identified Meta-QTLs across the populations. **(B)** Venn diagram for extracted genes belonged to Meta-QTLs identified across the populations and their overlaps. **(C)** Venn diagram showing the number of genes with significant interactions (with a *p*-value cutoff of <0.05) between genotypes and conditions (a two-way ANOVA was performed on 1047 genes detected from the overlapping of the populations) in three zones and their overlaps. **(D)** The expression pattern of the DEGs belonged to Z1, on their chromosomal regions; three subsets of genes on chromosomes 4, 7, and 9 showing co-upregulation in response to water stress in Azucena were surrounded by orange lines. IRnZ1, root Z1 of IR64 under normal condition; IRsZ1, root Z1 of IR64 under stress condition; AZnZ1, root Z1 of Azucena under normal condition; AZsZ1, root Z1 of Azucena under stress condition.

Many QTLs related to rice RSA traits were identified by linkage analysis from different populations across different water conditions, so far, which are valuable resources for integrating with transcriptome profiling and finding novel candidate genes. In this study, 20 independent experiments (Supplementary Table S10 and Figure 8A) based on bi-parental populations were reviewed included two sets of IR64 × Azucena and Azucena × other rice genotypes populations (with emphasis on Azucena, the tolerant and deep-rooting genotype). A total of 132 QTLs controlling RSA traits under drought and normal conditions (including 82 and 50 from the two mentioned population sets, respectively) were collected (Figure 8A and Supplementary Table S11). The RSA traits included were MRL, DRW, RN, RL, RTHK, RV, RDW, DRR, RSR, DS, RSAr, and RGA. Meta-QTL analysis were performed on both sets of collected QTLs separately. It led to identification of 31 and 23 significant Meta-QTLs with confidence interval (CI) of 0.11–17.67 cM and 0.055–5.2 cM which were 2.52 and 3.07 times narrower than the mean CI of the original QTLs for IR64 × Azucena and Azucena × other rice genotypes populations, respectively. They were located on all 12 rice chromosomes (Figure 8A and Supplementary Table S12) and were included a total of 5787 and 3511 genes (Figure 8B and Supplementary Table S13). Among them, 1047 common genes were detected located on chromosomes 1, 2, 3, 4, 7, and 9. To narrow down the candidate genes, only the 1047 common genes (Figure 8B), were considered for integrating with RNAseq data. The locations of these genes along with the information about the associated Meta-QTLs are shown in Supplementary Figure S8. A two-way ANOVA analysis of the expression levels in 1047 common genes across the two genotypes and two conditions showed that the expression level of 121, 159, and 130 genes changed significantly (*p* < 0.05) in zones 1, 2, and 3, respectively (Figure 8C and Supplementary Table S14). Of these, some were common between all three zones while others were zone-specific. Subsets of 22, 48, 64, and 33 genes overlapped between all zones, zones 1 and 2, zones 2 and 3, and zones 1 and 3, respectively (Figure 8C). The expression patterns of the DEGs in Z1 (the region enriched for root traits such as length, thickness and angle) are shown in Figure 8D, while those in Z2 and Z3 are shown in Supplementary Figure S9. In zone one 14, 7, 18, 18, 46, and 18 genes were differentially expressed in chromosomes 1, 2, 3, 4, 7, and 9, respectively (Figure 8D and Supplementary Figure S8). These genes, in particular those induced in the tolerant and deep-rooting genotype Azucena, may represent novel candidate genes potentially involved in RSA modification and response to water stress conditions. Some of them had functional descriptions, while others lacked. For example, in obtained Meta-QTLs on chromosome 9 (which control DRR trait), three annotated genes, *CYP76L1* (Cytochrome P450 76L1), *OsFbox490* and *ACO1*, along with a set of six unannotated genes adjacent to each other (surrounded by orange lines) were detected which were co-upregulated significantly in Azucena (Figure 8D).

**FIGURE 9:**
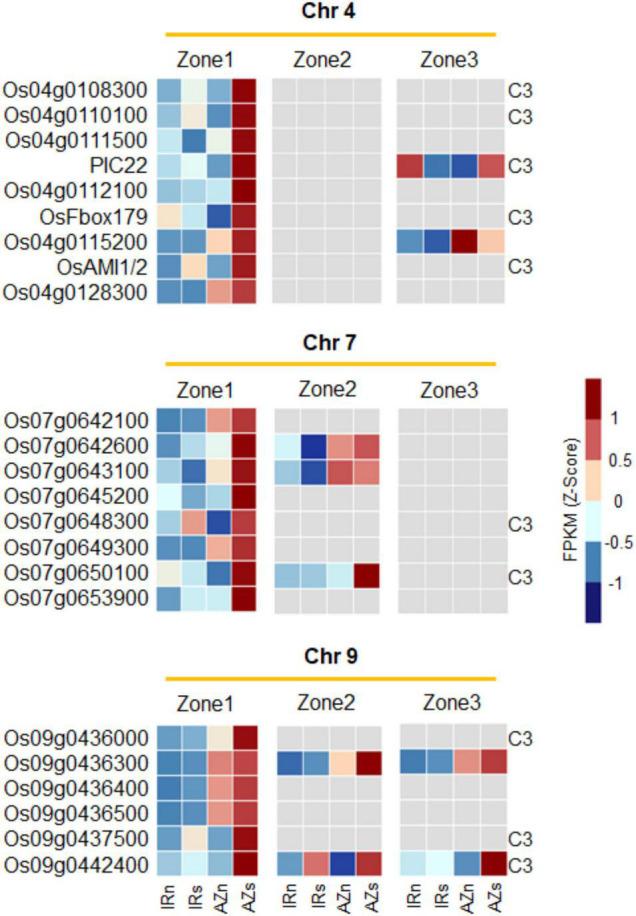
The expression pattern of the co-expressed regions on chromosome 4, 7, and 9 across all three root zones revealing they are zone 1-specific. Three subsets of genes on chromosomes 4, 7 and 9 co-upregulated in zone 1 of Azucena in response to water stress, and their expression pattern in Zones 2 and 3. Gray color depicts no significant change under stress. The genes co-localized with cluster 3 in the Figure 4A (highly upregulated DEGs in Z1 of Azucena) were labeled as C3. IRn, IR64 under normal condition; IRs, IR64 under stress condition; AZn, Azucena under normal condition; AZs, Azucena under stress condition.

As shown in Figure 8D, two another co-expressed regions (surrounded by orange lines) on chromosomes 4 and 7 were observed upregulated significantly in Z1 of Azucena. They may be considered as important regions including key candidate genes involved in RSA traits and tolerance to water stress. Due to the importance of these regions, their expression patterns were also examined in zones 2 and 3. The majority of which were found to be insignificant (Figure 9). Only 2, 3, and 2 genes on chromosomes 4, 7, and 9, respectively, showed significant differential expression in zones 2 and 3, while the remainder were zone 1-specific, the zone enriched for deep-rooting traits (Figure 9). Collectively, these results may be indicative of the potential roles of these genes in determining RSA traits.

The co-expressed region on chromosome 4 included a total of nine genes: *Os04g0108300*, *Os04g0110100* [GO-Molecular function (MF): carbohydrate binding], *Os04g0111500* (GO-BP: exocytosis and protein transport), *PIC22* (resistance gene analog *PIC22*/*Os04g0111900*; GO-BP: defense response; GO-MF: ADP binding; NB-ARC domain), *Os04g0112100* (GO-MF: ADP binding; NB-ARC domain), *OsFbox179* (*F-box protein 179*/*Os04g0113000*), *Os04g0115200*, *OsAMI1*/*2* (*Amidase2*/*Os04g0117900*; GO-BP: response to abscisic acid, IAA biosynthesis; GO-MF: amidase activity), and *Os04g0128300*. These genes were extracted by overlaying Meta-QTL 4.1 (IR64 × Azucena population) and Meta-QTL 4.1 (Azucena × other rice genotypes population) which control the RDW and RN traits (Figure 9 and Supplementary Figure S8).

The region on chromosome 7 contains the following 8 genes: *Os07g0642100* (GO-BP: DNA repair; GO-MF: DNA binding), *Os07g0642600*, *Os07g0643100* (putative esterase; GO-MF: hydrolase activity), *Os07g0645200*, *Os07g0648300*, *Os07g0649300*, *Os07g0650100* (transmembrane helix, integral component of membrane), and *Os07g0653900*. They were identified by the overlap between two Meta-QTLs (Meta-QTL 7.4 and 7.4) controlling the RSR and DRW traits (Figure 9 and Supplementary Figure S8).

Moreover, six genes were included in the region on chromosome 9, namely *Os09g0436000*, *Os09g0436300*, *Os09g0436400*, *Os09g0436500* (GO-BP: branched-chain amino acid biosynthesis, isoleucine and valine biosynthesis; GO-MF: oxidoreductase activity, ketol-acid reductoisomerase activity), *Os09g0437500*, and *Os09g0442400*. This region was obtained by overlaying of two Meta-QTLs which control DRR traits (Meta-QTL 9.3 and 9) (Figure 9 and Supplementary Figure S8).

Considering the lack of functional annotation for the majority of these transcripts, they are reported here as novel candidate genes potentially involved in RSA modification and response to water stress conditions.

### Validation by qRT-PCR

**FIGURE 10:**
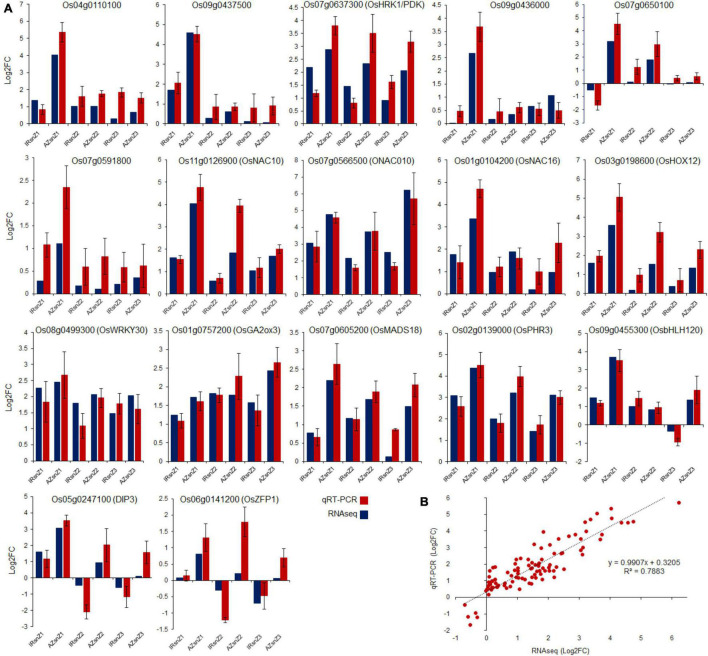
Validation by qRT-PCR. **(A)** Validations of selected genes using qRT-PCR in all three root zones of both genotypes, Azucena and IR64, in response to water stress. Data points are represented as log2fold change values. **(B)** Linear regression analysis between qRT-PCR and RNA-seq results (*R*^2^ = 0.7883) for 17 genes. *X*-axis numbers represent the log2fold-change values of mRNA-seq results. *Y*-axis numbers represent the log2fold-change values of qRT-PCR results.

Technical and biological variations in the data were checked by performing qRT-PCR of 3 independent biological replicates on 17 DEGs. These include six genes from integrating Meta-QTLs and RNAseq data (*Os04g0110100*, *Os07g0650100*, *Os07g0637300*/*OsHRK1*/*PDK*, *Os09g0437500*, *Os07g0591800*, and *Os09g0436000*), three genes from *NAC* gene family (*Os11g0126900*/*OsNAC10*, *Os07g0566500*/*ONAC010*, and *Os01g0104200*/*OsNAC16*), five genes with a high-fold change in expression levels (*Os07g0605200*/*OsMAD18*, *Os02g0139000* /*OsPHR3*, *Os09g0455300*/*OsbHLH120*, *Os05g0247100*/*DIP3*, and *Os11g0126900*/*OsNAC10*), and four randomly selected DEGs (*Os01g0757200*/*OsGA2ox3*, *Os03g0198600*/*OsHOX12*, *Os08g0499300*/*OsWRKY30*, *Os06g0141200*/*OsZFP1*). The qRT-PCR results were highly consistent with those of RNA sequencing in all three root zones in both genotypes in response to water-deficit stress (Figure 10A). The small variations in Log2FC values are probably due to a combinatorial effect of biological variation and the different mathematical models used to calculate expression levels from either qRT-PCR or RNA-Seq data (Kyndt et al., 2012). Scatter plots showed simple linear regression, and the *R*-squared (*r*^2^) between relative expression, based on log2FC obtained by FPKM values of RNAseq data (X), and the analyzed values from qRT-PCR (Y) (Figure 10B). The coefficient of variation was 0.79 for all samples.

## Discussion

In this study we investigated the transcriptomic response to water stress of three parts of root tips in two contrasting rice genotypes; Azucena, with a deep-rooting system, and IR64, with a shallow-rooting system. The aim of this was to develop a better understanding of RSA, an important developmental and agronomic trait with vital roles in plant adaptation and productivity under water-limited environments.

As expected, Azucena maintained a higher level of leaf RWC, less reduction in root and shoot dry weight, and a higher growth rate of roots in response to water stress, when compared to IR64. The transcriptomics analysis of the three consecutive root tip zones identified thousands of genes differentially expressed in the two genotypes under normal and stress conditions. A major challenge of big data is how to analyze and translate it into new biological knowledge, and generate a short list of the most important differentially expressed genes. Here we discuss several approaches we utilized to maximize the benefit of transcriptome analysis of root tips in our contrasting rice genotypes.

### Differentially Expressed Genes Between Control and Stress Conditions: Differentially Expressed Genes in Z1 of Azucena Root Are Specifically Enriched for Root Growth Maintenance Biological Processes

The results revealed different BPs which were enriched in zones 1, 2, and 3 of Azucena and IR64. Of great interest was Z1 of Azucena, as it was the only group enriched for genes involved in cell cycle and division and ATPase activities and energy generation. This may suggest intrinsic differences between Azucena and IR64, used to maintain root growth under water stress condition. Cell cycle regulation which plays key roles in maintaining plant growth at times of stress must be balanced with adoption to dynamic environmental conditions (Qi and Zhang, 2020). The ATPases extrude protons from cells to generate an electrochemical proton gradient which has a major role in providing the energy for physiological functions such as cell growth (Falhof et al., 2016). Activation of the ATPases is important in auxin-mediated cell elongation during wheat embryo development (Rober-Kleber et al., 2003). In Arabidopsis, auxin induces hypocotyl elongation through phosphorylation and activation of the ATPases (Takahashi et al., 2012). Also, ATPase activities are responsible for water maintenance, osmotic regulation and other adaptive mechanisms under drought stress (Ober and Sharp, 2003; Liu et al., 2005).

### Highly Upregulated Differentially Expressed Genes in Z1 of Azucena Are Enriched for Genes Involved in Root System Architecture Development While in IR64, Response to Oxidative Stress Is Enriched

Interestingly, about 2000 DEGs showed more than a fourfold change in response to stress across different genotypes and zones, and these clustered in 6 expression groups, labeled C1 to C6. Among them, C3 included 375 genes highly upregulated, specifically in Z1 of Azucena. Our GO enrichment analysis revealed that the C3 group was specifically enriched for transcripts involved in the regulation of transcription processes, as well as terpenoid, lipid and monocarboxylic acid metabolic processes. Terpenoids and their derivatives play essential roles in plant growth and development, and represent specialized metabolites mediating environmental adaptation (Tholl, 2015). Their functions in water stress response have also been reported (Vaughan et al., 2015; Savoi et al., 2016). Lipids are important components of cell membranes, and a change in their composition may help maintain membrane integrity and preserve cell compartmentation under water stress conditions (Gigon et al., 2004). Drought-tolerant plants have reaction mechanisms to maintain cellular homeostasis by lipid metabolism, and can regulate metabolic homeostasis (De Paula et al., 1990).

The C3 cluster was also enriched for genes involved in RSA development and water stress tolerance. These included the five key genes *OsNAC10*, *OsbHLH120*, *OsPHR3*, *PIP1;3*/*RWC3*, *OsMADS18*, and *OsNLA1*. Rice root-specific overexpression of *OsNAC10* increases root thickness and improves drought tolerance and grain yield under field drought conditions (Jeong et al., 2010). *OsbHLH120*, is known to control root thickness and length in upland rice genotypes, and is strongly induced by drought and salt stress (Li et al., 2015). *OsPHR3* is involved in root hair formation. Loss-of-function mutations in *OsPHR3* impair root hair growth and elongation (Guo et al., 2015). Overexpression of *PIP1;3*/*RWC3* in lowland rice exhibited higher levels of drought avoidance (Lian et al., 2004) and in Arabidopsis showed longer roots under stress conditions (Mosa et al., 2016). *OsMADS18*, strongly expressed in root meristems, causes a reduction in the number and elongation rate of adventitious root primordia when mutated (Fornara et al., 2004).

The C3 was also included several key genes involved in drought adaptation such as *DIP3*, *CIPK10*, *CIPK17*, *CIPK29*, *OsERF101*, *OsWRKY11*, and *ONAC58*/*OsNAP*. *DIP3*, annotated as a stress-induced protein, codes for a chitinase III, and is involved in the regulation of drought stress response in upland rice (Guo et al., 2013). *OsCIPK17* seems to participate in cross-talk among drought, salt stress and ABA treatment signaling pathways (Xiang et al., 2007). Rice plants overexpressing *OsERF101*, an important positive regulator in the adaptive response to water stress, are relatively more tolerant toward water stress (Jin et al., 2018). Overexpression of *OsWRKY11* confers heat and drought tolerance in rice seedlings (Wu et al., 2009), and its ectopic expression induces constitutive expression of drought-responsive genes in rice (Lee et al., 2018). Finally, the upregulation of *ONAC58*/*OsNAP* in rice in response to ABA and abiotic stresses, including high salinity, drought and low temperature, have been reported, and plants overexpressing *ONAC58*/*OsNAP* show enhanced tolerance and improved yield under drought stress (Chen et al., 2014).

In addition, our result showed that the cluster 1 (C1), containing genes highly upregulated in zone 1 of IR64, was enriched for genes involved in oxidative stress response. Response to oxidative stress was also enriched for cluster 6 (C6) which containing a gene set with upregulation patterns in all three zones of IR64. An important consequence of water stress is the closure of stomata which leads limits CO2 fixation and causes excessive production of reactive oxygen species (Das and Roychoudhury, 2014). When plants are exposed to water limitation and dehydration, oxidative or secondary stress occurs, thereby plants have to adjust their signaling pathways and metabolisms to ensure their growth and development (Scarpeci et al., 2008). It appears that IR64 is incapable of avoiding long-lasting water stress (Uga et al., 2013), shows an oxidative-stressed status while the upland Azucena genotype is in a water deficit avoidance stage. The enriched pathways of growth maintenance along with the increase in root length, observed in Azucena, is in accordance with the strategy of plants coping with water stress through root exploration to access water in deeper soil layers (Gilbert and Medina, 2016). IR64 probably resists water stress by limiting the growth rate and starting antioxidant system to deal with excess reactive oxygen species. It has been reported that, under severe stress, the oxidative stress markers and enzymes revealed a significant and strong upregulation by drought in IR64 (Melandri et al., 2021). However, there are studies that claimed that the inefficient antioxidant defense system in IR64 made it susceptible to water stress (Nahar et al., 2018; Sevanthi et al., 2021).

To cope with environmental stresses, plants have developed different adaptive strategies in a genotype-dependent manner (Rosa et al., 2009). Differential adaptation strategies to water stress in upland and lowland genotypes of rice have been previously reported (Xia et al., 2019; Abdirad et al., 2020; Luo et al., 2020).

### Molecular Mechanisms Associated With Enhanced Root Elongation in Azucena Under Stress Conditions

Root length, a contributing trait to RSA, strongly fluctuated between the two genotypes under the two conditions. Azucena not only had significantly longer roots than did IR64 under the two conditions, but also an increased rate of root growth and elongation induced by stress. With the onset of drought and drying of soil surface, plants capable of accessing water from deeper layers of soil by expanding their root systems can adapt more readily to water deficit stress, and maintain osmotic pressure. Root elongation is an important strategy used by tolerant genotypes to reach the moist layers of the soil (Comas et al., 2013). The root apical meristem, along with the elongation region, are responsible for root elongation, and are located in zone 1 of the plants in the present study.

Our expression analysis led to the identification of 17 genes upregulated in Z1 of Azucena including *OsRBG1*, *OsRMC*, and *OsERF2*/*OsWR4*. Ectopic expression of *OsRBG1*, preferentially expressed in meristematic tissues, increases auxin accumulation and facilitates crown root development. *OsRBG1* overexpression increases root length, primarily due to elevated cell numbers rather than cell enlargement. Also, *OsRBG1* co-localizes with microtubules known to be involved in cell division, which may account for the increase in organ size (Lo et al., 2020). Rice lines with *OsRMC* silenced had shorter primary roots and showed reduced total length of adventitious root (Jiang et al., 2007). *OsRMC*-overexpressing rice seedlings under Fe-deficient showed higher total root length than those of the wild types (Yang et al., 2013). Finally, *OsERF2*/*OsWR4* is required for the control of root architecture by regulating the expression of pivotal genes involved in rice root development. Its mutant lines showed shorter roots (Xiao et al., 2016).

Moreover, seven over-expressed genes (under both conditions) were found in the Azucena, including *OsDEL1*/*OsPLL12* and *OsDOF7*/*11*. Elongation of roots does not occur in transgenic rice seedlings with mutations in *OsDOF7*/*11* due to a reduced sucrose uptake (Wu et al., 2018). *OsDEL1*/*OsPLL12* is expressed predominantly in elongating tissues and is also involved in the maintenance of normal cell division. Mutation in *OsDEL1*/*OsPLL12* exhibited a clear 50% reduction in root length relative to the wild type (Leng et al., 2017).

### Water Stress Enhances Lateral Roots Formation in Both Genotypes, but More So in Azucena

It appears that expansion of the LR system is used as a strategy by both genotypes in facing water shortage, but it is employed to a greater extent by Azucena. LRs constitute a large proportion of the root system in total length and number, and are responsible for the greatest amount of water and nutrient uptake from the soil (Gowda et al., 2011). LR formation is influenced by the soil environment, and exhibits a high level of plasticity (Wangenheim et al., 2020). Proliferative roots have a relatively high-water uptake efficiency in water deficient soils (Ye et al., 2018). Although there are few studies on root branching patterns under abiotic stresses, increased lateral root density in response to drought has previously been reported in an article examining 11 Egyptian rice genotypes (Hazman and Brown, 2018).

Our transcriptomic approach revealed 18 genes significantly upregulated in Azucena roots that are related to LR growth and development, included *OsACS*, *OsARF16*, *OsPTR9*, *OsMADS27*, *OsHO1*/*OsSE5*, *OsRAA1*, and *OsAUX*/*LAX1*. Interestingly, all 18 DEGs were significantly induced in both zones 2 and 3, which are enriched for LR growth and development. Rice *OsACS* mutants fail to promote LR growth and elongation especially in response to inorganic phosphate deficiency, however, the most common morphological changes in response to inorganic phosphate deficiency was the increase in LR growth (Lee et al., 2019). Rice *ARF16*, along with several other genes involved in auxin biosynthesis and transport, control the phosphate-dependent LR formation (Shen et al., 2013). *OsPTR9*, when overexpressed in transgenic rice, results in promotion of LR growth and development and enhanced ammonium uptake, while its mutant lines have a lower number of LRs (Fang et al., 2013). *OsMADS27* strongly induces proliferation and the length of LRs when overexpressed (Liu et al., 2020) while under constitutive expression it significantly enhances the formation of lateral roots in a nitrate-dependent manner (Chen et al., 2018). Overexpression of *OsHO1*/*OsSE5* enhances LR formation in rice (Chen et al., 2012). *OsRAA1* is expressed in LR primordia and the pericycles of the branch zones. Its overexpression led to longer LRs (Han et al., 2008). *OsAUX*/*LAX1* was upregulated in Azucena roots in response to stress. In Arabidopsis, auxin influx via *AUX1*, and its close homologs *LAX1*, *LAX2* and *LAX3*, three transmembrane proteins with a highly conserved permease activity, participate in LR formation and emergence (Ugartechea-Chirino et al., 2009).

### Cell Wall Thickening in IR64 Under Stress May Reduce Root Growth

Cell wall thickening occurred in meristematic cells of IR64 roots in response to water stress. Cell wall dynamics and modifications under abiotic stress are variable depending on the genotype, the organ, the tissue, and the intensity of the stress (Le Gall et al., 2015). Cell wall thickening, by granting further rigidity to the wall, limits water loss. It also promotes water transport and maintains cell turgor pressure, in addition to restricting cell movement, elongation and enlargement by reducing cell wall flexibility (Yamaguchi et al., 2010; Liu et al., 2018). Thus, by enhancing cell wall thickening for insulation purposes as a mean of mitigating stress, the sensitive IR64 genotype may have reduced its capacity for root elongation to extract water from deeper layers of the soil. To strengthen this hypothesis, a list of genes known to take part in cell wall growth and development was prepared and their expression patterns were surveyed in our transcriptomic data. This led to the identification of 32 genes upregulated in Z1 of IR64 including three members of the *CAD* gene family (*OsCAD3*, *OsCAD8D*, and *OsCAD9*) along with *OsMYB52*/*54*, *OsSUS3*, *OsPRX1*, and *BSH1*/*OsCYP96B4*. CADs are an important group of enzymes which catalyzed lignin biosynthesis. In particular, the involvement of *OsCAD3* in lignin biosynthesis was recently reported in loss-of-function mutants with reduced levels of lignin (Tobias and Chow, 2005; Park et al., 2018). *OsMYB52*/*54*, is a secondary wall-associated gene. Its product acts downstream from *CEF1*/*OsMYB103L*, a master switch that regulates the regulatory cascade of secondary wall biosynthesis in rice. Loss-of-function mutation in *CEF1*/*OsMYB103L* reduces cellulose content and decrease thickness of the cell walls (Ye et al., 2015). *OsSUS3* participates in carbon-partitioning regulation for the direct biosynthesis of cellulose and callose. Overexpression of *OsSUS3* reduces cellulose crystallinity and increases cell wall polysaccharide deposition and thickness in transgenic rice (Fan et al., 2020). *OsPRX1*, a class III peroxidase, catalyzes a variety of reactions in cell wall formation and modification, including the cross-linking of structural polysaccharides, oxidative lignin polymerization, and ROS production, all of which are involved in modulation of the mechanical properties of cell walls (Passardi et al., 2004). *BSH1*/*OsCYP96B4* encodes a cytochrome P450 monooxygenase that functions mainly in secondary cell wall formation and thickening. *bsh1* mutants have been shown to have reduced levels of cell wall components such as cellulose and xylose (Wang et al., 2016; Jiang et al., 2020).

### Members of the *NAC, AP2/ERF, AUX/IAA, EXPANSIN, WRKY*, and *MYB* Gene Families May Play Important Roles in Root System Architecture and Drought Adaptation

Differential expression of members of a gene family across three root zones of both genotypes under both normal and stress conditions suggested their potential roles in RSA and drought adaptation. For example, 19 members of the *NAC* gene family were upregulated in Azucena roots in response to stress, five of which are known to be involved in rice RSA and drought stress response and tolerance. Overexpression of *OsNAC5* (Jeong et al., 2013) and *OsNAC10* (Jeong et al., 2010) in rice roots enlarge root diameter and enhance drought tolerance and grain yield under field conditions. Overexpression of *ONAC3* (*SNAC3*) in rice enhances tolerance to drought and oxidative stress while its suppression increases sensitivity to stress (Fang et al., 2015). *ONAC106* plays roles in osmotic stress-responsive signaling and plant architecture (Sakuraba et al., 2015). Mutation in *ONAC54* (*OsRIM1*) leads to shorter roots in rice (Yoshii et al., 2010). *ONAC58* (*OsNAP*)-overexpressing rice enhances tolerance to drought (Chen et al., 2014) and shows longer roots (Zhou et al., 2013). Downregulation of *ONAC58* lead to reduction in root growth and total root length, and fewer root tips (Lu et al., 2017).

Sixteen members of the *AP2/ERF* were significantly upregulated in Azucena in response to stress, among which six members are known to play roles in water stress tolerance and rice root development. Overexpression of *OsERF50* (*OsDREB6*, *AP2/EREBP113*) increases tolerance to osmotic stress in transgenic rice, whereas, *OsERF50* RNAi knockdown lines are more sensitive to stress than the wild type (Ke et al., 2014). Overexpression of *OsERF48* produces a longer and denser root phenotype. Under drought condition the transgenics exhibit a more vigorous root growth and a higher grain yield (Jung et al., 2017). The role of *OsERF101* in improving rice yield under drought, and its predominant expression in reproductive tissues have been reported (Jin et al., 2018) while in the present study *OsERF101* expression was detected in all 3 root zones in both genotypes and was induced by stress in Azucena. *OsERF95* (*OsSta2*)-overexpressing lines show more tolerance to osmotic stress (Kumar et al., 2017). *OsERF93* (*ERF1*) is involved in the induction of RSOsPR10 [root specific *Oryza sativa* pathogenesis-related (PR) protein 10] which plays a role in protecting the inner vascular system in the root during drought stress (Takeuchi et al., 2011). *OsERF2*/*OsWR4* controls root architecture and affects primary root growth in rice, and mutant lines of the same gene have shorter roots (Xiao et al., 2016).

The expression level of three genes *EXPA2*, *EXPB11*, and *EXPA5* increased significantly in response to water stress in Azucena roots. *OsEXPA2* has been shown to be linked to a QTL for seminal root length under water deficit conditions (Yang et al., 2004). The specific expression of *OsEXPA2* in the pericycle in the root-hair zone and the lateral and adventitious root primordia has also been reported (Cho and Kende, 1998).

We observed a significant upregulation of 6 members of this family under stress in Azucena. Of these, *OsIAA18* participates in tolerance against drought stress in rice (Song et al., 2009). A recent study in rice seedlings revealed *OsIAA26* and *OsIAA20* functioning in the reduction of inhibitory effects of ethylene on root growth and elongation (Chen et al., 2018). *OsIAA20* and *OsIAA23* participate in the maintenance of the root quiescent center during the primary, lateral and crown root development (Jun et al., 2011).

Six and seven members of *MYB* and *WRKY* gene families were upregulated in the Azucena roots. In rice roots, the expression of *OsWRKY11* is highly upregulated under mild drought stress, implying a role in drought avoidance (Kakar et al., 2016). *OsWRKY11* may also play a role in stimulating root growth, enhancing the plants ability to extract water from soil in water stress condition (Nuruzzaman et al., 2014). A reduction in the expression level of OsWRKY72 in del1 rice mutants leads to a reduction in root length and number (Leng et al., 2017). The constitutive expression of OsWRKY72 in transgenic Arabidopsis also affects root growth and stress tolerance (Song et al., 2010). OsWRKY3 has previously been identified as a drought responsive gene in rice (Berri et al., 2009; Yu et al., 2017).

Our results showed that *HSPs* and *HSF*s were specifically abundant in IR64 root in response to a 14-day water withholding compared to Azucena. It reveals the important roles of *HSP* and *HSF* families in IR64 adaptive strategies when confronting water-deficit stress. Plant *HSP*s as chaperones and *HSF*s as *HSP*s’ transcriptional regulators have been identified for playing a vital role in protecting proteins that might lose their potential functional conformation during biotic and/or abiotic stress (Khan et al., 2019). Also, *HSP*s and *HSF*s detoxify the reactive oxygen species by positively regulating the antioxidant enzymatic system and thus enhance membrane stability when plants face oxidative stress (Khan et al., 2019). Consequently, *HSP*s and *HSF*s might be considered as the main components of IR64 adaptation strategy against oxidative stress. A study on chickpea reported that *HSP*s were down-regulated in drought-tolerant genotypes while they were abundant in drought-sensitive genotypes (Sato and Yokoya, 2008). Theses finding might suggest that *HSP*s respond to drought in a genotype specific manner.

### Integrating Transcriptome Profiling and Meta-Quantitative Trait Loci Analysis: Identification of Novel Root System Architecture-Associated Candidate Genes

Many QTLs related to rice RSA traits were identified so far, including a number of genes which their expressions were not studied yet under different genotypes and conditions. They may be valuable resources for integrating with transcriptome profiling of RSA-contrasted genotypes and finding novel associated candidate genes. Meta-analysis of QTLs is a powerful statistical technique for reducing the confidence interval of QTL position through refining and confirming QTL positions on a consensus map via mathematical models. In Meta-QTL analysis, a number of independent QTL studies performed across different genetic backgrounds and environments, are combined to determine the number of true QTLs enabling researchers to position consensus QTLs with a greater precision, and to further reduce the intervals and the number of candidate genes (Coudert et al., 2010; Comas et al., 2013; Daryani et al., 2021). QTL meta-analysis method was initially developed by Goffinet and Gerber (2000) using maximum likelihood estimation and was then improved by Veyrieras et al. (2007). The integration of RSA Meta-QTLs analysis with transcriptome profiling may assist in generating a more reliable list of potential candidate genes involved in RSA. In this study, a total of 132 QTLs controlling RSA traits under drought and normal conditions, including 82 and 50 from IR64 × Azucena and Azucena × other rice genotypes populations, respectively, were collected and 31 and 23 significant Meta-QTLs were identified according to Daryani et al. (2021) method. The confidence interval (CI) of the identified M-QTLs was obtained as 0.11–17.67 cM and 0.055–5.2 which were 2.52 and 3.07 times narrower than the mean CI of the original QTLs belonged to IR64 × Azucena populations and Azucena × other rice genotypes populations, respectively. Also, the physical intervals of most of the obtained M-QTLs (14 M-QTLs and 10 M-QTLs belonged to IR64 × Azucena populations and Azucena × other rice genotypes populations, respectively) were below 1 Mb.

To narrow down the candidate genes, only the 1047 common genes between two sets of populations (see section “Materials and Methods”) were considered for searching in our transcriptome data, with 291 of these corresponding to DEGs in our study. These included 125, 159, and 130 DEGs in zones 1, 2, and 3, respectively. Some were common between all three zones while others were zone-specific, some had functional descriptions, while others lacked functional annotations and thus represent novel candidates with potential roles in RSA related pathways.

*OsACO1*, a highly upregulated gene in Z1 of Azucena roots in response to water stress, was located on meta-QTLs on chromosome 9 which controls the deep-rooting ratio trait. This gene is known to be involved in ethylene biosynthesis and salt stress response. Despite a lack of information on the role of *ACO1* in root traits, its positive role in internode elongation in rice has been reported (Iwamoto et al., 2010). *OsFbox490* (*Os09g0449600*), an F-box domain protein, was also found on chromosome 9, upregulated in Z1 of Azucena. A number of QTLs, related to maximum root length, are associated with a small region on chromosome 9 in rice. Containing three genes encoding F-box domain proteins, one Meta-QTL with a confidence interval of 20 kb were detected on rice chromosome 9 from resolving 14 QTLs for maximum root length (Courtois et al., 2009).

Interestingly, genes (adjacent to each other) located on three regions on chromosomes 4, 7, and 9 showed significant specific co-upregulation in Z1 of Azucena root. The regions belonged to the overlapped Meta-QTLs which control several root traits under drought (chromosome 4: RDW and RN; chromosome 7: RSR and DRW; chromosome 9: DRR). It was also noteworthy that only 2, 3 and 2 genes on chromosomes 4, 7, and 9, respectively, showed differential expression in zones 2 and 3, with the rest being specific to Z1. Specific expression of these genes in Z1, encompassing meristematic and elongating cells, reinforces the hypothesis on their roles in RSA trait determination. For example, *OsAMI1*/*2*, found on chromosome 4, encodes an enzyme involved in the biosynthesis of indole-3-acetamide to indole acetic acid. Auxins, as plant root forming hormones, promote root initiation, growth, development and branching (Overvoorde et al., 2010). Auxins are also involved in the control of cell division and elongation in root tips (Takatsuka and Umeda, 2014). Moreover, the role of *OsAMI1*/*2* in rice root hair development has also been reported (Wang et al., 2017). *Os09g0436500*, which is also located on chromosome 9, which controls the deep rooting ratio, is involved in the biosynthesis of isoleucine and valine. Reduction in the rate of isoleucine biosynthesis has been reported to lead to defects in both cell division and expansion processes during root development in Arabidopsis. Reduction in the number of root meristematic cells and the length of roots and root hairs has also been associated with mutations in isoleucine biosynthesis genes (Yu et al., 2013). The lack of functional annotation for the majority of these genes would represent them as potential novel candidates involved in RSA modification and response to water stress condition.

It was interesting to note that 17 of the highly upregulated DEGs in Z1 of Azucena (cluster 3, Figure 5A) were also co-localized with Meta-QTLs including *Os01g0253800*, *Os01g0257300* (*OsRAA1*), *Os01g0259966*, *Os04g0110100*, *Os04g0117900* (*OsAMI1*/*2*), *Os04g0108300*, *Os04g0111900* (*PIC22*), *Os04g0113000*, *Os07g0591700*, *Os07g0637300* (*OsHRK1*/*PDK*), Os07g0648300, *Os07g0650100*, *Os07g0594400*, *Os09g0436000*, *Os09g0442400*, *Os09g0447300* (*CYP76L1*/*Cytochrome P450 76L1*), and *Os09g0437500*. The expression pattern of five of these (*Os04g0110100*, *Os07g0650100*, *Os07g0637300*, *Os09g0437500*, and *Os09g0436000*) was also confirmed by qRT-PCR. These 17 candidate genes are located on meta-QTLs of chromosomes 1, 4, 7, and 9, controlling several root traits under drought stress (chromosome 1: RN, chromosome 4: RDW and RN; chromosome 7: RSR and DRW; chromosome 9: DRR).

## Conclusion

Detailed analyses of the transcriptome responses of three consecutive root tip zones to water stress in two contrasting genotypes of rice, IR64 (a susceptible and shallow-rooting genotype) and Azucena (a tolerant and deep-rooting genotype) lead to the identification of a number of DEGs and differential pathways involved in RSA and water stress adaptation. Our results revealed that Z1 of Azucena was specifically enriched for genes involved in cell cycle and division while in IR64 root, responses to oxidative stress were strongly enriched. It may suggest main intrinsic differences between Azucena and IR64 to maintain root growth under water stress condition. Also, a number of highly upregulated DEGs were identified in Z1 of Azucena involved in RSA development and water-stress adaptation. Our phenotypic analysis revealed that Azucena not only had significantly longer roots than IR64 under the two conditions, but also an increased rate of root growth and elongation induced by stress. It appears that expansion of the LR system is used as a strategy by both genotypes in facing water shortage, and to a greater extent by the upland tolerant genotype Azucena. By enhancing meristematic cell wall thickening for insulation purposes as a means of confronting stress, the sensitive IR64 genotype may have reduced its capacity for root elongation to extract water from deeper layers of the soil. In this study 28, 18, and 32 DEGs which may be involved in root elongation, lateral root development and cell wall formation and modification, respectively, were introduced. Furthermore, several members of gene families such as *NAC*, *AP2/ERF*, *AUX/IAA*, *EXPANSIN*, *WRKY*, and *MYB* emerged as main players in RSA and drought adaptation, while in IR64 root tip, members of *HSP* and *HSF* gene families were enriched. Integrating Meta-QTL analysis and transcriptome profiling revealed that 288 DEGs were co-localized with RSA QTLs previously reported under drought and normal conditions from two bi-parental populations. Considering the lack of functional annotation for the majority of these transcripts, they are reported here as novel candidate genes potentially involved in RSA modification and response to water stress conditions. We also found three co-expressed regions on Meta-QTLs on chromosomes 4, 7, and 9 (controlling the RDW and RN, the RSR and DRW, and DRR traits, respectively) specifically upregulated in Z1 of Azucena including 9, 7, and 9 genes, respectively. Lastly, it was interesting to note that 13 of the highly upregulated DEGs in Z1 of Azucena were also co-localized with Meta-QTLs. This finding warrants further research into their possible roles in drought adaptation and RSA. Overall, our analyses presented several major molecular differences between Azucena and IR64, which may partly explain their differential root growth responses to water stress. It appears that Azucena avoided water stress through enhancing growth and root exploration to access water, whereas IR64 with a shallow root system might mainly rely on cell insulation and antioxidant system to resist stress.

## Data Availability Statement

The original contributions presented in the study are publicly available. This data can be found here: National Center for Biotechnology Information (NCBI) BioProject database under accession number PRJNA716593.

## Author Contributions

GHS and SA designed the experiment. GHS supervised the experiment and revised the manuscript. SA prepared the samples and wrote the manuscript. MG, ArS, SA, and AmS analyzed the data. PD and Z-SS performed the Meta-QTL analysis. SA, PK, NH, PY, and AM performed the microscopic analysis. LF, SA, ZG, and MK performed the qRT-PCR section. PH, SI, and MM cooperated in data interpretation and manuscript revision. All authors contributed to the article and approved the submitted version.

## Conflict of Interest

The authors declare that the research was conducted in the absence of any commercial or financial relationships that could be construed as a potential conflict of interest.

## Publisher’s Note

All claims expressed in this article are solely those of the authors and do not necessarily represent those of their affiliated organizations, or those of the publisher, the editors and the reviewers. Any product that may be evaluated in this article, or claim that may be made by its manufacturer, is not guaranteed or endorsed by the publisher.

## Footnotes

1
https://plants.ensembl.org/info/data/ftp/index.html

2
https://funricegenes.github.io/

3
https://shigen.nig.ac.jp/rice/oryzabase/

4
https://rapdb.dna.affrc.go.jp/

5
http://ricephylogenomics-khu.org/tf/dbsearch.php

6
https://www.uniprot.org

7
http://archive.gramene.org/markers/microsat/

8
https://plants.ensembl.org/biomart/martview/

9
https://www.idtdna.com/pages/tools/oligoanalyzer

10
https://plants.ensembl.org/info/website/ftp/index.html
