# Supplementary material for: Genome-Wide Expression Analysis of Root Tips in Contrasting Rice Genotypes Revealed Novel Candidate Genes for Water Stress Adaptation
Source: Front Plant Sci. 2022 Feb 21;13:792079. doi: 10.3389/fpls.2022.792079 (PMC8899714; doi:10.3389/fpls.2022.792079)
Supplement: Supplementary file 1 [file Data_Sheet_1.zip › Supplementary Material/Supplementary Figures (1-10) and Supplementary Tables (1-7 &10-12).docx]

**Supplementary Information**

**List of Figures**

1. **Figure S1**. Number of macroscopic lateral roots along a 30-centimeter section (from tip) of the longest crown root.
2. **Figure S2.** Summary of read mapping statistics for each replicate of each sample.
3. **Figure S3.** Distance matrix heatmap between all samples.
4. **Figure S4.** Heatmap representing the expression patterns of genes with roles in rice root length, lateral root formation and cell wall biogenesis and modifications significantly changed in both genotype roots under stress.
5. **Figure S5**. Heatmaps representing the expression patterns of members of (A) AP2/ERF, (B) NAC, (C) WRKY, (D) EXPANSIN, (E) AUX/IAA, and (F) MYB gene families which had significant interactions between genotypes and conditions (p ≤ 0.05) by two-way ANOVA test.
6. **Figure S6**. Heatmaps representing the expression patterns of members of (A) ARF, (B) bHLH, (C) bZIP, (D) LEA, (E) EIN, (F) GRAS, (G) G2-like, (H) ARID, (I) GRF, and (J) CLV gene families which had significant interactions between genotypes and conditions (p ≤ 0.05) by two-way ANOVA test.
7. **Figure S7**. Heatmaps representing the expression patterns of members of (A) HSF, (B) HSP, (C) LBD, (D) MADS, (E) MYB-related, (F) RR, (G) G2-like, (H) SWEET, (I) WOX, and (J) TIP&PIP gene families which had significant interactions between genotypes and conditions (p ≤ 0.05) by two-way ANOVA test.
8. **Figure S8.** The obtained Meta-QTLs located on their chromosomal regions along with the number of total genes and differentially expressed genes extracted from the marked regions belonged to overlapped regions.
9. **Figure S9.** The expression patterns of the differentially expressed genes extracted from overlapping Meta-QTLs belonged to zone 2 and zone 3.

**List of Tables**

1. **Table S1.** Descriptive statistics for the phenotypic traits under well-watered and water-deficit stress conditions.
2. **Table S2.** % changes between control and water-deficit stress conditions.
3. **Table S3.** Samples used in this study.
4. **Table S4.** Enriched biological processes for identified gene sets (silent, low frequency, intermediate frequency and constitutive).
5. **Table S5.** Enriched biological processes for identified gene sets (genotype, zone and condition specific).
6. **Table S6.** The significant enriched biological processes (BP) belonged to intersections of DEGs across the 6 gene lists.
7. **Table S7.** Enriched biological processes for identified gene sets from the hierarchical clustering of DEGs based on the fold-change values.
8. **Table S10.** Summary of QTL studies included in the meta-analysis for 2 populations, IR64 × Azucena populations and Azucena × other rice genotypes populations.
9. **Table S11.** List of QTLs associated with root morphological traits under well-watered and water stress conditions**.**
10. **Table S12:** List of obtained Meta-QTLs from meta-analysis of the collected QTLs associated root system architecture traits under normal and water stress conditions.

- **Supplementary Figures**

**Figure S1**. Number of macroscopic lateral roots along a 30-centimeter section (from tip) of the longest crown roots of two genotypes of rice, contrasting in RSA and water stress tolerance, IR64, the shallow rooting and sensitive genotype and Azucena, the deep rooting and tolerant genotype, grown in soil-filled root boxes in response to a 14-day water-deficit stress compared to well-watered samples.Values represent mean ± S.E. from six independent samples. The different letters indicate significant difference (p ≤ 0.05) by Duncans test. IRn: IR64 genotype under normal condition, IRs: IR64 genotype under water-deficit stress condition, AZn: Azucena genotype under normal condition, AZs: Azucena genotype under water-deficit stress condition.

**Figure S2.** Summary of read mapping statistics for each replicate of each sample (there were no failed-QC reads). IR: IR64; AZ: Azucena; Z: zone; n: normal condition; s: stress condition; r: biological replicate.


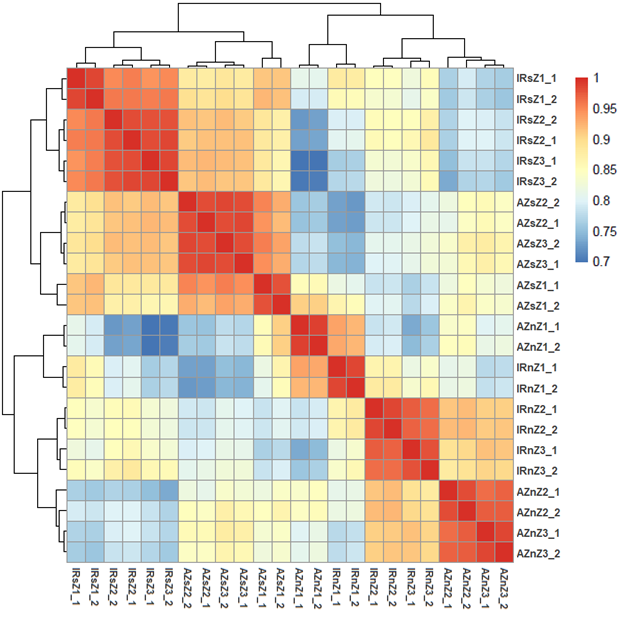


**Figure S3.** Distance matrix heatmap between all samples. Pearson correlation coefficients based on gene expression levels (FPKMs) between each pair of samples were shown as a heatmap and represented the consistency between biology replicates of each sample. A set of 24742 genes (about 65% of all identified annotated genes) expressed in at least one set of doublet replicates (FPKM>1) were used in this analysis.


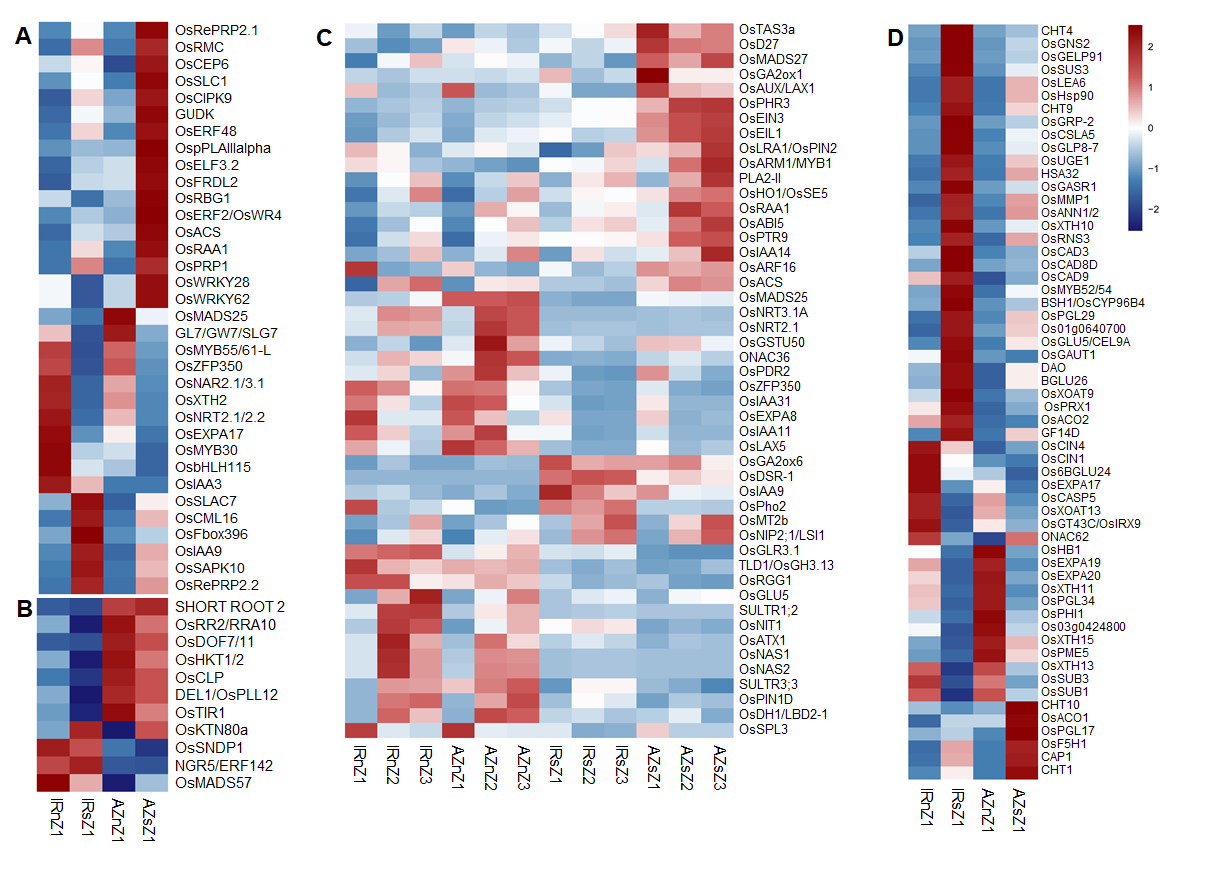


**Figure S4.** (A) Heatmap representing the expression patterns of genes with roles in rice root length significantly changed in root zone 1 of both genotypes under stress condition (p-value cut-off ≤ 0.05) analyzed by two-way ANOVA test. (B) Expression pattern of genes with roles in rice root length showed significant more values (p-value cut-off of ≤0.05, analyzed by t-test between genotypes in both conditions) in both genotypes, root zone 1, under both conditions. (C) Heatmap indicating the expression patterns of genes involved in lateral root formation, growth and development, induced in response to stress (significant interactions between genotypes and conditions (p-value cut-off ≤ 0.05) analyzed by two-way ANOVA test). (D) Heatmap representing the expression patterns of genes involved in cell wall biogenesis and modifications induced in zone 1 under stress (significant interactions between genotypes and conditions (p-value cut-off ≤ 0.05) analyzed by two-way ANOVA test).


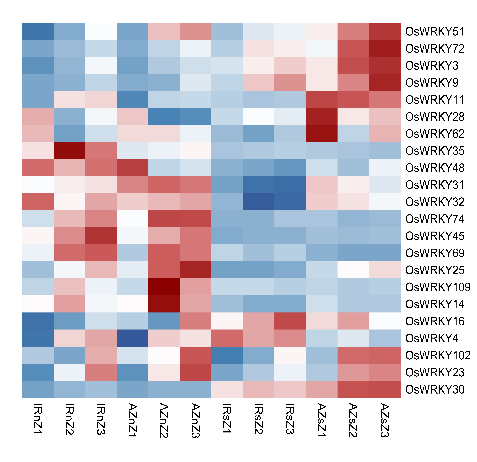

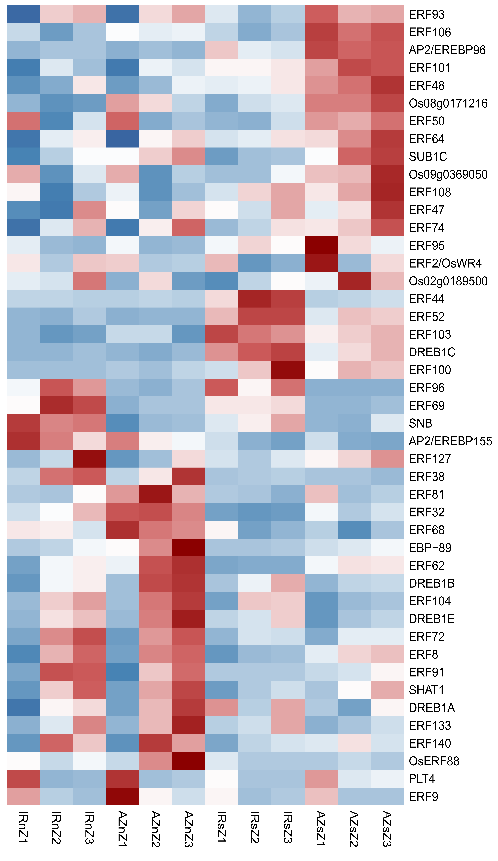

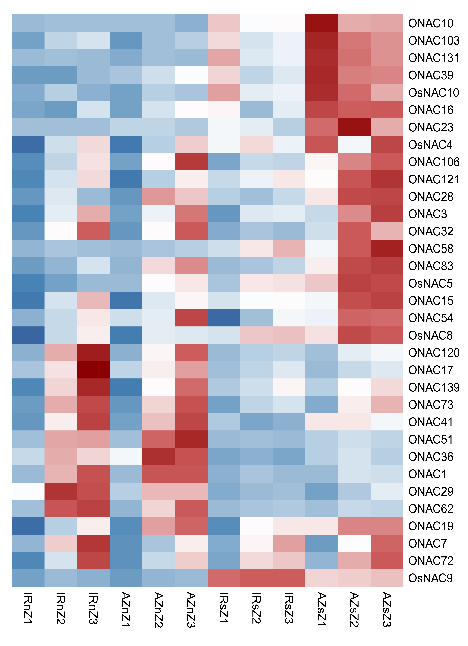


**B**

**C**

**A**

**F**


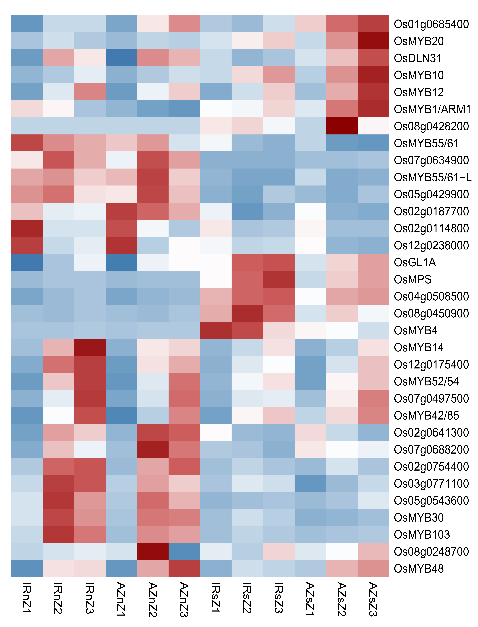


**E**


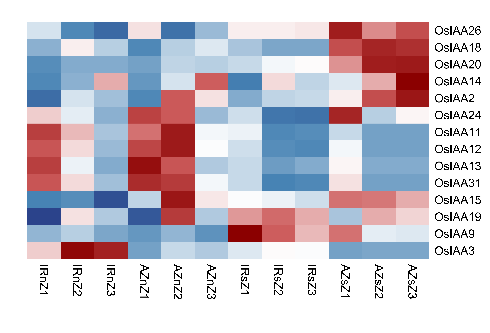


**D**


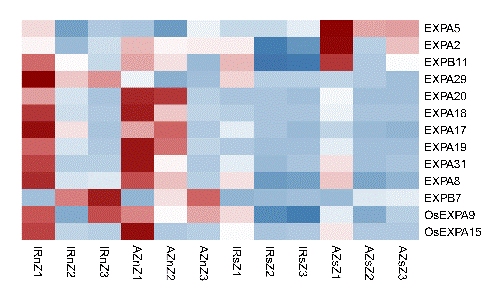


**Figure S5**. Heatmaps representing the expression patterns of members of (A) AP2/ERF, (B) NAC, (C) WRKY, (D) EXPANSIN, (E) AUX/IAA, and (F) MYB gene families which had significant interactions between genotypes and conditions (p ≤ 0.05) by two-way ANOVA test.


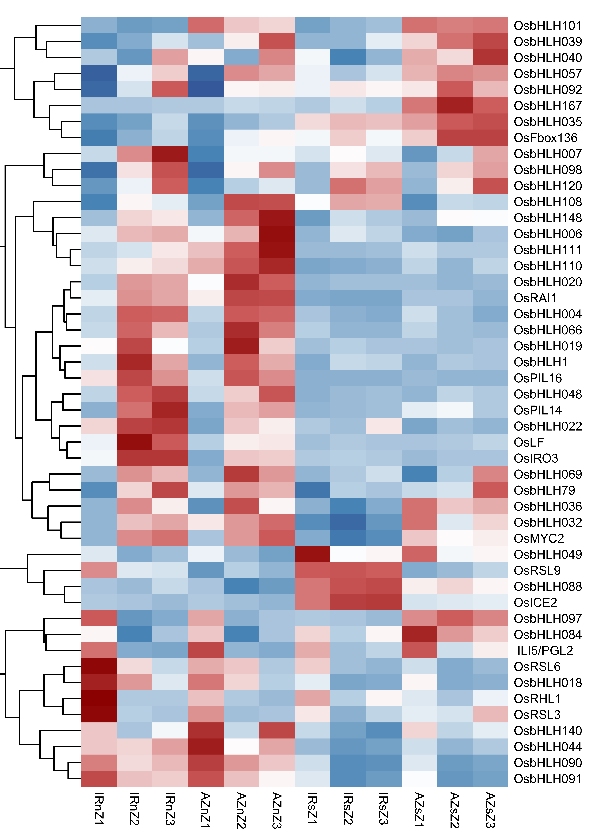

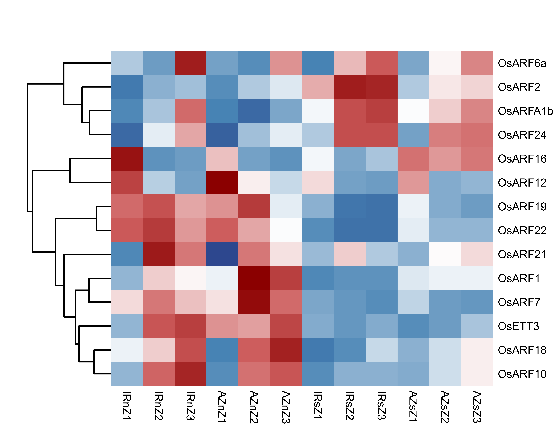

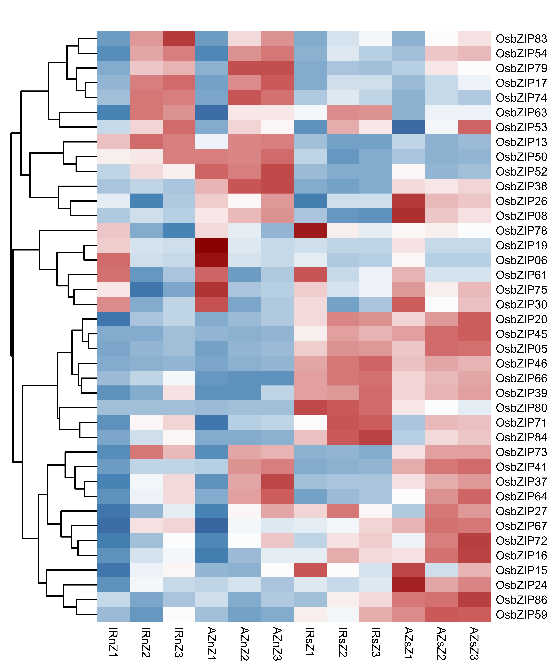


**B**

**C**

**A**

**D**


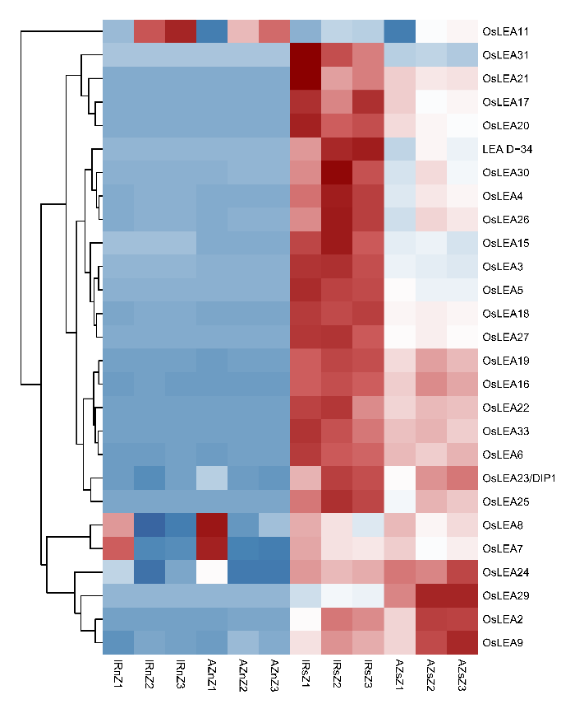


**G**


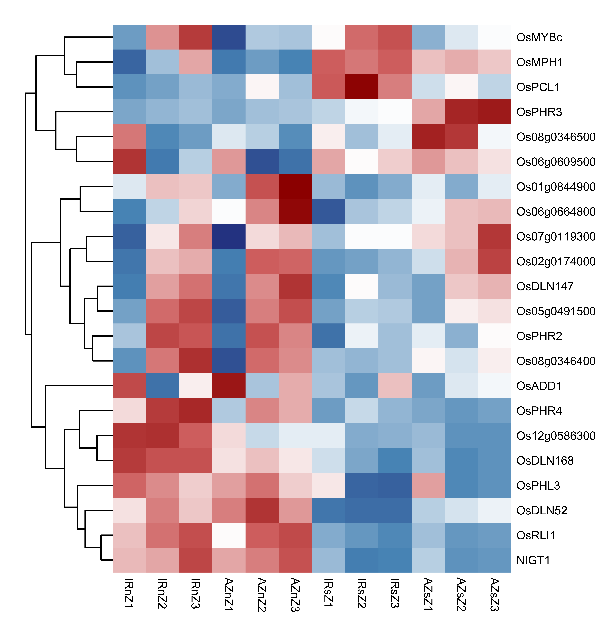


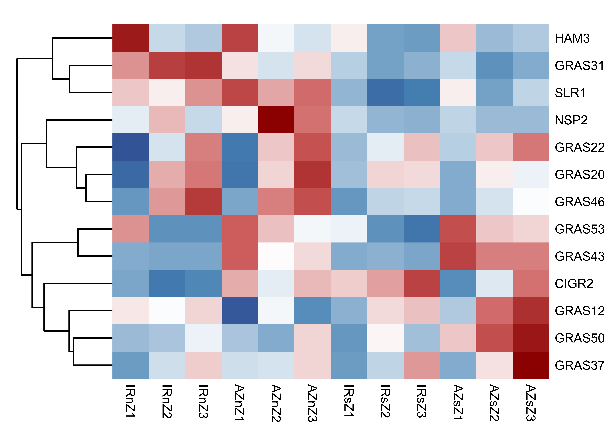


**F**


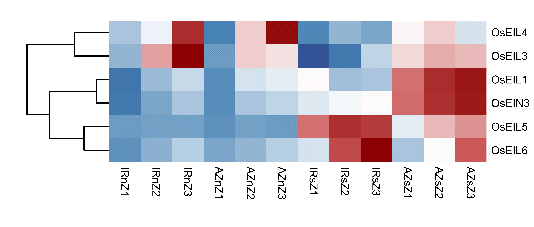


**E**


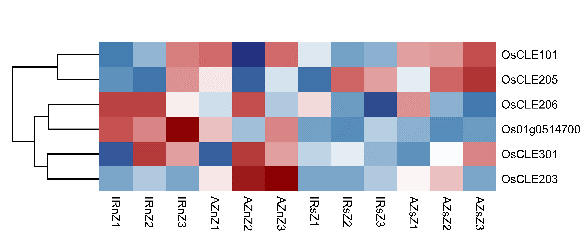


**H**

**I**

**J**


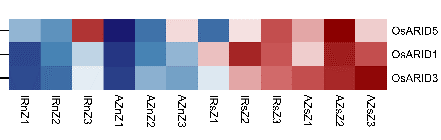

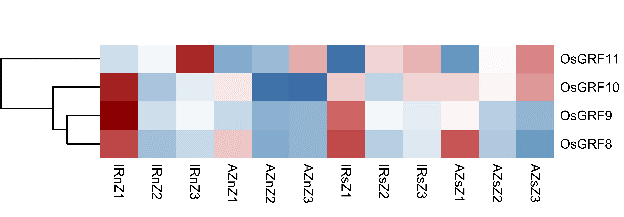


**Figure S6**. Heatmaps representing the expression patterns of members of (A) ARF, (B) bHLH, (C) bZIP, (D) LEA, (E) EIN, (F) GRAS, (G) G2-like, (H) ARID, (I) GRF, and (J) CLV gene families which had significant interactions between genotypes and conditions (p ≤ 0.05) by two-way ANOVA test.

**B**

**C**


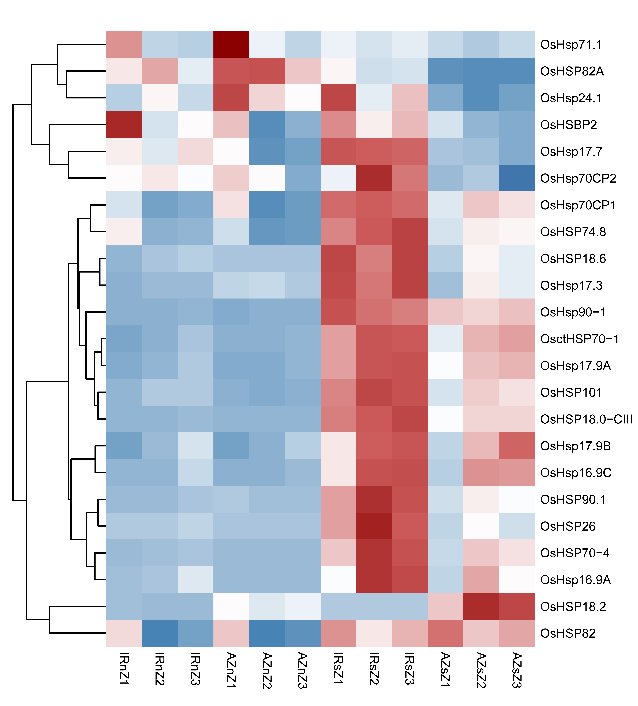

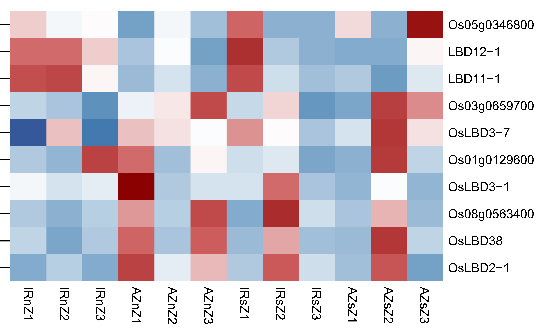

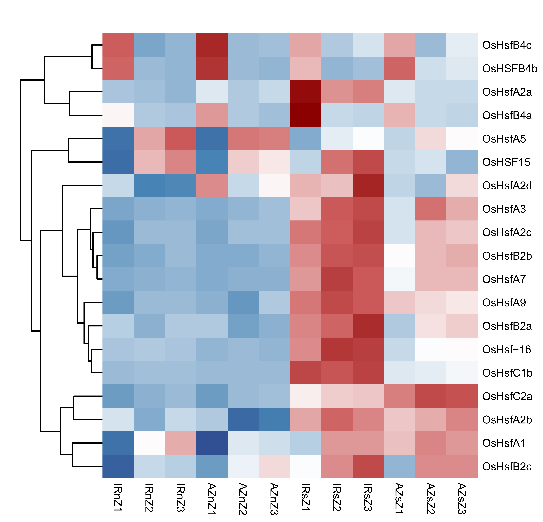


**A**


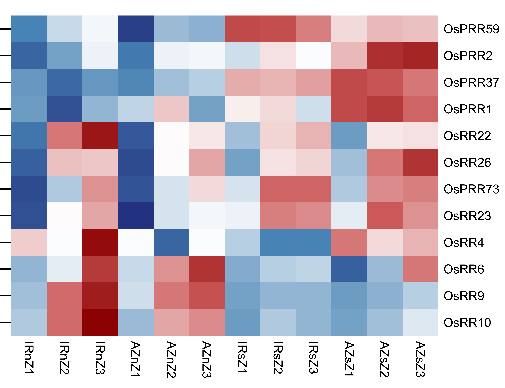


**F**


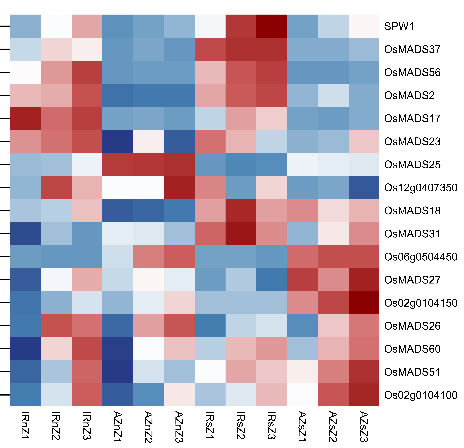


**D**

**E**


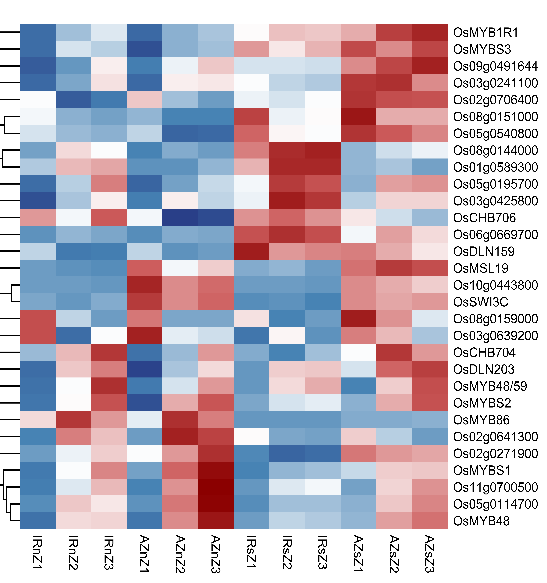


**G**


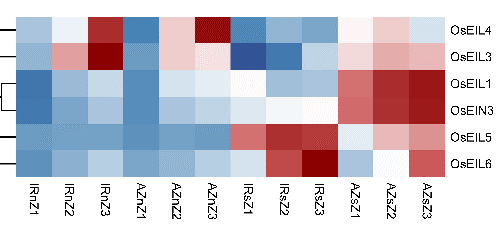


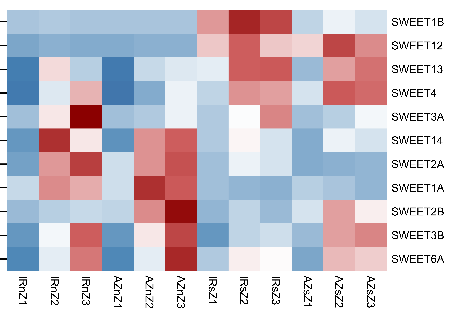


**H**

**J**


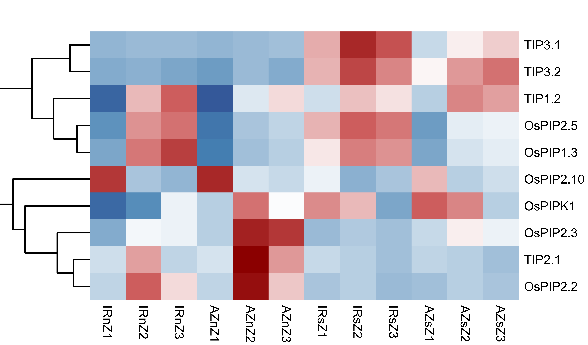


**I**


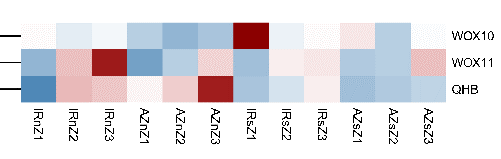


**Figure S7**. Heatmaps representing the expression patterns of members of (A) HSF, (B) HSP, (C) LBD, (D) MADS, (E) MYB-related, (F) RR, (G) G2-like, (H) SWEET, (I) WOX, and (J) TIP&PIP gene families which had significant interactions between genotypes and conditions (p ≤ 0.05) by two-way ANOVA test.


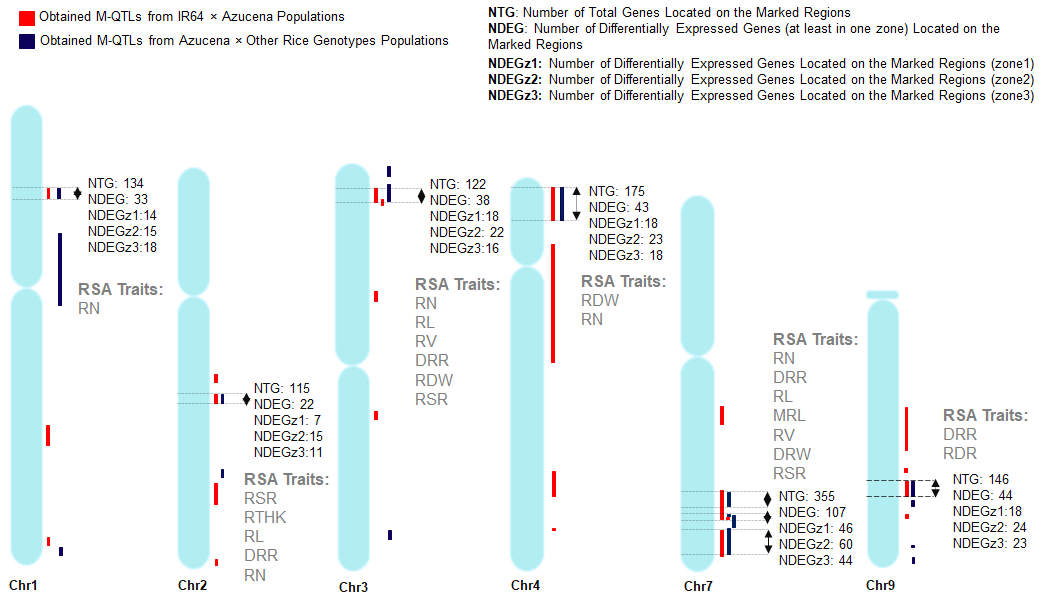


**Figure S8.** The obtained Meta-QTLs located on their chromosomal regions along with the number of total genes and differentially expressed genes extracted from the overlapped regions (arrow-marked areas). The root system architecture (RSA) traits associated with each marked region were listed; “Maximum root length (MRL)”, “deep rooting weight (DRW)”, “root number (RN)”, “root length (RL)”, “root thickness (RTHK)”, “root volume (RV)”, “root dry weight (RDW)”, “deep root ratio (DRR)”, “root to shoot ratio (RSR)”.

**B**

**A**


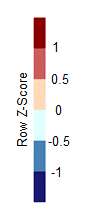

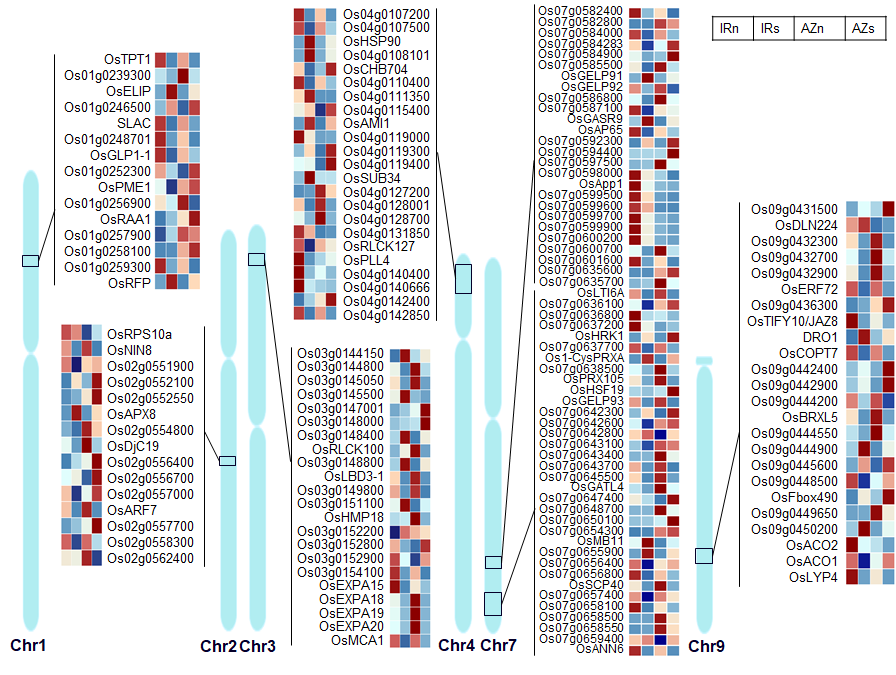

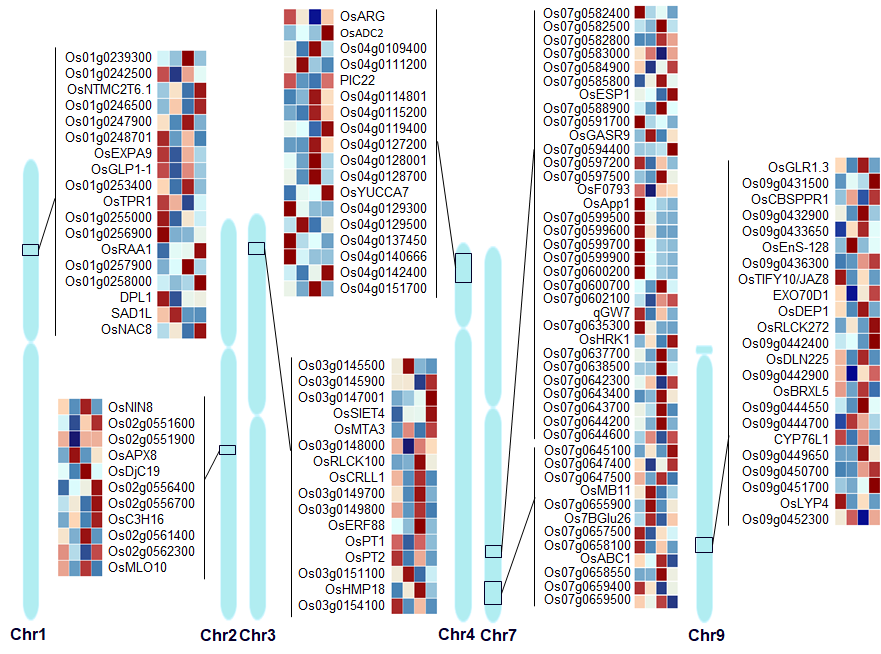


**Figure S9.** The expression patterns of the differentially expressed genes extracted from overlapping Meta-QTLs which had significant interactions (with the p-value cutoff of <0.05) between genotypes and conditions, belonged to zone 2 (A) and zone 3 (B), on their chromosomal regions.

- **Supplementary Tables**

**Table S1.** Descriptive statistics (mean, standard deviation (Sd), and standard error (Se); 6 biological replicates) for the phenotypic traits under well-watered (control) and water-deficit stress conditions. RWC: Relative water content; DW: Dry weight; LLR: Length of the longest root.

|  |  | RWC (%) | ShootDW (g) | RootDW (g) | LLR (cm) |
| --- | --- | --- | --- | --- | --- |
| IR64-Ctrl | Mean | 92.436 | 2.11 | 0.57 | 30.3 |
|  | Sd | 2.286 | 0.235 | 0.065 | 1.53 |
|  | Se | 0.933 | 0.096 | 0.026 | 0.62 |
| IR64-SWD-stress | Mean | 72.721 | 1.39 | 0.36 | 37.5 |
|  | Sd | 6.279 | 0.174 | 0.051 | 1.77 |
|  | Se | 2.563 | 0.071 | 0.021 | 0.71 |
| Azucena-Ctrl | Mean | 89.41 | 2.38 | 0.99 | 38.5 |
|  | Sd | 4.989 | 0.604 | 0.134 | 2.0 |
|  | Se | 2.037 | 0.246 | 0.054 | 0.84 |
| Azucena-SWD-stress | Mean | 80.16 | 1.82 | 0.86 | 61.3 |
|  | Sd | 5.56 | 0.386 | 0.102 | 3.42 |
|  | Se | 2.269 | 0.157 | 0.040 | 1.39 |

**Table S2.** % changes between control and water-deficit stress conditions (Stress vs. Control). RWC: Relative water content; DW: Dry weight; LLR: Length of the longest root. Asterisks indicate significant differences between conditions: * = p < 0.05; ** = p < 0.01; *** = p < 0.001; **** = p < 0.0001.

|  | RWC | ShootDW | RootDW | LLR |
| --- | --- | --- | --- | --- |
| IR64 | -21.33 *** | -34.3 **** | -36.31 **** | 23.64 **** |
| Azucena | -10.34 ** | -23.67 * | -13.42 * | 58.74 **** |

**Table S3.** Samples used in this study.

| **Sample** | **Tissue** | **Genotype** | **Condition** | **Rep.** | **TotalPairs** | **PoorQualitySequences** | **MappingRates** | **UniqueMappingRates** |
| --- | --- | --- | --- | --- | --- | --- | --- | --- |
| S01 | Zone1 | IR64 | Control | 1 | 17909774 | 0 | 95.07% | 83.99% |
| S02 | Zone1 | IR64 | Control | 2 | 16622386 | 0 | 94.68% | 87.11% |
| S03 | Zone1 | IR64 | Stress | 1 | 18817279 | 0 | 94.38% | 86.73% |
| S04 | Zone1 | IR64 | Stress | 2 | 14903161 | 0 | 94.16% | 86.09% |
| S05 | Zone1 | Azucena | Control | 1 | 19154392 | 0 | 96.36% | 89.66% |
| S06 | Zone1 | Azucena | Control | 2 | 16033318 | 0 | 96.37% | 90.00% |
| S07 | Zone1 | Azucena | Stress | 1 | 19036664 | 0 | 96.25% | 89.29% |
| S08 | Zone1 | Azucena | Stress | 2 | 21909684 | 0 | 96.30% | 89.51% |
| S09 | Zone2 | IR64 | Control | 1 | 22085061 | 0 | 94.35% | 87.88% |
| S10 | Zone2 | IR64 | Control | 2 | 18442412 | 0 | 94.65% | 88.56% |
| S11 | Zone2 | IR64 | Stress | 1 | 19541793 | 0 | 93.75% | 87.18% |
| S12 | Zone2 | IR64 | Stress | 2 | 15997552 | 0 | 94.33% | 88.05% |
| S13 | Zone2 | Azucena | Control | 1 | 17967206 | 0 | 96.22% | 90.76% |
| S14 | Zone2 | Azucena | Control | 2 | 18567097 | 0 | 96.05% | 90.57% |
| S15 | Zone2 | Azucena | Stress | 1 | 18117485 | 0 | 95.89% | 90.12% |
| S16 | Zone2 | Azucena | Stress | 2 | 18454173 | 0 | 96.02% | 90.20% |
| S17 | Zone3 | IR64 | Control | 1 | 18741615 | 0 | 94.06% | 86.87% |
| S18 | Zone3 | IR64 | Control | 2 | 21723153 | 0 | 93.95% | 87.28% |
| S19 | Zone3 | IR64 | Stress | 1 | 20557480 | 0 | 94.12% | 87.79% |
| S20 | Zone3 | IR64 | Stress | 2 | 17879193 | 0 | 93.79% | 87.11% |
| S21 | Zone3 | Azucena | Control | 1 | 19453503 | 0 | 95.96% | 90.40% |
| S22 | Zone3 | Azucena | Control | 2 | 21805906 | 0 | 95.95% | 90.51% |
| S23 | Zone3 | Azucena | Stress | 1 | 17739260 | 0 | 95.79% | 90.06% |
| S24 | Zone3 | Azucena | Stress | 2 | 20992488 | 0 | 95.78% | 90.08% |

**Table S4**. Enriched biological processes for identified gene sets (silent, low frequency, intermediate frequency and constitutive)

| **Gene set** | **GO term** | **Description** | **Annotated number in cluster** | **Annotated number in background** | **FDR** |
| --- | --- | --- | --- | --- | --- |
| Silent |  |  |  |  |  |
|  | GO:0032501 | Multicellular organismal process | 82 | 154/24075 | 8.1E-13 |
|  | GO:0022414 | Reproductive process | 57 | 114/24075 | 0.000000071 |
|  | GO:0022900 | Electron transport chain | 35 | 60/24075 | 0.0000089 |
|  | GO:0070882 | Cellular cell wall organization or biogenesis | 22 | 26/24075 | 0.000034 |
|  | GO:0006310 | DNA recombination | 30 | 61/24075 | 0.00082 |
|  | GO:0006721 | Terpenoid metabolic process | 13 | 13/24075 | 0.0025 |
|  | GO:0043086 | Negative regulation of catalytic activity | 27 | 59/24075 | 0.0041 |
| Less than 20% (Low frequency) | | | | | |
|  | GO:0032502 | Developmental process | 7 | 28/24075 | 0.00032 |
|  | GO:0007275 | Multicellular organismal development | 6 | 27/24075 | 0.0018 |
|  | GO:0000003 | Reproduction | 9 | 133/24075 | 0.032 |
| Bet. 20%-80% (Intermediate frequency) | | | | | |
|  | GO:0007275 | Multicellular organismal development | 26 | 27/24075 | 6.8E-09 |
|  | GO:0032502 | Developmental process | 26 | 28/24075 | 6.8E-09 |
|  | GO:0022900 | Electron transport chain | 27 | 60/24075 | 0.00016 |
|  | GO:0000272 | Polysaccharide catabolic process | 21 | 37/24075 | 0.00016 |
|  | GO:0006721 | Terpenoid metabolic process | 13 | 13/24075 | 0.00028 |
|  | GO:0070882 | Cellular cell wall organization or biogenesis | 17 | 26/24075 | 0.00032 |
|  | GO:0009725 | Response to hormone stimulus | 16 | 30/24075 | 0.0027 |
|  | GO:0042221 | Response to chemical stimulus | 66 | 301/24075 | 0.0049 |
|  | GO:0006310 | DNA recombination | 22 | 61/24075 | 0.0079 |
| More than 80% (Constitutive) | | | | | |
|  | GO:0008104 | Protein localization | 266 | 307/24075 | 0.027 |
|  | GO:0044248 | Cellular catabolic process | 285 | 337/24075 | 0.027 |
|  | GO:0023052 | Signaling | 361 | 447/24075 | 0.027 |
|  | GO:0045184 | Establishment of protein localization | 251 | 294/24075 | 0.027 |
|  | GO:0015031 | Protein transport | 251 | 294/24075 | 0.027 |
|  | GO:0042180 | Cellular ketone metabolic process | 377 | 480/24075 | 0.036 |
|  | GO:0016070 | RNA metabolic process | 782 | 1088/24075 | 0.036 |
|  | GO:0019752 | Carboxylic acid metabolic process | 370 | 473/24075 | 0.036 |
|  | GO:0044085 | Cellular component biogenesis | 206 | 239/24075 | 0.036 |

**Table S5.** Enriched biological processes for identified gene sets (genotype, zone and condition specific).

| **Gene set** | **GO term** | **Description** | | **Annotated number in cluster** | **Annotated number in background** | **FDR** |
| --- | --- | --- | --- | --- | --- | --- |
| **Silent**  Genotype specific |  |  |  | |  |  |
| **IR64** | GO:0012501 | Programmed cell death | 52/743 | | 532/24075 | 3.80E-10 |
|  | GO:0006915 | Apoptosis | 52/743 | | 532/24075 | 3.80E-10 |
|  | GO:0006310 | DNA recombination | 10/743 | | 61/24075 | 0.005 |
| **Azucena** | GO:0007275 | Multicellular organismal development | 7/483 | | 27/24075 | 0.00097 |
|  | GO:0032502 | Developmental process | 7/483 | | 28/24075 | 0.00097 |
|  | GO:0006721 | Terpenoid metabolic process | 5/483 | | 13/24075 | 0.0028 |
| Condition specific |  |  |  | |  |  |
| **Control** | GO:0032502 | Developmental process | 7/476 | | 28/24075 | 0.002 |
|  | GO:0006721 | Terpenoid metabolic process | 5/476 | | 13/24075 | 0.0044 |
| **Stress** | GO:0032501 | Multicellular organismal process | 22/865 | | 154/24075 | 0.00016 |
|  | GO:0022414 | Reproductive process | 17/865 | | 114/24075 | 0.00069 |
|  | GO:0032502 | Developmental process | 9/865 | | 28/24075 | 0.00069 |
|  | GO:0070882 | Cellular cell wall organization or biogenesis | 8/865 | | 26/24075 | 0.0021 |
|  | GO:0009856 | Pollination | 13/865 | | 114/24075 | 0.041 |
| Zone specific |  |  |  | |  |  |
| **Zone 1** | GO:0010033 | Response to organic substance | 9/699 | | 30/24075 | 0.00022 |
|  | GO:0009725 | Response to hormone stimulus | 9/699 | | 30/24075 | 0.00022 |
|  | GO:0007275 | Multicellular organismal development | 8/699 | | 27/24075 | 0.00071 |
|  |  |  |  | |  |  |
| **Zone 2** | --- | --- | --- | | --- | --- |
|  |  |  |  | |  |  |
| **Zone 3** | --- | --- | --- | | --- | --- |
| **Expressed (All)** |  |  |  | |  |  |
| Zone specific |  |  |  | |  |  |
|  |  |  |  | |  |  |
| **Zone 1** | GO:0032502 | Developmental process | 6/309 | | 28/24075 | 0.0014 |
|  | GO:0071554 | Cell wall organization or biogenesis | 10/309 | | 148/24075 | 0.0042 |
|  | GO:0007275 | Multicellular organismal development | 5/309 | | 27/24075 | 0.0042 |
|  | GO:0006310 | DNA recombination | 6/309 | | 61/24075 | 0.014 |
|  |  |  |  | |  |  |
| **Zone 2** | --- | --- | --- | | --- | --- |
|  |  |  |  | |  |  |
| **Zone 3** | --- | --- | --- | | --- | --- |

**Table S6.** The significant enriched biological processes (BP) belonged to intersections of DEGs (C01-C40, representing in upset plot in figure 3) across the 6 gene lists (IRsnZ1, IRsnZ2, IRsnZ3, AZsnZ1, AZsnZ2, and AZsnZ3).

| **Cluster** | **GOID** | **GOTerm** | **FDR** | **Nr. Genes** | **% Associated Genes** | **Associated Genes Found** |
| --- | --- | --- | --- | --- | --- | --- |
| C01 | GO:0009694 | jasmonic acid metabolic process | 0.029928 | 6 | 22.22222 | [LOC4324570, LOC4333201, LOC4334233, LOC4341907, LOC4344475, LOC4345762] |
| C01 | GO:0006542 | glutamine biosynthetic process | 0.017167 | 3 | 60 | [LOC4330649, LOC4333896, LOC4337272] |
| C01 | GO:0005200 | structural constituent of cytoskeleton | 0.010107 | 5 | 35.71429 | [LOC4326917, LOC4327550, LOC4333632, LOC4338790, LOC4343694] |
| C01 | GO:0006740 | NADPH regeneration | 0.036261 | 5 | 25 | [LOC4327239, LOC4329889, LOC4333117, LOC4335330, LOC4335430] |
| C01 | GO:0006098 | pentose-phosphate shunt | 0.030378 | 5 | 26.31579 | [LOC4327239, LOC4329889, LOC4333117, LOC4335330, LOC4335430] |
| C01 | GO:0009834 | plant-type secondary cell wall biogenesis | 0.001419 | 10 | 25 | [LOC4326326, LOC4327242, LOC4332646, LOC4337665, LOC4339617, LOC4345960, LOC4348270, LOC4350540, LOC9269038, LOC9272120] |
| C01 | GO:0015969 | guanosine tetraphosphate metabolic process | 0.042162 | 3 | 42.85714 | [LOC4332791, LOC4337890, LOC4345756] |
| C01 | GO:0006790 | sulfur compound metabolic process | 0.010107 | 28 | 11.11111 | [LOC4324574, LOC4325163, LOC4325710, LOC4325712, LOC4327425, LOC4329321, LOC4329692, LOC4330673, LOC4332456, LOC4335048, LOC4336847, LOC4338768, LOC4339646, LOC4340033, LOC4340140, LOC4340640, LOC4342614, LOC4345205, LOC4346775, LOC4347614, LOC4349123, LOC4349192, LOC4350083, LOC4350524, LOC4352747, LOC9267617, LOC9267826, LOC9272308] |
| C01 | GO:0016054 | organic acid catabolic process | 0.014003 | 15 | 13.76147 | [LOC4325145, LOC4326136, LOC4327232, LOC4328441, LOC4329692, LOC4334405, LOC4334698, LOC4337447, LOC4338691, LOC4338861, LOC4339131, LOC4345318, LOC4345787, LOC4345810, LOC9271676] |
| C01 | GO:1902299 | pre-replicative complex assembly involved in cell cycle DNA replication | 0.042162 | 3 | 42.85714 | [LOC107275673, LOC4331016, LOC4338176] |
| C01 | GO:0009651 | response to salt stress | 0.005419 | 15 | 15.625 | [LOC4325006, LOC4325078, LOC4329520, LOC4330838, LOC4332357, LOC4332688, LOC4333201, LOC4334553, LOC4336539, LOC4340462, LOC4343734, LOC4344028, LOC4346329, LOC4347172, LOC4349402] |
| C01 | GO:0047484 | regulation of response to osmotic stress | 0.015363 | 6 | 26.08696 | [LOC4325006, LOC4325078, LOC4329520, LOC4332688, LOC4334553, LOC4336539] |
| C01 | GO:0006979 | response to oxidative stress | 0.00359 | 30 | 11.76471 | [LOC4324554, LOC4325129, LOC4326273, LOC4326716, LOC4332174, LOC4332357, LOC4332925, LOC4333201, LOC4335202, LOC4335896, LOC4336354, LOC4336539, LOC4337728, LOC4337892, LOC4338417, LOC4339222, LOC4340052, LOC4341247, LOC4341861, LOC4342187, LOC4342593, LOC4344210, LOC4344397, LOC4345762, LOC4346141, LOC4346329, LOC4347520, LOC4348941, LOC4349240, LOC4349402] |
| C01 | GO:0009414 | response to water deprivation | 1.72E-05 | 17 | 25 | [LOC4325006, LOC4325078, LOC4326740, LOC4329520, LOC4330265, LOC4330838, LOC4332688, LOC4334553, LOC4336539, LOC4340462, LOC4344028, LOC4347172, LOC4349123, LOC4350448, LOC4350451, LOC4350452, LOC4350454] |
| C01 | GO:0009737 | response to abscisic acid | 3.07E-05 | 24 | 17.91045 | [LOC4324158, LOC4325078, LOC4329630, LOC4330265, LOC4330838, LOC4332374, LOC4332548, LOC4333017, LOC4333201, LOC4333435, LOC4339010, LOC4339454, LOC4340170, LOC4340462, LOC4342391, LOC4344028, LOC4345410, LOC4345807, LOC4347172, LOC4349062, LOC4350448, LOC4350451, LOC4350452, LOC4350454] |
| C01 | GO:0098869 | cellular oxidant detoxification | 0.006841 | 24 | 12.12121 | [LOC4324554, LOC4325129, LOC4326273, LOC4326716, LOC4332174, LOC4332925, LOC4335202, LOC4335896, LOC4336354, LOC4337728, LOC4337892, LOC4338417, LOC4339222, LOC4339397, LOC4341247, LOC4341861, LOC4342187, LOC4344045, LOC4344210, LOC4344397, LOC4346141, LOC4346329, LOC4347520, LOC4349240] |
| C03 | GO:0045492 | xylan biosynthetic process | 0.046292 | 4 | 15.38461 | [LOC4337222, LOC4341895, LOC4350174, LOC4351745] |
| C03 | GO:0006865 | amino acid transport | 0.004832 | 10 | 11.36364 | [LOC107275890, LOC4325094, LOC4327987, LOC4336662, LOC4338844, LOC4339758, LOC4340559, LOC4340751, LOC4344567, LOC4352231] |
| C03 | GO:0006071 | glycerol metabolic process | 0.019859 | 4 | 20 | [LOC4333407, LOC4337206, LOC4345965, LOC4346156] |
| C03 | GO:0042274 | ribosomal small subunit biogenesis | 6.11E-05 | 12 | 17.3913 | [LOC4324901, LOC4328064, LOC4328392, LOC4332813, LOC4335531, LOC4335547, LOC4336833, LOC4338485, LOC4344189, LOC4348523, LOC4350322, LOC4350516] |
| C03 | GO:0019682 | glyceraldehyde-3-phosphate metabolic process | 0.014089 | 3 | 33.33333 | [LOC4326153, LOC4329911, LOC4330320] |
| C03 | GO:0009240 | isopentenyl diphosphate biosynthetic process | 0.030179 | 3 | 25 | [LOC4326153, LOC4329911, LOC4330320] |
| C03 | GO:0042454 | ribonucleoside catabolic process | 0.000379 | 3 | 100 | [LOC4325937, LOC4333153, LOC4344179] |
| C03 | GO:0043043 | peptide biosynthetic process | 0.000206 | 42 | 6.752411 | [LOC4325462, LOC4326022, LOC4326392, LOC4326469, LOC4328392, LOC4328472, LOC4328671, LOC4328797, LOC4328826, LOC4329423, LOC4330963, LOC4332773, LOC4332813, LOC4333083, LOC4333306, LOC4333588, LOC4334512, LOC4334667, LOC4335531, LOC4335547, LOC4336196, LOC4336317, LOC4337585, LOC4338152, LOC4338199, LOC4338485, LOC4338905, LOC4338984, LOC4339145, LOC4339634, LOC4342953, LOC4346021, LOC4346044, LOC4346540, LOC4347488, LOC4348523, LOC4348562, LOC4348852, LOC4349032, LOC4350516, LOC4351596, LOC4352560] |
| C03 | GO:0034470 | ncRNA processing | 7.14E-05 | 28 | 8.668731 | [LOC4324901, LOC4325340, LOC4328064, LOC4328392, LOC4329423, LOC4329627, LOC4330282, LOC4332779, LOC4332813, LOC4334529, LOC4334688, LOC4335547, LOC4336421, LOC4336833, LOC4337783, LOC4338485, LOC4339582, LOC4339641, LOC4343215, LOC4343611, LOC4344189, LOC4344253, LOC4345558, LOC4345577, LOC4348215, LOC4348523, LOC4348618, LOC4350322] |
| C05 | GO:0016645 | oxidoreductase activity, acting on the CH-NH group of donors | 0.035903 | 3 | 10 | [LOC4327887, LOC4348932, LOC4349318] |
| C05 | GO:0043269 | regulation of ion transport | 0.03024 | 3 | 12 | [LOC4325272, LOC4326245, LOC4352510] |
| C05 | GO:0032984 | protein-containing complex disassembly | 0.041087 | 4 | 6.25 | [LOC4337837, LOC4345167, LOC4349142, LOC4350692] |
| C05 | GO:0009734 | auxin-activated signaling pathway | 0.044606 | 5 | 5.494505 | [LOC4325890, LOC4327785, LOC4329672, LOC4339130, LOC4341978] |
| C05 | GO:0009073 | aromatic amino acid family biosynthetic process | 0.029233 | 4 | 9.756098 | [LOC4335756, LOC4342026, LOC4342571, LOC4347875] |
| C05 | GO:0005216 | ion channel activity | 0.030706 | 6 | 5.825243 | [LOC107275732, LOC4325272, LOC4326245, LOC4328089, LOC4340431, LOC4352878] |
| C05 | GO:0051084 | 'de novo' posttranslational protein folding | 0.023386 | 5 | 11.90476 | [LOC4324778, LOC4327388, LOC4331379, LOC4339609, LOC4339933] |
| C05 | GO:0051085 | chaperone cofactor-dependent protein refolding | 0.023386 | 5 | 11.90476 | [LOC4324778, LOC4327388, LOC4331379, LOC4339609, LOC4339933] |
| C05 | GO:0005385 | zinc ion transmembrane transporter activity | 0.026217 | 3 | 14.28571 | [LOC4333098, LOC4333669, LOC4342783] |
| C08 | GO:0045292 | mRNA cis splicing, via spliceosome | 0.017135 | 3 | 8.333333 | [LOC4329951, LOC4330440, LOC4345884] |
| C08 | GO:0048585 | negative regulation of response to stimulus | 0.040218 | 3 | 5.357143 | [LOC4327518, LOC4347161, LOC9270058] |
| C08 | GO:0003755 | peptidyl-prolyl cis-trans isomerase activity | 0.046759 | 3 | 4.918033 | [LOC4328121, LOC4333865, LOC4342342] |
| C08 | GO:0034599 | cellular response to oxidative stress | 0.012355 | 4 | 8.888889 | [LOC4332846, LOC4347161, LOC4349382, LOC4351664] |
| C08 | GO:0004521 | endoribonuclease activity | 0.045686 | 3 | 4.83871 | [LOC4338371, LOC4349811, LOC9269203] |
| C08 | GO:0004675 | transmembrane receptor protein serine/threonine kinase activity | 0.046759 | 3 | 4.918033 | [LOC107277651, LOC4326435, LOC4344557] |
| C08 | GO:0006403 | RNA localization | 0.021118 | 4 | 5.194805 | [LOC4326176, LOC4328083, LOC4337459, LOC4344017] |
| C08 | GO:0006913 | nucleocytoplasmic transport | 0.010007 | 5 | 5.952381 | [LOC4325860, LOC4326176, LOC4328083, LOC4337459, LOC4344017] |
| C08 | GO:0006405 | RNA export from nucleus | 0.011786 | 4 | 7.843137 | [LOC4326176, LOC4328083, LOC4337459, LOC4344017] |
| C10 | GO:0009699 | phenylpropanoid biosynthetic process | 0.021471 | 3 | 6.818182 | [LOC4346217, LOC4346301, LOC4349097] |
| C10 | GO:0071396 | cellular response to lipid | 0.016412 | 5 | 4.424779 | [LOC4341108, LOC4347257, LOC4349455, LOC4352487, LOC4352488] |
| C10 | GO:0071669 | plant-type cell wall organization or biogenesis | 0.007511 | 6 | 4.918033 | [LOC107276948, LOC4330706, LOC4331665, LOC4337845, LOC4337935, LOC4339106] |
| C10 | GO:0003743 | translation initiation factor activity | 0.017694 | 4 | 5.555555 | [LOC4326910, LOC4334895, LOC4342306, LOC4343445] |
| C10 | GO:0015980 | energy derivation by oxidation of organic compounds | 0.001797 | 7 | 5.932203 | [LOC4325317, LOC4330016, LOC4334266, LOC4334274, LOC4334506, LOC4335816, LOC9268154] |
| C10 | GO:0042773 | ATP synthesis coupled electron transport | 0.017393 | 3 | 7.692307 | [LOC4325317, LOC4334266, LOC4334506] |
| C10 | GO:0006260 | DNA replication | 0.009409 | 6 | 4.615385 | [LOC4339099, LOC4340258, LOC4343985, LOC4348571, LOC4350526, LOC4352514] |
| C10 | GO:0065004 | protein-DNA complex assembly | 0.022604 | 4 | 4.819277 | [LOC4339099, LOC4348571, LOC4350526, LOC4352514] |
| C10 | GO:0032508 | DNA duplex unwinding | 0.024707 | 3 | 6 | [LOC4339099, LOC4350526, LOC4352514] |
| C11 | GO:0016679 | oxidoreductase activity, acting on diphenols and related substances as donors | 0.005772 | 3 | 7.5 | [LOC4330432, LOC4332400, LOC4343570] |
| C11 | GO:0051156 | glucose 6-phosphate metabolic process | 0.005024 | 3 | 10 | [LOC4326547, LOC4331761, LOC4347311] |
| C11 | GO:1990542 | mitochondrial transmembrane transport | 0.010343 | 3 | 5.263158 | [LOC4343123, LOC4349038, LOC4349475] |
| C13 | GO:1903311 | regulation of mRNA metabolic process | 0.016313 | 3 | 4.285714 | [LOC4332947, LOC4336934, LOC4339126] |
| C13 | GO:0044786 | cell cycle DNA replication | 0.00507 | 3 | 8.823529 | [LOC4335111, LOC4351377, LOC4352823] |
| C13 | GO:0015103 | inorganic anion transmembrane transporter activity | 0.013746 | 3 | 5.172414 | [LOC4327665, LOC4330553, LOC4346653] |
| C13 | GO:0005244 | voltage-gated ion channel activity | 0.006972 | 3 | 9.090909 | [LOC4327665, LOC4330553, LOC4341189] |
| C14 | GO:0034404 | nucleobase-containing small molecule biosynthetic process | 0.005179 | 3 | 7.692307 | [LOC4340378, LOC4345281, LOC4349403] |
| C14 | GO:0006414 | translational elongation | 0.006754 | 3 | 6.818182 | [LOC4339539, LOC4341969, LOC4344516] |
| C14 | GO:0042273 | ribosomal large subunit biogenesis | 0.008339 | 4 | 4.255319 | [LOC4330579, LOC4332209, LOC4336307, LOC4344241] |
| C14 | GO:0000278 | mitotic cell cycle | 6.22E-05 | 9 | 4.761905 | [LOC4324288, LOC4326914, LOC4327302, LOC4333851, LOC4336670, LOC4343243, LOC4349656, LOC4352280, LOC4352642] |
| C14 | GO:0001932 | regulation of protein phosphorylation | 8.17E-06 | 8 | 9.756098 | [LOC4324288, LOC4326914, LOC4331332, LOC4336670, LOC4339539, LOC4344516, LOC4352280, LOC4352642] |
| C14 | GO:0071900 | regulation of protein serine/threonine kinase activity | 2.91E-05 | 6 | 10.16949 | [LOC4324288, LOC4326914, LOC4331332, LOC4336670, LOC4352280, LOC4352642] |
| C14 | GO:1905784 | regulation of anaphase-promoting complex-dependent catabolic process | 2.19E-05 | 3 | 60 | [LOC4326397, LOC4330430, LOC4336870] |
| C14 | GO:1903364 | positive regulation of cellular protein catabolic process | 0.00358 | 3 | 9.090909 | [LOC4326397, LOC4330430, LOC4336870] |
| C14 | GO:1990757 | ubiquitin ligase activator activity | 2.19E-05 | 3 | 60 | [LOC4326397, LOC4330430, LOC4336870] |
| C15 | GO:0000027 | ribosomal large subunit assembly | 0.004953 | 3 | 7.5 | [LOC4327483, LOC4330425, LOC4350098] |
| C15 | GO:0005977 | glycogen metabolic process | 0.001428 | 3 | 18.75 | [LOC4324810, LOC4342819, LOC4343753] |
| C15 | GO:0009414 | response to water deprivation | 0.014014 | 3 | 4.411765 | [LOC4325061, LOC4334748, LOC4339745] |
| C15 | GO:0009751 | response to salicylic acid | 0.002539 | 3 | 13.63636 | [LOC4332449, LOC4347069, LOC4350434] |
| C15 | GO:0009753 | response to jasmonic acid | 0.002806 | 4 | 10 | [LOC4332449, LOC4344608, LOC4348531, LOC4350434] |
| C15 | GO:1903825 | organic acid transmembrane transport | 0.011228 | 3 | 4.918033 | [LOC4327515, LOC4346901, LOC4350398] |
| C15 | GO:0046942 | carboxylic acid transport | 0.006811 | 3 | 6.25 | [LOC4333862, LOC4346901, LOC4350398] |
| C17 | GO:0004742 | dihydrolipoyllysine-residue acetyltransferase activity | 7.38E-06 | 3 | 42.85714 | [LOC4328010, LOC4343003, LOC4347022] |
| C19 | GO:0005319 | lipid transporter activity | 0.006384 | 3 | 5.454545 | [LOC107276243, LOC4329168, LOC4344687] |
| C19 | GO:0009225 | nucleotide-sugar metabolic process | 0.006145 | 3 | 8.108109 | [LOC4333393, LOC4343849, LOC9267465] |
| C19 | GO:0006757 | ATP generation from ADP | 0.01027 | 3 | 4.347826 | [LOC4327400, LOC4330512, LOC4338737] |
| C19 | GO:0072522 | purine-containing compound biosynthetic process | 0.004176 | 4 | 4.444445 | [LOC4328470, LOC4332642, LOC4334952, LOC4345839] |
| C19 | GO:0015986 | ATP synthesis coupled proton transport | 0.008023 | 3 | 9.375 | [LOC4328470, LOC4334952, LOC4345839] |
| C23 | GO:0006888 | endoplasmic reticulum to Golgi vesicle-mediated transport | 0.000868 | 4 | 4.878049 | [LOC4327685, LOC4327708, LOC4335786, LOC4347521] |
| C27 | GO:0007346 | regulation of mitotic cell cycle | 0.000672 | 3 | 5.084746 | [LOC4325174, LOC4331610, LOC4346016] |
| C28 | GO:0009199 | ribonucleoside triphosphate metabolic process | 0.00028 | 3 | 6.122449 | [LOC4324151, LOC4339815, LOC4345078] |
| C28 | GO:0009206 | purine ribonucleoside triphosphate biosynthetic process | 0.000423 | 3 | 7.692307 | [LOC4324151, LOC4339815, LOC4345078] |
| C33 | GO:0042273 | ribosomal large subunit biogenesis | 4.82E-06 | 6 | 6.382979 | [LOC4329831, LOC4329832, LOC4336914, LOC4341262, LOC4347787, LOC4351604] |
| C36 | GO:0042273 | ribosomal large subunit biogenesis | 0.000105 | 4 | 4.255319 | [LOC4327087, LOC4336397, LOC4340741, LOC9266027] |
| C40 | GO:0009308 | amine metabolic process | 0.000322 | 3 | 4.109589 | [LOC107275892, LOC4344361, LOC4347769] |

**Table S7.** Enriched biological processes for identified gene sets from the hierarchical clustering of DEGs based on the fold-change values. See figure 4.

| **Cluster** | **GO term** | **Description** | **Annotated number in cluster** | **Annotated number in background** | **FDR** |
| --- | --- | --- | --- | --- | --- |
| C1 | GO:0032502 | developmental process | 15/1257 | 28/24075 | 0.00000066 |
| C1 | GO:0007275 | multicellular organismal development | 15/1257 | 27/24075 | 0.00000066 |
| C1 | GO:0007242 | intracellular signaling cascade | 22/1257 | 132/24075 | 0.0034 |
| C1 | GO:0016192 | vesicle-mediated transport | 26/1257 | 186/24075 | 0.0066 |
| C1 | GO:0008104 | protein localization | 34/1257 | 307/24075 | 0.0097 |
| C1 | GO:0007165 | signal transduction | 29/1257 | 240/24075 | 0.0097 |
| C1 | GO:0045184 | establishment of protein localization | 33/1257 | 294/24075 | 0.0097 |
| C1 | GO:0015031 | protein transport | 33/1257 | 294/24075 | 0.0097 |
| C1 | GO:0032535 | regulation of cellular component size | 5/1257 | 7/24075 | 0.014 |
| C1 | GO:0000226 | microtubule cytoskeleton organization | 5/1257 | 7/24075 | 0.014 |
| C1 | GO:0090066 | regulation of anatomical structure size | 5/1257 | 7/24075 | 0.014 |
| C1 | GO:0010646 | regulation of cell communication | 10/1257 | 47/24075 | 0.026 |
| C1 | GO:0009628 | response to abiotic stimulus | 13/1257 | 74/24075 | 0.026 |
| C1 | GO:0051641 | cellular localization | 31/1257 | 309/24075 | 0.049 |
| C2 | GO:0032502 | developmental process | 20/1761 | 28/24075 | 0.000000019 |
| C2 | GO:0007275 | multicellular organismal development | 19/1761 | 27/24075 | 0.000000037 |
| C2 | GO:0008104 | protein localization | 55/1761 | 307/24075 | 0.000006 |
| C2 | GO:0045184 | establishment of protein localization | 53/1761 | 294/24075 | 0.000006 |
| C2 | GO:0009725 | response to hormone stimulus | 16/1761 | 30/24075 | 0.000006 |
| C2 | GO:0015031 | protein transport | 53/1761 | 294/24075 | 0.000006 |
| C2 | GO:0007242 | intracellular signaling cascade | 32/1761 | 132/24075 | 0.000011 |
| C2 | GO:0007165 | signal transduction | 41/1761 | 240/24075 | 0.00056 |
| C2 | GO:0033036 | macromolecule localization | 59/1761 | 411/24075 | 0.00066 |
| C2 | GO:0023046 | signaling process | 44/1761 | 279/24075 | 0.0012 |
| C2 | GO:0046907 | intracellular transport | 37/1761 | 233/24075 | 0.0045 |
| C2 | GO:0016192 | vesicle-mediated transport | 31/1761 | 186/24075 | 0.0079 |
| C2 | GO:0034613 | cellular protein localization | 33/1761 | 207/24075 | 0.0089 |
| C2 | GO:0009628 | response to abiotic stimulus | 17/1761 | 74/24075 | 0.011 |
| C2 | GO:0006886 | intracellular protein transport | 32/1761 | 202/24075 | 0.011 |
| C2 | GO:0051649 | establishment of localization in cell | 42/1761 | 299/24075 | 0.012 |
| C2 | GO:0006721 | terpenoid metabolic process | 7/1761 | 13/24075 | 0.017 |
| C2 | GO:0006605 | protein targeting | 10/1761 | 32/24075 | 0.028 |
| C2 | GO:0043436 | oxoacid metabolic process | 56/1761 | 473/24075 | 0.041 |
| C2 | GO:0032535 | regulation of cellular component size | 5/1761 | 7/24075 | 0.041 |
| C2 | GO:0090066 | regulation of anatomical structure size | 5/1761 | 7/24075 | 0.041 |
| C2 | GO:0033365 | protein localization in organelle | 9/1761 | 28/24075 | 0.041 |
| C2 | GO:0019752 | carboxylic acid metabolic process | 56/1761 | 473/24075 | 0.041 |
| C3 | GO:0032502 | developmental process | 11/628 | 28/24075 | 0.00000098 |
| C3 | GO:0009725 | response to hormone stimulus | 7/628 | 30/24075 | 0.005 |
| C3 | GO:0009628 | response to abiotic stimulus | 9/628 | 74/24075 | 0.024 |
| C3 | GO:0007242 | intracellular signaling cascade | 12/628 | 132/24075 | 0.027 |
| C4 | GO:0009755 | hormone-mediated signaling pathway | 3/532 | 3/24075 | 0.038 |
| C5 | GO:0006091 | generation of precursor metabolites and energy | 18/495 | 243/24075 | 0.0058 |
| C5 | GO:0022900 | electron transport chain | 8/495 | 60/24075 | 0.015 |
| C5 | GO:0044786 | cell cycle | 3/495 | 3/24075 | 0.019 |
| C5 | GO:0030163 | protein catabolic process | 13/495 | 184/24075 | 0.034 |
| C9 | GO:0006970 | response to osmotic stress | 3/272 | 12/24075 | 0.046 |
| C9 | GO:0009725 | response to hormone stimulus | 4/272 | 30/24075 | 0.046 |
| C10 | GO:0006412 | translation | 54/735 | 595/24075 | 8.4E-09 |
| C10 | GO:0070887 | cellular response to chemical stimulus | 5/735 | 6/24075 | 0.0025 |
| C10 | GO:0042254 | ribosome biogenesis | 8/735 | 47/24075 | 0.024 |
| C10 | GO:0007275 | multicellular organismal development | 6/735 | 27/24075 | 0.039 |
| C11 | GO:0032502 | developmental process | 12/567 | 28/24075 | 0.000000019 |
| C11 | GO:0009415 | response to water | 6/567 | 14/24075 | 0.00052 |
| C11 | GO:0009628 | response to abiotic stimulus | 11/567 | 74/24075 | 0.00052 |
| C11 | GO:0032501 | multicellular organismal process | 14/567 | 154/24075 | 0.0025 |
| C11 | GO:0000272 | polysaccharide catabolic process | 6/567 | 37/24075 | 0.026 |

**Table S10.** Summary of QTL studies included in the meta-analysis for 2 populations, IR64 × Azucena populations and Azucena × other rice genotypes populations.

| Cross | Population size | Genotyping  method | Population type | References |
| --- | --- | --- | --- | --- |
| **IR64 × Azucena populations:** |  |  |  |  |
| IR64 × Azucena | 260 | SSR | DH | Venuprasad et al. (2002) |
| IR64 × Azucena | 109 | SSR | DH | Zheng et al. (2006) |
| IR64 × Azucena | 96 | SNP and SSR | DH | Zheng et al. (2008) |
| IR64 × Azucena | 248 | EST | DH | Zheng et al. (2006) |
| IR64 × Azucena | 109 | RFLP, AFLP | DH | Zheng et al. (1998) |
| IR64 × Azucena | 135 | RFLP | DH | Hemamalini et al. (2000) |
| IR64 × Azucena | 260 | molecular marker-assisted QTL tagging | DH | Venuprasad et al. (2002) |
| IR64 × Azucena | 312 | RFLP | NILs, DH | Shen et al. (2001) |
| **Azucena × other rice genotypes populations:** | | |  |  |
| Bala×Azucena | 205 | AFLP and RFLP | RIL | Cairns et al. (2009) |
| Bala × Azucena | 168 | SSR | RIL | Emrich et al. (2008) |
| Bala × Azucena | 205 | SSR | RIL | Norton et al. (2009) |
| Bala × Azucena | 205 | SSRs | RIL (F6) | MacMillan et al. (2006) |
| Bala × Azucena | 205 | RFLP and SSR | RIL (F6) | Price et al. (2002) |
| KalingaIII × Azucena | 120 | SSR | NIL | Steele et al. (2013) |
| IR1552 × Azucena | 96 | ESTs, cDNA-AFLP and SSR | RIL | Zheng et al. (2003) |
| IR1552 × Azucena | 249 | EST$cDNA-AFLP | RILs F10 | Zheng et al. (2003) |
| IR1552 × Azucena |  | RFLP, AFLP | RILs F10 | Zhang et al. (2001b) |
| Bala × Azucena | 205 | RFLP, AFLP | F2, RILS | Price et al. (2000) |
| Bala × Azucena | 205 | marker-based selection | RILs, RINILs | Gareth et al. (2009) |
| Kalinga III × Azucena | 180 | SSRs | NILs | Steele et al. (2006) |

**Table S11**. List of QTLs associated with root morphological traits under well-watered and water stress conditions used for the QTL meta-analysis (For two populations: (IR64 × Azucena populations and Azucena × other rice genotypes populations). Cond. = condition, N = normal condition, S = stress condition, Chr. = chromosome, LOD= logarithm (base 10) of odds, R2= phenotypic variance explained by individual QTL (%), Posit. = Position of QTL on consensus map, Posit. + CI = Position of QTL plus confidence interval (CI), Posit. - CI = Position of QTL minus confidence interval; Traits: RL: root length, RSR: root to shoot ratio, MRL: maximum root length DRW: deep rooting weight, RDW: root dry weight, RN: root number, RV: root volume, RTHK: root thickness, DS: drought stress, DRR: deep root ratio, RSAr: root surface area, RGA: root growth angle.

| QTL name | Trait | Cond. | Year of study | Chr. number | Linkage group | LOD | R2 | Posit. | Posit.+ CI | Posit.- CI | Obtained-M-QTLs |
| --- | --- | --- | --- | --- | --- | --- | --- | --- | --- | --- | --- |
| IR64 × Azucena populations: | | | | | |  |  |  |  |  |  |
| QRN1_1 | RN | S | 2000 | Chr1 | linkage_group1 | 1.81 | 14.1 | 14.35 | 6.89005779 | 21.8099422 | MQTL1.1 |
| QRN1_1 | RN | S | 2000 | Chr1 | linkage_group1 | 2.11 | 8.8 | 21.35 | 8.42256047 | 34.2774395 | MQTL1.1 |
| QRN1_2 | RN | S | 2000 | Chr1 | linkage_group1 | 3 | 24.5 | 45.1 | 40.8067271 | 49.3932729 | MQTL1.1 |
| QRSR1_1 | RSR | S | 2000 | Chr1 | linkage_group1 | 1.63 | 12.7 | 58.45 | 50.167702 | 66.7322981 | MQTL1.2 |
| QRN1_2 | RN | N | 2006 | Chr1 | linkage_group1 | 2.98 | 13.3 | 101.55 | 97.7906015 | 105.309399 | MQTL1.2 |
| QRDW1_3 | RDW | N | 2006 | Chr1 | linkage_group1 | 2.47 | 11.3 | 120.2 | 115.775221 | 124.624779 | MQTL1.2 |
| QRL1_1 | RL | S | 2000 | Chr1 | linkage_group1 | 1.64 | 13.7 | 122.6 | 114.922249 | 130.277751 | MQTL1.2 |
| QRN1_1 | RN | N | 2006 | Chr1 | linkage_group1 | 2.59 | 11.7 | 127.6 | 123.326496 | 131.873504 | MQTL1.2 |
| QMRL1_1 | MRL | S | 2001 | Chr1 | linkage_group1 | 3 | 8.9 | 129 | 120.1 | 137.9 | MQTL1.2 |
| QDRW1_2 | DRW | S | 2001 | Chr1 | linkage_group1 | 3 | 9.6 | 129 | 119.4 | 138.6 | MQTL1.2 |
| QDRW1_1 | DRW | S | 2001 | Chr1 | linkage_group1 | 3 | 7.5 | 136.45 | 128.95 | 143.95 | MQTL1.3 |
| QRL1_1 | RL | N | 2088 | Chr1 | linkage_group1 | 5.03 | 21.6 | 142.1 | 135.18 | 149.02 | MQTL1.3 |
| QDRR1_1 | DRR | S | 2000 | Chr1 | linkage_group1 | 2.61 | 16.4 | 151 | 144.063325 | 157.936675 | MQTL1.3 |
| QRL1_2 | RL | N | 2088 | Chr1 | linkage_group1 | 2.57 | 11.1 | 162.8 | 149.33 | 176.27 | MQTL1.3 |
| QRL1_1 | RL | N | 2006 | Chr1 | linkage_group1 | 2.6 | 12 | 167.35 | 163.183333 | 171.516667 | MQTL1.3 |
| QRDW1_2 | RDW | N | 2006 | Chr1 | linkage_group1 | 3.49 | 15.6 | 167.35 | 164.144872 | 170.555128 | MQTL1.3 |
| QRL1_2 | RL | N | 2006 | Chr1 | linkage_group1 | 2.42 | 11.8 | 215.4 | 211.162712 | 219.637288 | MQTL1.3 |
| QRDW1_1 | RDW | N | 2006 | Chr1 | linkage_group1 | 3.55 | 15.7 | 215.4 | 212.215287 | 218.584713 | MQTL1.3 |
| QDRR2_1 | DRR | S | 2000 | Chr2 | linkage_group2 | 2 | 8.4 | 47.75 | 34.2069681 | 61.2930319 | MQT2.1 |
| QMRL2_1 | MRL | S | 2001 | Chr2 | linkage_group2 | 3 | 9.9 | 66.7 | 56.8 | 76.6 | MQT2.1 |
| QRDW2_1 | RDW | S | 2000 | Chr2 | linkage_group2 | 1.58 | 13.3 | 67.75 | 59.8413395 | 75.6586605 | MQT2.1 |
| QRL2_1 | RL | S | 2000 | Chr2 | linkage_group2 | 1.62 | 15.4 | 67.75 | 60.9197932 | 74.5802068 | MQT2.1 |
| QRV2_1 | RV | S | 2000 | Chr2 | linkage_group2 | 1.74 | 15.6 | 67.75 | 61.0073599 | 74.4926401 | MQT2.1 |
| QRN2_1 | RN | S | 2000 | Chr2 | linkage_group2 | 1.8 | 17.4 | 67.75 | 61.7048744 | 73.7951256 | MQT2.1 |
| QRSR2_3 | RSR | N | 2088 | Chr2 | linkage_group2 | 2.73 | 9.2 | 92.4 | 76.15 | 108.65 | MQT2.2 |
| QRTHK2_1 | RTHK | S | 2000 | Chr2 | linkage_group2 | 1.66 | 16.7 | 86.3 | 80.0014859 | 92.5985141 | MQT2.2 |
| QRTHK2_1 | RTT | S | 2002 | Chr2 | linkage_group2 | 3.21 | 26.9 | 86.3 | 82.1535888 | 90.4464112 | MQT2.2 |
| QRTHK2_1 | RTHK | S | 2000 | Chr2 | linkage_group2 | 3.21 | 26.7 | 86.3 | 82.36048 | 90.23952 | MQT2.2 |
| QRTHK2_2 | RTHK | S | 2000 | Chr2 | linkage_group2 | 1.59 | 12.4 | 123.25 | 114.767324 | 131.732676 | MQT2.3 |
| QRL2_1 | RL | N | 2006 | Chr2 | linkage_group2 | 2.46 | 11.3 | 122.4 | 117.975221 | 126.824779 | MQT2.3 |
| QRN2_2 | RN | S | 2000 | Chr2 | linkage_group2 | 2.69 | 25.1 | 123.25 | 119.059355 | 127.440645 | MQT2.3 |
| QRSR2_4 | RSR | N | 2088 | Chr2 | linkage_group2 | 2.46 | 8 | 153.2 | 134.52 | 155.88 | MQT2.4 |
| QRSR2_1 | RSR | S | 2000 | Chr2 | linkage_group2 | 2.79 | 13.5 | 159.15 | 150.723225 | 167.576775 | MQT2.4 |
| QRN2_3 | RN | S | 2000 | Chr2 | linkage_group2 | 2.26 | 17.7 | 159.15 | 153.207334 | 165.092666 | MQT2.4 |
| QRSR3_1 | RSR | S | 2000 | Chr3 | linkage_group3 | 2.18 | 8.9 | 6 | -6.7821874 | 18.7821874 | MQTL3.1 |
| QDRR3_1 | DRR | S | 2000 | Chr3 | linkage_group3 | 2.33 | 10.1 | 6 | -5.2635117 | 17.2635117 | MQTL3.1 |
| QRDW3_1 | RDW | S | 2000 | Chr3 | linkage_group3 | 2.8 | 20.8 | 6 | 0.94301994 | 11.0569801 | MQTL3.1 |
| QRV3_1 | RV | S | 2000 | Chr3 | linkage_group3 | 2.89 | 21.4 | 6 | 1.08480443 | 10.9151956 | MQTL3.1 |
| QRV3_1 | RTV | S | 2002 | Chr3 | linkage_group3 | 4.02 | 29.1 | 15.2 | 11.3670632 | 19.0329368 | MQTL3.2 |
| QRDW3_1 | RDW | S | 2002 | Chr3 | linkage_group3 | 4.22 | 30.7 | 15.2 | 11.5668254 | 18.8331746 | MQTL3.2 |
| QRL3_5 | RL | N | 2088 | Chr3 | linkage_group3 | 2.61 | 13 | 58.85 | 47.35 | 70.35 | MQTL3.3 |
| QRDW3_1 | RDW | N | 2006 | Chr3 | linkage_group3 | 3.12 | 13.9 | 67.15 | 63.5528777 | 70.7471223 | MQTL3.4 |
| QRL3_6 | RL | N | 2088 | Chr3 | linkage_group3 | 3.17 | 14.1 | 87.08 | 76.47 | 97.68 | MQTL3.4 |
| QRDW4_1 | RDW | N | 2006 | Chr4 | linkage_group4 | 3.01 | 13.4 | 6.65 | 2.91865672 | 10.3813433 | MQTL4.1 |
| QRN4_1 | RN | S | 2000 | Chr4 | linkage_group4 | 1.54 | 11.9 | 24.1 | 15.2609088 | 32.9390912 | MQTL4.2 |
| QRSR4_7 | RSR | N | 2088 | Chr4 | linkage_group4 | 3.06 | 12.5 | 85.05 | 73.09 | 97.01 | MQTL4.3 |
| QDS4_1 | DS | S | 2000 | Chr4 | linkage_group4 | 1.8 | 14.4 | 91 | 83.6954733 | 98.3045268 | MQTL4.3 |
| QDRR4_1 | DRR | S | 2000 | Chr4 | linkage_group4 | 2.3 | 14 | 104.65 | 96.5241809 | 112.775819 | MQTL4.4 |
| QRL4_2 | RL | S | 2000 | Chr4 | linkage_group4 | 2.07 | 16 | 104.65 | 98.0759259 | 111.224074 | MQTL4.4 |
| QRL4_1 | RL | S | 2000 | Chr4 | linkage_group4 | 1.65 | 12.7 | 120.5 | 112.217702 | 128.782298 | MQTL4.4 |
| QRTHK5_1 | RTHK | S | 2000 | Chr5 | linkage_group5 | 2.41 | 19.1 | 27.6 | 22.0929222 | 33.1070778 | MQTL5.1 |
| QRL5_1 | RL | N | 2006 | Chr5 | linkage_group5 | 3.65 | 16.1 | 27.6 | 24.4944099 | 30.7055901 | MQTL5.1 |
| QRV5_1 | RV | S | 2000 | Chr5 | linkage_group5 | 1.6 | 13.5 | 43.05 | 35.2585048 | 50.8414952 | MQTL5.2 |
| QRSR5_9 | RSR | N | 2088 | Chr5 | linkage_group5 | 3.06 | 11.6 | 103.6 | 90.71 | 116.49 | MQTL5.3 |
| QRL5_8 | RL | N | 2088 | Chr5 | linkage_group5 | 3.05 | 13.7 | 103.6 | 92.69 | 114.51 | MQTL5.3 |
| QRV5_2 | RV | S | 2000 | Chr5 | linkage_group5 | 1.82 | 15.1 | 100.15 | 93.1840937 | 107.115906 | MQTL5.3 |
| QRDW5_1 | RDW | N | 2006 | Chr5 | linkage_group5 | 5.04 | 21.7 | 100.15 | 97.8458525 | 102.454148 | MQTL5.3 |
| QRN6_1 | RN | S | 2000 | Chr6 | linkage_group6 | 1.65 | 12.9 | 81.1 | 72.9461097 | 89.2538903 | MQTL6.1 |
| QRSR6_10 | RSR | N | 2088 | Chr6 | linkage_group6 | 2.4 | 11.4 | 89 | 75.89 | 102.11 | MQTL6.2 |
| QRL6_1 | RL | N | 2006 | Chr6 | linkage_group6 | 3.4 | 15 | 180.8 | 177.466667 | 184.133333 | MQTL6.2 |
| Qrn7_1 | RN | S | 2000 | Chr7 | linkage_group7 | 3.21 | 14.3 | 6 | -1.9553474 | 13.9553474 | MQTL7.1 |
| Qrn7_1 | RN | S | 2000 | Chr7 | linkage_group7 | 2.06 | 15.7 | 57.35 | 50.6503067 | 64.0496933 | MQTL7.1 |
| Qrn7_1 | RN | S | 2000 | Chr7 | linkage_group7 | 1.58 | 15.3 | 73.1 | 66.2251513 | 79.9748487 | MQTL7.2 |
| Qdrr7_1 | DRR | S | 2000 | Chr7 | linkage_group7 | 2.1 | 9 | 87.9 | 75.2598369 | 100.540163 | MQTL7.3 |
| Qrv7_1 | RV | S | 2000 | Chr7 | linkage_group7 | 1.74 | 14.3 | 87.9 | 80.5443926 | 95.2556074 | MQTL7.3 |
| Qmrl7_1 | MRL | S | 2001 | Chr7 | linkage_group7 | 3 | 17.7 | 101.9 | 84.2 | 119.6 | MQTL7.3 |
| Qmrl7_1 | DRW | S | 2001 | Chr7 | linkage_group7 | 3 | 14.7 | 101.9 | 87.2 | 116.6 | MQTL7.3 |
| Qrsr7_1 | RSR | S | 2000 | Chr7 | linkage_group7 | 2.52 | 11.7 | 105.25 | 95.5267976 | 114.973202 | MQTL7.4 |
| Qmrl7_2 | DRW | S | 2001 | Chr7 | linkage_group7 | 3 | 4.8 | 101.9 | 97.1 | 106.7 | MQTL7.4 |
| Qrsr8_1 | RSR | S | 2000 | Chr8 | linkage_group8 | 2.06 | 13 | 10.1 | 1.34911786 | 18.8508822 | MQTL8.1 |
| Qrn8_1 | RN | N | 2006 | Chr8 | linkage_group8 | 2.42 | 11 | 7.15 | 2.60454546 | 11.6954546 | MQTL8.1 |
| Qrn8_2 | RN | N | 2006 | Chr8 | linkage_group8 | 2.41 | 11 | 58.85 | 54.3045455 | 63.3954546 | MQTL8.2 |
| Qrl8_1 | RL | N | 2006 | Chr8 | linkage_group8 | 2.54 | 11.5 | 98.1 | 93.7521739 | 102.447826 | MQTL8.3 |
| Qrthk8_1 | RTHK | S | 2000 | Chr8 | linkage_group8 | 2.94 | 21.6 | 149.7 | 144.830316 | 154.569685 | MQTL8.4 |
| Qrthk9_1 | RTHK | S | 2000 | Chr9 | linkage_group9 | 1.62 | 12.7 | 35.9 | 27.617702 | 44.1822981 | MQTL9.1 |
| Qrl9_1 | RL | S | 2000 | Chr9 | linkage_group9 | 1.63 | 14.7 | 50.45 | 43.2945452 | 57.6054548 | MQTL9.1 |
| Qmrl9_2 | DRW | S | 2001 | Chr9 | linkage_group9 | 3 | 5.6 | 49.65 | 44.05 | 55.25 | MQTL9.2 |
| Qdrr9_1 | DRR | S | 2000 | Chr9 | linkage_group9 | 2.25 | 10.5 | 66.3 | 55.4655745 | 77.1344255 | MQTL9.3 |
| Qmrl9_1 | MRL | S | 2001 | Chr9 | linkage_group9 | 3 | 8.8 | 76.75 | 67.95 | 85.55 | MQTL9.4 |
| Qmrl9_1 | RTL | S | 2002 | Chr9 | linkage_group9 | 3.63 | 12.9 | 77.05 | 68.4036076 | 85.6963924 | MQTL9.4 |
| Qrn10_1 | RN | S | 2000 | Chr10 | linkage_group10 | 1.75 | 13.4 | 69.8 | 61.9503593 | 77.6496407 | MQTL10.1 |
| Qds12_1 | DRW | S | 2000 | Chr12 | linkage_group12 | 2.03 | 16.1 | 107.1 | 100.566759 | 113.633241 | MQTL12.1 |
| Azucena × Other Rice Genotypes Populations: | | | | | | |  |  |  |  |  |
| Qrn1_2 | RN | S | 2000 | Chr1 | linkage_group1 | 3 | 5.8 | 49 | 43.2 | 54.8 | MQTL1.1 |
| Qrn1_1 | RN | S | 2000 | Chr1 | linkage_group1 | 3 | 12.4 | 61.3 | 48.9 | 73.7 | MQTL1.2 |
| Qrn1_2 | RN | N | 2003 | Chr1 | linkage_group1 | 2.71 | 13.4 | 100.15 | 97.7073938 | 102.59261 | MQTL1.2 |
| Qrl1_1 | RL | N | 2003 | Chr1 | linkage_group1 | 2.43 | 11.8 | 107.6 | 104.826193 | 110.37381 | MQTL1.2 |
| Qrn1_1 | RN | N | 2003 | Chr1 | linkage_group1 | 2.41 | 12 | 166.15 | 163.422423 | 168.87758 | MQTL1.3 |
| Qrl1_1 | RL | N | 2003 | Chr1 | linkage_group1 | 2.5 | 11.3 | 174.75 | 171.853458 | 177.64654 | MQTL1.3 |
| Qrn1_3 | RN | S | 2000 | Chr1 | linkage_group1 | 3 | 10.3 | 176.3 | 166 | 186.6 | MQTL1.3 |
| Qrl2_1 | RL | N | 2003 | Chr2 | linkage_group2 | 2.57 | 12 | 12.3 | 9.57242303 | 15.027577 | MQTL2.1 |
| Qrl2_1 | RL | N | 2009 | Chr2 | linkage_group2 | 3.9 | 19.3 | 84.65 | 82.5900986 | 86.709901 | MQTL2.1 |
| Qdrr2_1 | DRR | S | 2000 | Chr2 | linkage_group2 | 3 | 8.6 | 89.9 | 81.3 | 98.5 | MQTL2.1 |
| Qrn2_1 | RN | S | 2000 | Chr2 | linkage_group2 | 3 | 9.9 | 89.9 | 80 | 99.8 | MQTL2.1 |
| Qrl2_1 | RN | N | 2003 | Chr2 | linkage_group2 | 3.22 | 18.2 | 92.9 | 91.1015976 | 94.698402 | MQTL2.1 |
| Qdrr2_1 | DRR | S | 2006 | Chr2 | linkage_group2 | 3 | 18 | 112.45 | 109.934568 | 114.96543 | MQTL2.2 |
| Qrsr2_1 | RSR | S | 2006 | Chr2 | linkage_group2 | 3 | 10.8 | 121.85 | 117.657613 | 126.04239 | MQTL2.2 |
| Qdrr2_2 | DRR | S | 2000 | Chr2 | linkage_group2 | 3 | 16.7 | 128.3 | 111.6 | 145 | MQTL2.2 |
| Qrn2_2 | RN | S | 2000 | Chr2 | linkage_group2 | 3 | 18 | 128.3 | 110.3 | 146.3 | MQTL2.2 |
| Qdrr2_2 | DRR | N | 2006 | Chr2 | linkage_group2 | 3 | 15.8 | 135.5 | 132.634318 | 138.36568 | MQTL2.2 |
| Qrl2_2 | RL | N | 2009 | Chr2 | linkage_group2 | 2.7 | 10 | 145.35 | 141.37439 | 149.32561 | MQTL2.2 |
| Qtrhk2_1 | TRHK | N | 2006 | Chr2 | linkage_group2 | 3 | 4.8 | 183.1 | 173.66713 | 192.53287 | MQTL2.2 |
| Qrl3_3 | RN | N | 2003 | Chr3 | linkage_group3 | 2.46 | 11.4 | 0.55 | -2.32113366 | 3.4211337 | MQTL3.1 |
| Qrl3_1 | RL | N | 2009 | Chr3 | linkage_group3 | 3.2 | 9.3 | 10.35 | 6.0751508 | 14.624849 | MQTL3.2 |
| Qrl3_1 | RN | N | 2003 | Chr3 | linkage_group3 | 2.48 | 11.7 | 13.8 | 11.0024852 | 16.597515 | MQTL3.2 |
| Qrl3_1 | RN | N | 2003 | Chr3 | linkage_group3 | 2.72 | 15 | 52.4 | 50.2179384 | 54.582062 | MQTL3.3 |
| Qrl3_1 | RL | N | 2003 | Chr3 | linkage_group3 | 2.94 | 13.4 | 53.15 | 50.7073938 | 55.592606 | MQTL3.3 |
| Qrl3_2 | RN | N | 2003 | Chr3 | linkage_group3 | 3.01 | 13.7 | 149.35 | 146.960882 | 151.73912 | MQTL3.3 |
| Qrl4_1 | RN | N | 2003 | Chr4 | linkage_group4 | 3.08 | 14 | 100.4 | 98.0620769 | 102.73792 | MQTL4.1 |
| Qrl4_1 | RN | N | 2003 | Chr4 | linkage_group4 | 2.48 | 12.3 | 69.6 | 66.9389493 | 72.261051 | MQTL4.1 |
| Q5_1 | RN | S | 2000 | Chr5 | linkage_group5 | 3 | 6.8 | 87.4 | 80.6 | 94.2 | MQTL5.1 |
| Qrl5_1 | RL | N | 2003 | Chr5 | linkage_group5 | 3.18 | 14.4 | 78 | 75.7270192 | 80.272981 | MQTL5.1 |
| Qdrr5_1 | DRR | S | 2000 | Chr5 | linkage_group5 | 3 | 5.2 | 87.4 | 82.2 | 92.6 | MQTL5.2 |
| Qrga6_1 | RGA | N | 2009 | Chr6 | linkage_group6 | 2.8 | 8.2 | 31.8 | 26.9516954 | 36.648305 | MQTL6.1 |
| Qrl6_1 | RL | N | 2003 | Chr6 | linkage_group6 | 2.42 | 11.9 | 191.9 | 189.149502 | 194.6505 | MQTL6.1 |
| Qrl6_2 | RL | N | 2003 | Chr6 | linkage_group6 | 2.42 | 11.8 | 293.4 | 290.626193 | 296.17381 | MQTL6.1 |
| Qrl6_1 | RN | N | 2003 | Chr6 | linkage_group6 | 2.78 | 13.4 | 115.45 | 113.007394 | 117.89261 | MQTL6.1 |
| Qdrr7_1 | DRR | S | 2006 | Chr7 | linkage_group7 | 3 | 22.3 | 83.3 | 81.2696064 | 85.330394 | MQTL7.1 |
| Qrl7_1 | RL | N | 2003 | Chr7 | linkage_group7 | 2.46 | 11.5 | 94.75 | 91.9038327 | 97.596167 | MQTL7.2 |
| Qrn7_1 | RN | S | 2006 | Chr7 | linkage_group7 | 3 | 14.7 | 101.75 | 98.6698791 | 104.83012 | MQTL7.3 |
| Qrsr7_1 | RSR | S | 2006 | Chr7 | linkage_group7 | 3 | 18.7 | 108.8 | 106.378729 | 111.22127 | MQTL7.4 |
| Qrsr8_1 | RSR | S | 2006 | Chr8 | linkage_group8 | 3 | 17.7 | 75.7 | 73.1419335 | 78.258067 | MQTL8.1 |
| Qrdr9_64 | RDR | N | 2011 | Chr9 | linkage_group9 | 26.9 | 66.6 | 60.8 | 59.75 | 61.85 | MQTL9.1 |
| Qmrl9_1 | MRL | S | 2006 | Chr9 | linkage_group9 | 3 | 69 | 63.7 | 63.0438003 | 64.3562 | MQTL9.2 |
| Qrsa9_1 | RSAr | S | 2006 | Chr9 | linkage_group9 | 3 | 18.1 | 81.15 | 78.6484653 | 83.651535 | MQTL9.3 |
| Qrl9_1 | RN | N | 2003 | Chr9 | linkage_group9 | 4.56 | 20 | 91.35 | 89.7134538 | 92.986546 | MQTL9.4 |
| Qrl9_1 | RL | N | 2003 | Chr9 | linkage_group9 | 2.99 | 13.4 | 92.8 | 90.3573938 | 95.242606 | MQTL9.4 |
| Qrga10_1 | RGA | N | 2009 | Chr10 | linkage_group10 | 3.9 | 17.7 | 56.7 | 54.4538928 | 58.946107 | MQTL10.1 |
| Qdrr11_1 | DRR | N | 2006 | Chr11 | linkage_group11 | 3 | 13 | 80.3 | 76.817094 | 83.782906 | MQTL11.1 |
| Qrl11_1 | RL | N | 2009 | Chr11 | linkage_group11 | 3.6 | 27.1 | 87.15 | 85.6829853 | 88.617015 | MQTL11.1 |
| Qdrr11_1 | DRR | S | 2000 | Chr11 | linkage_group11 | 3 | 6.9 | 87 | 80.1 | 93.9 | MQTL11.2 |
| Q11_1 | RN | S | 2000 | Chr11 | linkage_group11 | 3 | 7.4 | 87 | 79.6 | 94.4 | MQTL11.2 |
| Qdrr11_2 | DRR | S | 2006 | Chr11 | linkage_group11 | 3 | 29.8 | 101.75 | 100.230612 | 103.26939 | MQTL11.2 |

**Table S12.** List of obtained Meta-QTLs from meta-analysis of the collected QTLs associated root system architecture traits under normal and water stress conditions (For two populations: (IR64 × Azucena populations and Azucena × other rice genotypes populations). Chr.: chromosome; Posit.: position; CI: confidence interval; bp: base pair; cM: centimorgan.

| MetaQTL | Chr. number | Meta-QTL Peak Posit. (cM) | Posit. 1 (cM) | Posit. 2 (cM) | Physical Posit. (pb) | Physical Posit. 2(bp) | Flanking marker of the posit. (1) | Flanking marker of the posit. (2) | Length of Meta-QTL (cM) | Physical Length of Meta-QTL (bp) | 95% CI (cM) | Number of Genes | Number of initial QTLs | Trait |
| --- | --- | --- | --- | --- | --- | --- | --- | --- | --- | --- | --- | --- | --- | --- |
| IR64 × Azucena populations | | | | |  |  |  |  |  |  |  |  |  |  |
| M-QTL 1.1 | 1 | 43.76 | 46.065 | 41.455 | 7717298 | 8800087 | S11941A | RM8091 | 4.61 | 1082789 |  | 155 | 3 | RN |
| M-QTL 1.2 | 1 | 128.61 | 125.64 | 131.58 | 30092742 | 31947440 | RM1152 | RM3709 | 5.94 | 1854698 |  | 353 | 7 | RN, RSR, RDW, RL, MRL, DRW |
| M-QTL 1.3 | 1 | 164.39 | 161.55 | 167.24 | 40670383 | 41563269 | RM529 | R503-W858 | 5.69 | 892886 |  | 186 | 8 | RL, DRW, DRR |
| M-QTL 2.1 | 2 | 66.58 | 63.45 | 69.7 | 18529790 | 19336316 | RM4499 | RM341 | 6.25 | 806526 |  | 105 | 6 | DRR, MRL, RDW, RL, RV, RN |
| M-QTL 2.2 | 2 | 83.38 | 85.805 | 80.955 | 20445714 | 21342449 | C796A | E3634S | 4.85 | 896735 |  | 129 | 4 | RSR, RTHK, |
| M-QTL 2.3 | 2 | 123.24 | 119.49 | 127 | 28351861 | 30270605 | R3393-G57 | C601-C1408 | 7.51 | 1918744 |  | 341 | 3 | RTHK, RL, RN |
| M-QTL 2.4 | 2 | 158.13 | 153.7 | 162.55 | 35061036 | 35661689 | RM5894 | RG520 | 8.85 | 600653 |  | 172 | 3 | RSR, RN |
| M-QTL 3.1 | 3 | 6 | 2.45 | 14.45 | 2452877 | 3524119 | RM1332 | RZ329 | 12 | 1071242 |  | 225 | 4 | RSR, DRR, RDW. RV |
| M-QTL3.2 | 3 | 16.45 | 17.29 | 15.61 | 3179705 | 3931850 | RM4683 | C12266SA | 1.68 | 752145 |  | 92 | 2 | RV, RDW |
| M-QTL 3.3 | 3 | 55.37 | 58.855 | 51.885 | 11525225 | 12574837 | R3156 | RM5928 | 6.97 | 1049612 |  | 149 | 1 | RL |
| M-QTL 3.4 | 3 | 90.65 | 92.35 | 88.95 | 22391304 | 23126064 | RM5864 | RM16 | 3.4 | 734760 |  | 61 | 2 | RDW, RL |
| M-QTL 4.1 | 4 | 7.3 | 9.895 | 4.705 | 361889 | 3546753 | E20565S | Y3635R | 5.19 | 3184864 |  | 195 | 1 | RDW |
| M-QTL 4.2 | 4 | 24.1 | 15.26 | 32.93 | 5649050 | 16522331 | RZ262 | P150-E61384S | 17.67 | 10873281 |  | 554 | 1 | RN |
| M-QTL 4.3 | 4 | 89.38 | 83.14 | 95.61 | 26782001 | 29060978 | RM1018 | RM317 | 12.47 | 2278977 |  | 330 | 2 | RSR |
| M-QTL 4.4 | 4 | 110.63 | 111.27 | 109.99 | 31875539 | 32117010 | RM6909 | C55 | 1.28 | 241471 |  | 49 | 3 | DRR, RL |
| M-QTL 5.1 | 5 | 27.6 | 24.89 | 30.3 | 3073406 | 3450870 | RM405 | RM574 | 5.41 | 377464 |  | 47 | 2 | RTHK, RL |
| M-QTL 5.2 | 5 | 43.04 | 35.25 | 50.84 | 4789309 | 8304142 | C53428S | RM516 | 15.59 | 3514833 |  | 316 | 1 | RV |
| M-QTL 5.3 | 5 | 102.85 | 107.33 | 98.37 | 23445449 | 25769955 | C10405S | RM3631 | 8.96 | 2324506 |  | 361 | 4 | RL, RV,RSR, RDW |
| M-QTL 6.1 | 6 | 88.91 | 90.76 | 87.06 | 23737032 | 24491014 | RM454 | R1559 | 3.7 | 753982 |  | 98 | 1 | RN |
| M-QTL 6.2 | 6 | 96.03 | 98.95 | 93.11 | 24035491 | 25996122 | RM162 | CDO544 | 5.84 | 1960631 |  | 175 | 2 | RSR, RL |
| M-QTL 7.1 | 7 | 59.12 | 61.365 | 56.875 | 16521697 | 17907948 | R430 | C11425S | 4.49 | 1386251 |  | 76 | 2 | RN |
| M-QTL 7.2 | 7 | 87.9 | 81.54 | 94.25 | 23109505 | 25457233 | RM5623 | RM473A | 12.71 | 2347728 |  | 386 | 1 | RN |
| M-QTL 7.3 | 7 | 93.36 | 93.415 | 93.305 | 25301976 | 25457233 | C847 | RM473A | 0.11 | 155257 |  | 32 | 4 | DRR, RV, MRL, DRW |
| M-QTL 7.4 | 7 | 101.9 | 97.26 | 106.53 | 26354749 | 28411532 | RM3552 | RZ978 | 9.27 | 2056783 |  |  | 2 | RSR, DRW |
| M-QTL 8.1 | 8 | 7.77 | 3.74 | 11.81 | 682581 | 1527975 | T77-T78 | RZ143 | 8.07 | 845394 |  | 131 | 2 | RSR, RN |
| M-QTL 8.2 | 8 | 58.85 | 54.3 | 63.39 | 11983657 | 17437513 | C1374-C1115 | RG978 | 9.09 | 5453856 |  | 295 | 1 | RN |
| M-QTL 8.3 | 8 | 98.1 | 93.75 | 102.44 | 23570745 | 24941768 | RM8043 | S6487S | 8.69 | 1371023 |  | 224 | 1 | RL |
| M-QTL 8.4 | 8 | 63.69 | 65.75 | 61.63 | 18249652 | 19006530 | S3680 | RM4815 | 4.12 | 756878 |  | 65 | 1 | RTHK |
| M-QTL 9.1 | 9 | 35.9 | 27.61 | 44.18 | 9629362 | 13579197 | C1454 | RM105 | 16.57 | 3949835 |  | 168 | 2 | RTHK, RL |
| M-QTL 9.2 | 9 | 52.75 | 53.745 | 51.755 | 14692058 | 15077893 | RM6771 | S2074 | 1.99 | 385835 |  | 49 | 1 | DRW |
| M-QTL 9.3 | 9 | 60.75 | 61.58 | 59.92 | 15662573 | 16972710 | RM434 | W574 | 1.66 | 1310137 |  | 174 | 1 | DRR |
| M-QTL 9.4 | 9 | 69.49 | 70.05 | 68.93 | 18514769 | 18920684 | R1562 | RM3164 | 1.12 | 405915 |  | 80 | 2 | RTHK |
| M-QTL 10.1 | 10 | 69.8 | 61.95 | 77.64 | 20318289 | 22372009 | RM294A | RM333 | 15.69 | 2053720 |  | 373 | 1 | RN |
| M-QTL 12.1 | 12 | 107.1 | 100.56 | 113.63 | 26107904 | 27488270 | M320B | RG181 | 13.07 | 1380366 |  | 204 | 1 | DRW |
| Azucena × Other Rice Genotypes Populations: | | | | |  |  |  |  |  |  |  |  |  |  |
| M-QTL 1.1 | 1 | 43.76 | 46.065 | 41.455 | 7717298 | 8800087 | S11941A | RM8091 | 4.61 | 1082789 |  | 155 | 1 | RN |
| M-QTL 1.2 | 1 | 68.33 | 71.8 | 64.86 | 12092238 | 18976324 | C51420S | RM24 | 6.94 | 6884086 |  | 476 | 3 | RN, RL |
| M-QTL 1.3 | 1 | 170.57 | 172.91 | 168.23 | 41562935 | 42375962 | R503 | RM6407 | 4.68 | 813027 |  | 4 | 3 | RL , RN |
| M-QTL 2.1 | 2 | 83.38 | 85.805 | 80.955 | 20445714 | 21342449 | C796A | E3634S | 4.85 | 896735 |  | 129 | 5 | RL, DRR, RN |
| M-QTL 2.2 | 2 | 112.37 | 114.185 | 110.555 | 26965236 | 27609877 | RM6366 | RM221 | 3.63 | 644641 |  | 112 | 7 | DRR, RSR, RN, RL, TRHK |
| M-QTL 3.1 | 3 | 0.55 | 2.32 | 3.42 | 105852 | 1116926 | RM60 | RM3894 | 1.1 | 1011074 |  | 220 | 1 | RN |
| M-QTL3.2 | 3 | 10.34 | 6 | 14.62 | 1396188 | 3120806 | RM4853 | RM5628 | 8.62 | 1724618 |  | 356 | 2 | RL, RN |
| M-QTL 3.3 | 3 | 148.865 | 146 | 151.73 | 32923195 | 33920501 | S759 | C1141 | 5.73 | 997306 |  | 217 | 3 | RN, RL, RN |
| M-QTL 4.1 | 4 | 98.01 | 98.78 | 97.24 | 29060978 | 29709080 | RM317 | RM3474 | 1.54 | 648102 |  | 19 | 2 | RN |
| M-QTL 5.1 | 5 | 78 | 75.72 | 80.27 | 19591187 | 20485804 | W653 | R3103 | 4.55 | 894617 |  | 121 | 2 | RN, RL |
| M-QTL 5.2 | 5 | 87.4 | 82.2 | 92.6 | 21064330 | 22184372 | C308 | RM5642 | 10.4 | 1120042 |  | 155 | 1 | DRR |
| M-QTL 6.1 | 6 | 31.79 | 26.95 | 36.64 | 5096744 | 6663826 | RM314 | RM8270 | 9.69 | 1567082 |  | 207 | 4 | RL, RGA, RN |
| M-QTL 7.1 | 7 | 83.3 | 81.26 | 85.33 | 23558551 | 24723154 | RM5508 | B2F2 | 4.07 | 1164603 |  | 178 | 1 | DRR |
| M-QTL 7.2 | 7 | 93.36 | 93.415 | 93.305 | 25301976 | 25457233 | C847 | RM473A | 0.11 | 155257 |  | 32 | 1 | RL |
| M-QTL 7.3 | 7 | 101.75 | 98.66 | 104.83 | 25312972 | 26354749 | RM3552 | RM3555 | 6.17 | #VALUE! |  | 157 | 1 | RN |
| M-QTL 7.4 | 7 | 108.8 | 106.37 | 111.22 | 26354749 | 28411532 | RZ978 | CDO38 | 4.85 | 2056783 |  | 222 | 1 | RSR |
| M-QTL 8.1 | 8 | 76.35 | 79.765 | 72.935 | 20483246 | 21444109 | RM1109 | S15156 | 6.83 | 960863 |  | 118 | 1 | RSR |
| M-QTL 9.1 | 9 | 60.75 | 61.58 | 59.92 | 15662573 | 16972710 | RM434 | W574 | 1.66 | 1310137 |  | 174 | 1 | RDR |
| M-QTL 9.2 | 9 | 63.7 | 63.04 | 64.35 | 17189773 | 17813881 | E61594S | RM2190 | 1.31 | 624108 |  | 99 | 1 | MRL |
| M-QTL 9.3 | 9 | 83.55 | 83.79 | 83.31 | 21032667 | 21299440 | S10578 | C570 | 0.48 | 266773 |  | 58 | 1 | RSAr |
| M-QTL 9.4 | 9 | 92.8 | 90.35 | 95.24 | 21856424 | 22504524 | C506 | RZ792 | 4.89 | 648100 |  | 124 | 2 | RN, RL |
| M-QTL 10.1 | 10 | 56.7 | 54.45 | 58.94 | 18963951 | 19762209 | E31548S | RM3019 | 4.49 | 798258 |  | 136 | 1 | RGA |
| M-QTL 11.1 | 11 | 80.3 | 76.81 | 83.78 | 18628574 | 20953256 | M244 | P64 | 6.97 | 2324682 |  | 244 | 2 | DRR, RL |
| M-QTL 11.2 | 11 | 86.37 | 87.405 | 85.335 | 20584994 | 21425287 | E1126S | C189 | 2.07 | 840293 |  | 29 | 3 | DRR, RN |

- **References**

Cairns, J.E., Audebert, A. Mullins, C.E. Price A.H. (2009). Mapping quantitative trait loci associated with root growth in upland rice (Oryza sativa L.) exposed to soil water-deficit in fields with contrasting soil properties. Field Crops Research 114 (2009) 108–118.

Catolos M, Sandhu N, Dixit S, Shamsudin NAA, Naredo MEB, McNally KL, Henry A, Diaz MG, Kumar A (2017) Genetic Loci Governing Grain Yield and Root Development under Variable Rice Cultivation Conditions. Plant Science. doi: 10.3389/fpls.2017.01763.

Emrich , K., Price, A., Piepho H. P. (2008). Assessing the importance of genotype 3 environment interaction for root traits in rice using a mapping population III: QTL analysis by mixed models. Euphytica. 161:229–240.

Gareth J. Norton ئ Adam H. Price (2009). Mapping of quantitative trait loci for seminal root morphology and gravitropic response in rice. Euphytica (2009) 166:229–237.

Hemamalini GS, Shashidhar HE, Hittalmani S. Molecular marker assisted tagging of morphological and physiological traits under two contrasting moisture regimes at peak vegetative stage in rice. Euphytica. 2000; 112:69–78.

Kitomi Y, Nakao E, Kawai S, Kanno N, Ando T, FukuokaSh, Irie K, Uga Y (2018) Fine Mapping of QUICK ROOTING 1 and 2, Quantitative Trait Loci Increasing Root Length in Rice. Genes 727-735.

Li H, Durbin R. 2009. Fast and accurate short read alignment with Burrows–Wheeler transform. Bioinformatics 25, 1754–1760.

MacMillan K, Emrich K, Piepho H-P, Mullins CE, Price AH. Assessing the importance of genotype x environmental interaction for root traits in rice using a mapping population II: conventional QTL analysis. Theor Appl Genet. 2006; 113:953– 64.

Norton GJ, Aitkenhead MJ, Khowaja FS, Whalley WR, Price AH (2008). A bioinformatic and transcriptomic approach to identifying positional candidate genes without fine mapping; an example using rice root-growth QTLs. Genomics.92:344–52.

Price AH, Steele KA, Moore BJ, Barraclough PB, Clark LJ. A combined RFLP and AFLP linkage map of upland rice used to identify QTLs for root-penetration ability. Theor Appl Genet. 2000; 100:49–56.

Price AH, Steele KA, Moore BJ, Jones RGW (2002). Upland rice grown in soil filled chambers and exposed to contrasting water-deficit regimes. II. Mapping QTLs for root morphology and distribution. Field Crop Research. 76:25–43.

Shen L, Courtois B, McNally KL, Robin S, Li Z. (2001) Evaluation of near isogenic lines of rice introgressed with QTLs for root depth through marker-aided selection. Theor Appl Genet.103(1):75–83.

Srividhya A, Vemireddy LR, Ramanarao PV, Sridhar S, Jayaprada M, Anuradha G, Srilakshmi B, Reddy HK, Hariprasad AS, Siddiq EA (2011) Molecular Mapping of QTLs for Drought Related Traits at Seedling Stage under PEG Induced Stress Conditions in Rice. Plant Sciences 2: 190-201.

Steele KA, Price AH, Sashidhar HE, Witcombe JR. (2006) Marker-assisted selection to introgress rice QTLs controlling root traits into an Indian upland rice variety. Theor Appl Genet.112:208–21.

Uga Y, Kitomi Y, Ishikawa S, Yano M. (2015) Genetic improvement for root growth angle to enhance crop production. Breeding Science, 65: 111–119.

Uga Y, Okuno K, Yano M (2011) Dro1, a major QTL involved in deep rooting of rice under upland ﬁeld conditions. Experimental Botany 62: 2485-94.

Uga Y, Okuno K, Yano M (2010) Fine mapping of Sta1, a quantitative trait locus determining stele transversal area, on rice chromosome 9. Mol Breeding 26: 533–538.

Uga Y, Sugimoto K, Ogawa S, Rane J, Ishitani M, Hara N, Kitomi Y, Inukai Y, Ono K, Kanno, N, Inoue H, Takehisa H, Motoyama R, Nagamura Y, Wu J, Matsumoto T, Okuno K, Yano M (2013) Control of root system architecture by Deeper Rooting1 increases rice yield under drought conditions. Nature Genetics 45: 1097-1106.

Uga, Y, Okuno K, Yano M (2008) QTLs underlying natural variation in stele and xylem structures of rice root. Breed. Sci 58: 7–14.

Venuprasad R, Shashidhar HE, Hittalmani S, Hemamalini GS (2002) Tagging quantitative trait loci associated with grain yield and root morphological traits in rice (Oryza sativa L.) under contrasting moisture regimes. Euphytica 128: 293–300.

Veyrieras, J.-B., Goffinet, B. & Charcosset, A. (2007). MetaQTL: a package of new computational methods for the meta-analysis of QTL mapping experiments. BMC bioinformatics 8(1): 49.

Zhang WP, Shen XY, Wu P, Hu B, Liao CY. (2001b) QTL and epistasis for seminal root length under a different water supply in rice. Theor. Appl. Genet. 103:118–23.

Zheng B, Yang L, Mao CZ, Zhang WP, Wu P (2006) QTLs and Candidate Genes for Rice Root Growth Under Flooding and Upland Conditions. Actu Geneticu Sinica 33 (2): 141-151.

Zheng BS, Yang L, Zhang WP, Mao CZ, Wu YR, Yi KK, et al. (2003) Mapping QTLs and candidate genes for rice root traits under different water-supply conditions and comparative analysis across three populations. Theor Appl Genet.107:1505–15.

Zheng H.G., Babu R.C., M.S Md, L Pathan, Ali, and. Nguyen H.T (1998). Quantitative trait loci for root-penetration ability and root thickness in rice: Comparison of genetic backgrounds. Genome 43: 53–61.
